# Tracing the biosynthetic origin of limonoids and their functional groups through stable isotope labeling and inhibition in neem tree (*Azadirachta indica*) cell suspension

**DOI:** 10.1186/s12870-018-1447-6

**Published:** 2018-10-11

**Authors:** Thiagarayaselvam Aarthy, Fayaj A. Mulani, Avinash Pandreka, Ashish Kumar, Sharvani S. Nandikol, Saikat Haldar, Hirekodathakallu V. Thulasiram

**Affiliations:** 1 Chemical Biology Unit, Division of Organic Chemistry, CSIR-National Chemical Laboratory, Dr. Homi Bhabha Road, Pune, 411008 India; 2 Academy of Scientific and Innovative Research, AnusandhanBhawan, 2 Rafi Marg, New Delhi, 110 001 India; 3 Institute of Genomics and Integrative Biology, Council of Scientific and Industrial Research (CSIR), Mall Road, New Delhi, 110007 India

**Keywords:** Neem, ^13^C labeling, Tiglate and isovalerate biosynthesis, qPCR, In vitro plant cell culture, Triterpenoids and natural products

## Abstract

**Background:**

Neem tree serves as a cornucopia for triterpenoids called limonoids that are of profound interest to humans due to their diverse biological activities. However, the biosynthetic pathway that plant employs for the production of limonoids remains unexplored for this wonder tree.

**Results:**

Herein, we report the tracing of limonoid biosynthetic pathway through feeding experiments using ^13^C isotopologues of glucose in neem cell suspension. Growth and development specific limonoid spectrum of neem seedling and time dependent limonoid biosynthetic characteristics of cell lines were established. Further to understand the role of mevalonic acid (MVA) and methylerythritol phosphate (MEP) pathways in limonoid biosynthesis, Ultra Performance Liquid Chromatography (UPLC)- tandem mass spectrometry based structure-fragment relationship developed for limonoids and their isotopologues have been utilized. Analyses of labeled limonoid extract lead to the identification of signature isoprenoid units involved in azadirachtin and other limonoid biosynthesis, which are found to be formed through mevalonate pathway. This was further confirmed by treatment of cell suspension with mevinolin, a specific inhibitor for MVA pathway, which resulted in drastic decrease in limonoid levels whereas their biosynthesis was unaffected with fosmidomycin mediated plastidial methylerythritol 4-phosphate (MEP) pathway inhibition. This was also conspicuous, as the expression level of genes encoding for the rate-limiting enzyme of MVA pathway, 3-hydroxy-3-methyl-glutaryl-coenzyme A reductase (HMGR) was comparatively higher to that of deoxyxylulose-phosphate synthase (DXS) of MEP pathway in different tissues and also in the in vitro grown cells. Thus, this study will give a comprehensive understanding of limonoid biosynthetic pathway with differential contribution of MVA and MEP pathways.

**Conclusions:**

Limonoid biosynthesis of neem tree and cell lines have been unraveled through comparative quantification of limonoids with that of neem tree and through ^13^C limonoid isotopologues analysis. The undifferentiated cell lines of neem suspension produced a spectrum of C-seco limonoids, similar to parental tissue, kernel. Azadirachtin, a C-seco limonoid is produced in young tender leaves of plant whereas in the hard mature leaves of tree, ring intact limonoid nimocinol accumulates in high level. Furthermore, mevalonate pathway exclusively contributes for isoprene units of limonoids as evidenced through stable isotope labeling and no complementation of MEP pathway was observed with mevalonate pathway dysfunction, using chemical inhibitors.

**Electronic supplementary material:**

The online version of this article (10.1186/s12870-018-1447-6) contains supplementary material, which is available to authorized users.

## Background

**Fig. 1:**
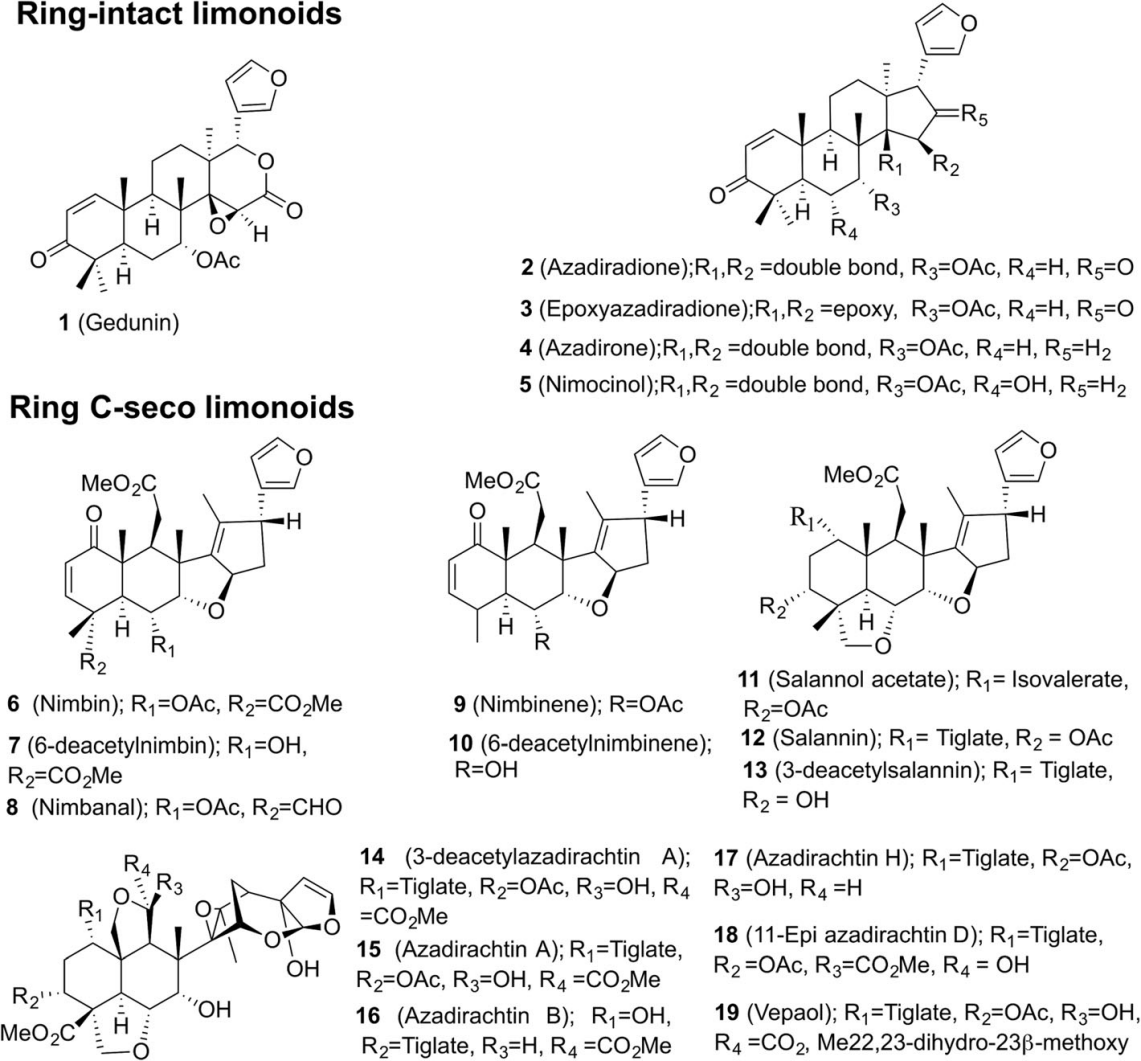
Chemical structures of ring intact and ring C- seco limonoids dealt with in this study

*Azadirachta indica* A. Juss (Indian Lilac), a member of Mahogany family (Meliaceae) is a medicinal tree of Indian subcontinent. Different parts of the tree have been used in traditional Ayurvedic and Unani medicine for the treatment of myriads of human ailments [1]. Various parts of neem tree serves mankind since time immemorial with its bountiful medicinal properties, of these the neem seed has been used widely as a natural insecticide in agricultural practice. The most active and characteristic compound of neem tree, azadirachtin A (Fig. 1) is found in most of the tissues of neem tree, however its occurrence is rich in the seed kernel [2, 3]. Azadirachtin A, an effective insect growth deterrent, is found to be the best potential natural insecticide candidate identified so far from the plant sources, also possesses remarkable non-toxicity to vertebrates [2]. Unlike other insecticide, which exerts its effect on the nervous system of insects, azadirachtin was known to act on the endocrine system, thereby affecting the feeding behaviour, development, reproduction and metabolism in insects [2, 4]. Besides the insecticidal potential, azadirachtin A was found to possesses osteogenic activity and beneficial effects on bone [5]. Considering the broad-spectrum activity of this marvelous biopesticide, azadirachtin A, several studies have been reported for the optimization of increasing its productivity through cell suspension and hairy root cultures [6, 7]. Characterization of this highly oxygenated, complex molecule was startling in such a way that it paid impetus for the perseverance of the researchers from both biological and chemical fields. In particular, following the determination of correct structure of azadirachtin in 1985 [8], Ley and co-workers achieved the total synthesis of this molecule after two decades of efforts [9, 10].

Azadirachtin belonging to tetranor-triterpenoids, a triterpene class of secondary metabolites is a well-known neem limonoid [2, 11, 12]. Over 150 limonoids have been isolated and characterized from different parts of the neem tree [11, 12]. The pharmaceutical applications of various plant parts and the individual neem limonoids has been discerned indeed [1, 11, 12]. To exemplify, the anti-viral efficacy of neem tree is evident from the studies on activity of neem leaf and bark extracts against dengue and encephalitis viruses respectively [1, 13, 14]. The limonoid, gedunin possess potential anti-tumor, anti-malarial [15, 16] and anti-diabetic activity [17], azadiradione confers anti-inflammatory [18] and anti-diabetic effect [17]. Protective effect of salannin on gastric lesions and spermicidal effect of salannin, nimbin and nimbidinhas been identified [19]. The anti-angiogenic and anti-proliferative potential of nimbolide has been demonstrated extensively through various in vitro and in vivo studies [20–22]. Neem tree is a paradigmatic example of triterpenoids rich medicinal plant with each terpenoids being endowed with different medicinal properties. Besides the remarkable knowledge on the potential bioactivity of neem limonoids, very little is known about their biosynthetic pathway and that have enthralled our attention to study their biosynthesis.

Limonoid biosynthetic pathway deviates from the steroid biosynthesis at the formation of triterpene skeleton, through cyclization of 2, 3(*S*)-oxidosqualene catalyzed by triterpene synthase. Oxidosqualene is formed by epoxidation of squalene (30C), which is formed through non-head-to-tail, 1′-1 condensation of two units of farnesyl diphosphate (FPP) [3] catalyzed by squalene synthase. FPP is formed through the most common chain elongation reaction by the coupling of isopentenyl diphosphate (IPP) with its allylic diphosphate, dimethyl allyl diphosphate (DMAPP) or with geranyl diphosphate (GPP) [23–29]. In higher plants, biosynthesis of isoprenoids occurs through either of the two biosynthetic pathways: mevalonate pathway (MVA) or the methyl-erythritol phosphate pathway (MEP) or through a combination of both pathways [23, 25, 30]. Classical MVA pathway is localized in the cytosol of the plant cell, [25] whereas MEP pathway occurs in the plastids [26, 30, 31]. It is found that monoterpenes, diterpenes and tetraterpenes are synthesized through the plastidic MEP pathway [32, 33] and sesquiterpenes, sterols (triterpenes) through the cytosolic MVA pathway, however it varies between plants and under different physiological conditions [34–36]. Contradictory to this prevalence of dichotomy, there are evidences based on labeling studies that suggests the differential contribution of isoprene C5 units from either of the two pathways towards the biosynthesis of different metabolites due to traversing of isoprene units across the thylakoid membrane of plastids. Consecutive isoprenoid pathway inhibition and labeled precursor feeding experiments carried out in tobacco cells showed that exchange of the isoprene units biosynthesized through the two pathways occurs for the complementation of the other pathway which indeed gives flexibility for the plants to survive under the conditions of stress [37–41].

In neem tree, tetracyclic triterpene skeletal intermediate(s), en route to the formation of proto-limonoids through successive oxidation and rearrangement reactions [42]. The proto-limonoid further undergoes skeletal rearrangements and functionalization mediated by oxido-reductase and hydrolase enzyme systems to form higher limnoids such as basic and C-seco limonoids [42]. Depending on the skeletal modifications, limonoids can be subdivided into ring-intact (basic) and C-seco limonoids. Ring-intact limonoids encompass 4,4,8-trimethyl-17-furanylsteroidal skeleton such as azadirone and its derivatives. C-seco limonoids are generated by C-ring opening and further rearrangements thus producing nimbin, salannin and azadirachtins type of limonoids (Fig. 1) [3, 12]. Although intensive work has been carried out on the isolation, characterization, synthesis and bioactivity of limonoids, a very little is known about their biosynthesis and remains as a fact of speculation hitherto.

To gain insight into the isoprenoid biosynthetic pathway(s) involved in the contribution of isoprene units into limonoid skeleton, we carried out labeling experiment with [1-^13^C], [2-^13^C] glucose (Glc) isotopomers and its isotopologue, [1,6-^13^C] Glc in *Azadirachta indica* suspension cell cultures, which served as an experimental system for studying the biosynthesis of limonoids. We have utilized high resolution-tandem Mass spectrometry to study the limonoids produced in cell culture followed by the study of ^13^C labeling pattern of the limonoids obtained through feeding experiment. The dissection of the chemical complexity of limonoids by rendition through the isotopologues and isotopomers obtained with tandem MS was made possible because of high mass resolution and accuracy of MS. The isotope labeling experiments were carried out subsequent to the inhibition of isoprenoid biosynthetic pathways to study the complementation of MVA and MEP pathways for the biosynthesis of limonoids in neem cell culture. Spatial expression levels of genes involved in the rate-limiting step of the MVA and MEP pathways were studied in different tissues of neem tree. To further comprehend their regulatory roles during the inhibition of MVA pathway, the expression level of the same genes were examined through quantitative polymerase chain reaction (qPCR) experiments.

## Results

### Metabolic profiling of neem plantlets, callus and cell suspension culture

**Fig. 2:**
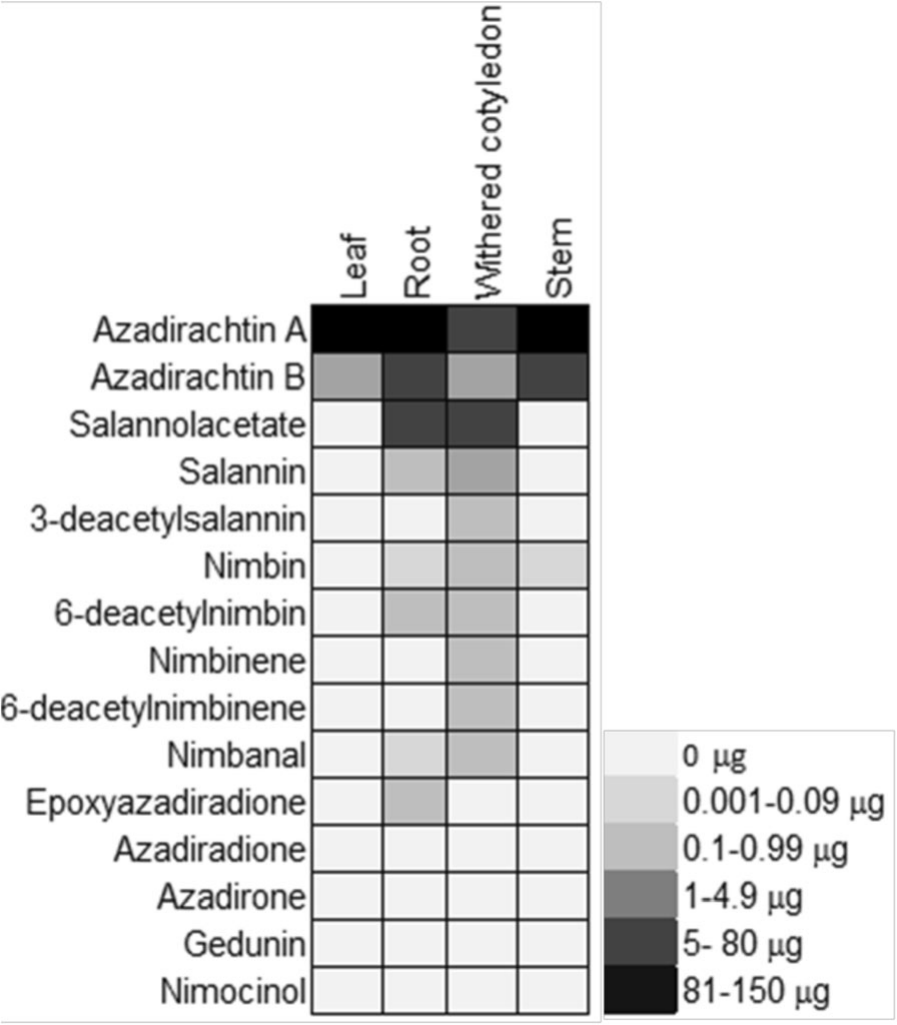
Targeted limonoid profile of in vitro grown neem seedling emerged from cotyledons

Neem plantlets and a stable cell suspension were established from the callus culture obtained from kernel, in order to profile and quantify the individual limonoids formed and to perform feeding experiments for tracing the limonoid biosynthetic pathway.The neem kernel started callusing in four weeks of inoculation and it was maintained by regular subculturing (Methods). Neem seedling was developed from the cotyledon from four weeks of inoculation. Limonoid profiling was carried out using UPLC-ESI(+)-HRMS based targeted profiling. Azadirachtin A was found to be the major limonoid identified in the different part of seedling followed by azadirachtin B (Fig. 2). Azadirachtin A present in the stem of the plant is 123 μg/g, 100 μg/g in root, 81 μg/g and 7 μg/g in leaf and withered cotyledons respectively.

**Fig. 3:**
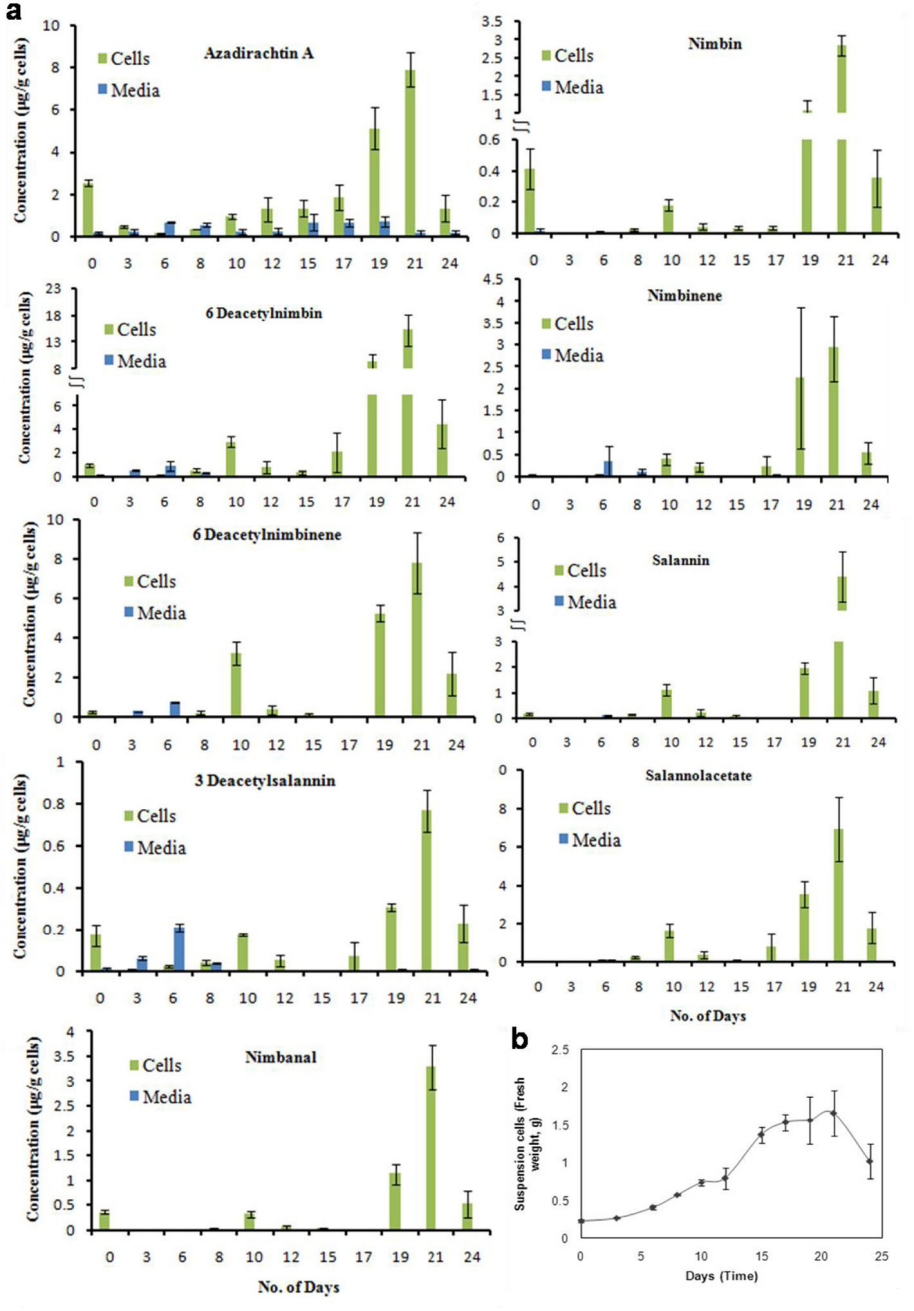
Growth of suspension cell cultures and time course study of limonoid biosynthesis. (**a**) limonoid content during the course of cellular growth (Blue represents the level of limonoids present media, green represents the level of limonoids present in suspension cell biomass). (**b**) Growth curve obtained with cell suspension

The studies indicated that both ring intact (basic) and C-seco limonoids were identified in the newly formed callus however, in subsequent subcultures, the levels of ring intact limonoids decreased. At the end of three subcultures, the basic limonoids were not detected. C-seco limonoids such as azadirachtin A, salannin, salannolacetate, 3-deacetylsalannin, nimbin, 6-deacetylnimbin, nimbinene, 6-deacetylnimbinene, nimbanal were found in callus and suspension culture of neem cells as identified (Additional file 1: Figure S1) and quantified based on extracted ion chromatogram. Callus inoculum of 0.2 g, was dispersed in 5 ml of liquid MS media to initiate cell suspension culture, which showed a typical growth curve (Fig. 3b). A lag phase of 5 days was observed from the day of inoculation, followed by exponential phase for the next 5 days. Following the exponential phase, linear growth phase was observed for 9 days and the stationary phase started from day 20. Nine limonoids identified in the suspension culture were quantified over the time course of cellular growth (Fig. 3a). Limonoid accumulation in cells was recorded highest in the stationary phase of growth. Among the nine C-seco limonoids identified, level of 6-deacetylnimbin and 6-deacetylnimbinene were higher (17 μg/g and 8 μg/g of cells respectively) among the tetranor- and pentanor-triterpenoid group of limonoids, respectively in the cell suspension culture. In the media, the limonoids were identified in traces, and does not increase over the time (Fig. 3a).

### Characterization of suspension cell cultures for limonoid biosynthesis

**Fig. 4:**
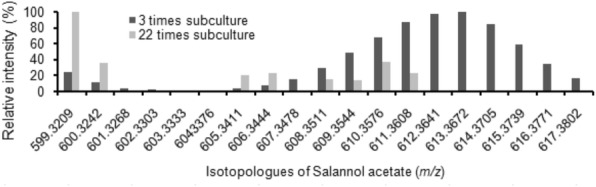
Characterization of suspension cells for limonoid biosynthesis. Comparison of salannolacetate isotopologues formed between cell suspension obtained from callus of different subculturing stages (3 and 22) after growing the cells in the presence of D- [1,6-^13^C] Glc

UPLC-ESI-MS analysis of the suspension cells derived from callus, which has been subcultured 22 times, indicated the drastic decrease in the levels of limonoids compared to the one of third stage. This was also evidenced from the [1,6-^13^C] Glc labeling study carried out with the suspension cells obtained at different subculturing stage of callus. Limonoid isotopologues formed due to incorporation of ^13^C label from [1,6-^13^C] Glc fed cell suspension obtained from third sub-culturing stage callus was comparatively high however no ^13^C incorporated limonoids detected in the cell culture derived from callus which has been sub-cultured 22 times (Fig. 4). Feeding experiments clearly indicated that, there is a gradual drop in the biosynthesis of limonoids in due course of subculturing of callus. Labeled limonoid were not detected in the media, hence indicating that the traces of limonoids identified in the media (Fig. 3a) may be from the ruptured cells formed during the generation of suspension from callus. These results show that limonoids are synthesized and stored inside the suspension cells.

### Biosynthesis of limonoids: Feeding experiment with different ^13^C labeled D-glucose tracers

**Fig. 5:**
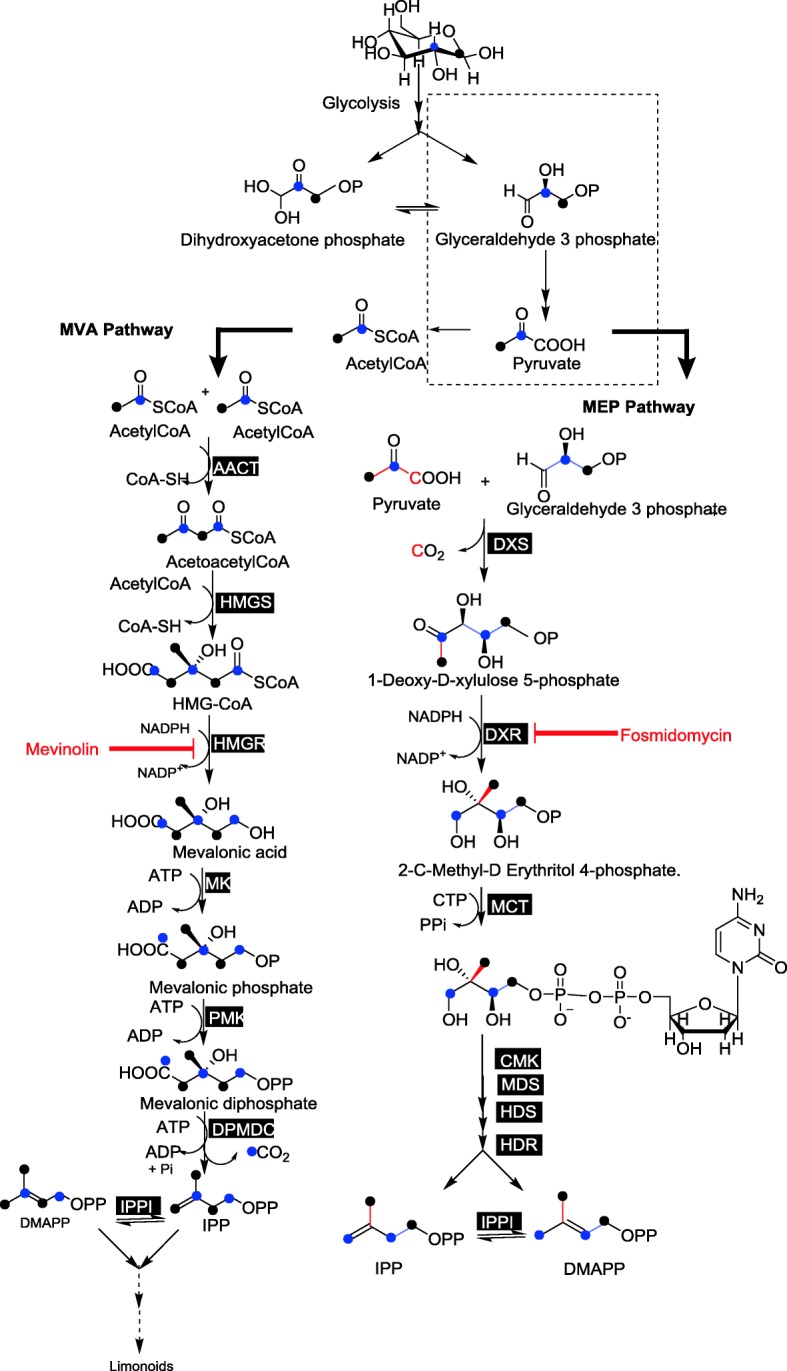
Tracing the flow of ^13^C from glucose through MVA and MEP pathway for the biosynthesis of isoprene unit. The black dot representsthe ^13^C carbon derived from 1-^13^C label /1,6 -^13^C of D-glucose and the blue one from 2-^13^C label of D-glucose. Mevinolin and fosmidomycin are the inhibitors for HMGR and DXR, respectively. AACT, acetoacetyl-CoA thiolase; HMGS, 3-hydroxy-3 methyl glutaryl coenzyme A synthase; HMGR, 3-hydroxy-3-methyl glutaryl coenzyme A reductase; MK, mevalonate kinase; PMK, 5-phosphomevalonate kinase; DPMDC, diphosphomevalonate decarboxylase; IPPI, isopentenyl diphosphate isomerase; DXS,1-deoxy-D-xylulose-5-phosphate synthase; DXR, 1-deoxy-D-xylulose-5-phosphate reductoisomerase; MCT, 2-C-methyl-D-erythritol-4 phosphate cytidyltransferase; CMK, 4-(Cytidine 5′-diphospho)-2-C-methyl-D-erythritol kinase; MDS, 2-C-methyl-D-erythritol 2,4-cyclodiphosphate synthase; HDS, 4-hydroxy-3-methylbut-2-enyl diphosphate synthase; HDR, 4-hydroxy-3-methylbut-2-enyl diphosphate reductase

The isoprene units synthesized through MVA and MEP pathway will have different labeling patterns when cell suspension culture is fed with ^13^C labeled D-Glucose (Fig. 5). This principle is taken further to investigate the contribution of isoprene units formed through MVA and MEP pathways towards the biosynthesis of limonoids, feeding experiment was carried out with different ^13^C labeled Glucose (Glc) tracers. 24 h old neem suspension cultures were grown in liquid MS media were re-suspended in MS media containing [1-^13^C] Glc, [2-^13^C] Glc or [1,6-^13^C] Glc. The control experiment was carried out using MS media containing unlabeled glucose under similar conditions. At the end of 19 days of incubation, cell biomass were extracted for limonoids and subjected to UPLC-HRMS analysis. Azadirachtin A, nimbin, 6-deacetylnimbin, nimbinene, 6-deacetylnimbinene, salannin, 3-deacetylsalannin, salannolacetate and nimbanal were identified in the cell suspension generated ^13^C isotopologues, which showed normal distribution pattern in their relative intensity. Azadirachtin A, salannin, salannolacetate, 6-deacetylnimbinene were identified in abundance with high intensity of isotopologues. The isotopologues varied in their number and intensity for each of the studied limonoid, upon labeling with different positional ^13^C-Glc tracers. High resolution MS/MS serves as a powerful technique, and helped us for the determination of intramolecular distribution of ^13^C in the isotopologues of the metabolite. Natural abundance of isotopologues was observed in MS for limonoids obtained from unlabeled control cell suspension (Additional file 1: Figure S3c, S3i, S3o, S3u). However, in nature, ^13^C abundance is only 1.1% and thus the signal for lower mass fragments obtained during MS/MS spectra of limonoids are predominantly from the monoisotopic ions containing its most abundant isotope (^12^C). However, in ^13^C enrichment experiments, molecular ions obtained in MS are represented by different isotopologues.

**Fig. 6:**
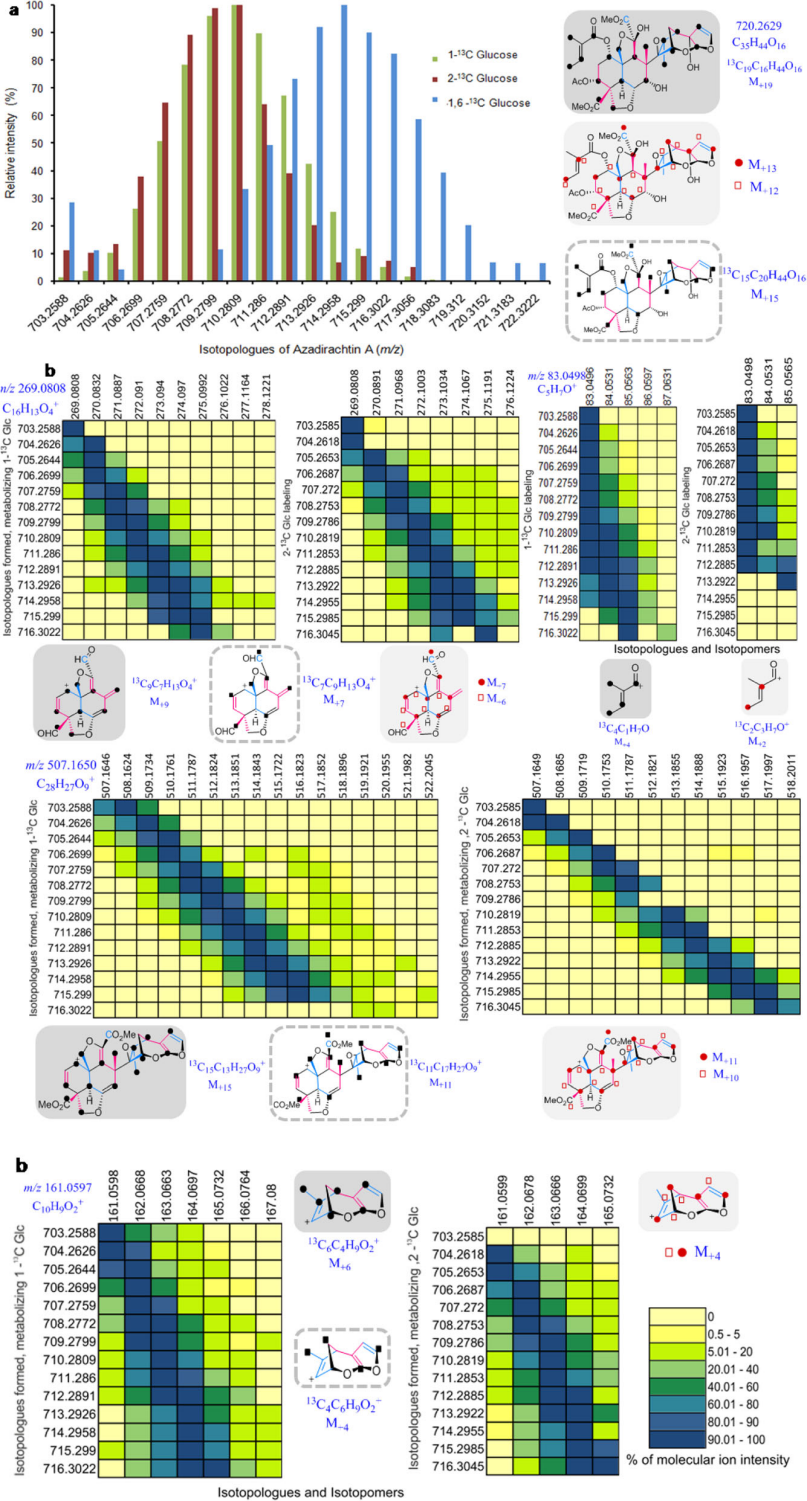
Distribution of azadirachtin A isotopologues and isotopomers obtained from ESI-MS and MS/MS analysis. (**a**) Comparison of relative intensity of isotopologues of protonated ion adduct [M-H_2_O + H]^+^ of azadirachtin A obtained from independent labeling experiments with [1-^13^C], [2-^13^C], [1,6-^13^C] Glc. (**b**) Heatmap of isotopologue of azadirachtin A in the mass spectra vs relative intensity of each of the isotopologues for individual fragments (*m/z* 83.0498, 161.0597, 269.0808, 507.1650 derived from different part of the skeleton are represented) obtained when subjected to tandem MS at collision energy of 20%. The structure of the molecule and its fragments are given in grey boxes for the molecule denoting its ^13^C pattern of formation through MVA pathway and dashed line boxes for its formation through MEP pathway. The black dots and squares represent the position of ^13^C carbon incorporated from [1-^13^C] Glc / [1,6-^13^C] Glc whereas the red dots corresponding to those from [2-^13^C] Glc

UPLC-HRMS analysis of azadirachtin A obtained from the unlabeled, control cell suspension showed a protonated parent molecular ion, *m/z* 703.2588 ([M-H_2_O + H]^+^). Neem cell suspension enriched by feeding [1-^13^C] Glc and [1,6-^13^C] Glc yielded with 14 and 19 isotopologues, respectively (*m/z* 703.2588 to 717.3056 and 722.3222) (Figs. 6a, 9d, Additional file 1: Figure S3a-S3e). [2-^13^C] Glc labeling experiment was conducted to corroborate further, the results obtained with [1-^13^C] and [1,6-^13^C] Glc. The ^13^C enrichment of the suspension with [2-^13^C] Glc gave rise to 14 mass units to the azadirachtin A parent ion (Fig. 6a, S3f).

Mass spectra of 6-deaceylnimbinene from the control showed parent molecular ion [M + H]^+^ of *m/z* 441.2264 (Additional file 1: Figure S3 g, S3i). Incorporation of 11 and 14 ^13^C atoms was reflected from the comparison of unlabeled molecular ion with that of [1-^13^C] Glc and [1,6-^13^C] Glc labeling experiment, respectively (*m/z* 441.2264 to 452.2632 and *m/z* 441.2264 to 455.2730). Whereas, ^13^C enrichment with [2-^13^C] Glc labeling culminates with 10 isotopologues (Additional file 1: Figure sS3 g-S3 l, S5a).

Parent molecular ion [M + H]^+^, *m/z* 597.3050 was observed for salannin in the control cell culture whereas gradient increase of 14 (*m/z* 597.3050 to 611.3516) and 16 mass units (*m/z* 597.3050 to 613.3582) were generated from [1,6-^13^C] Glc as isotopic tracer. With the use of [2-^13^C] Glc as tracer, 12 isotopologues were observed (Additional file 1: Figure S3 m-S3r, S6a).

**Fig. 7:**
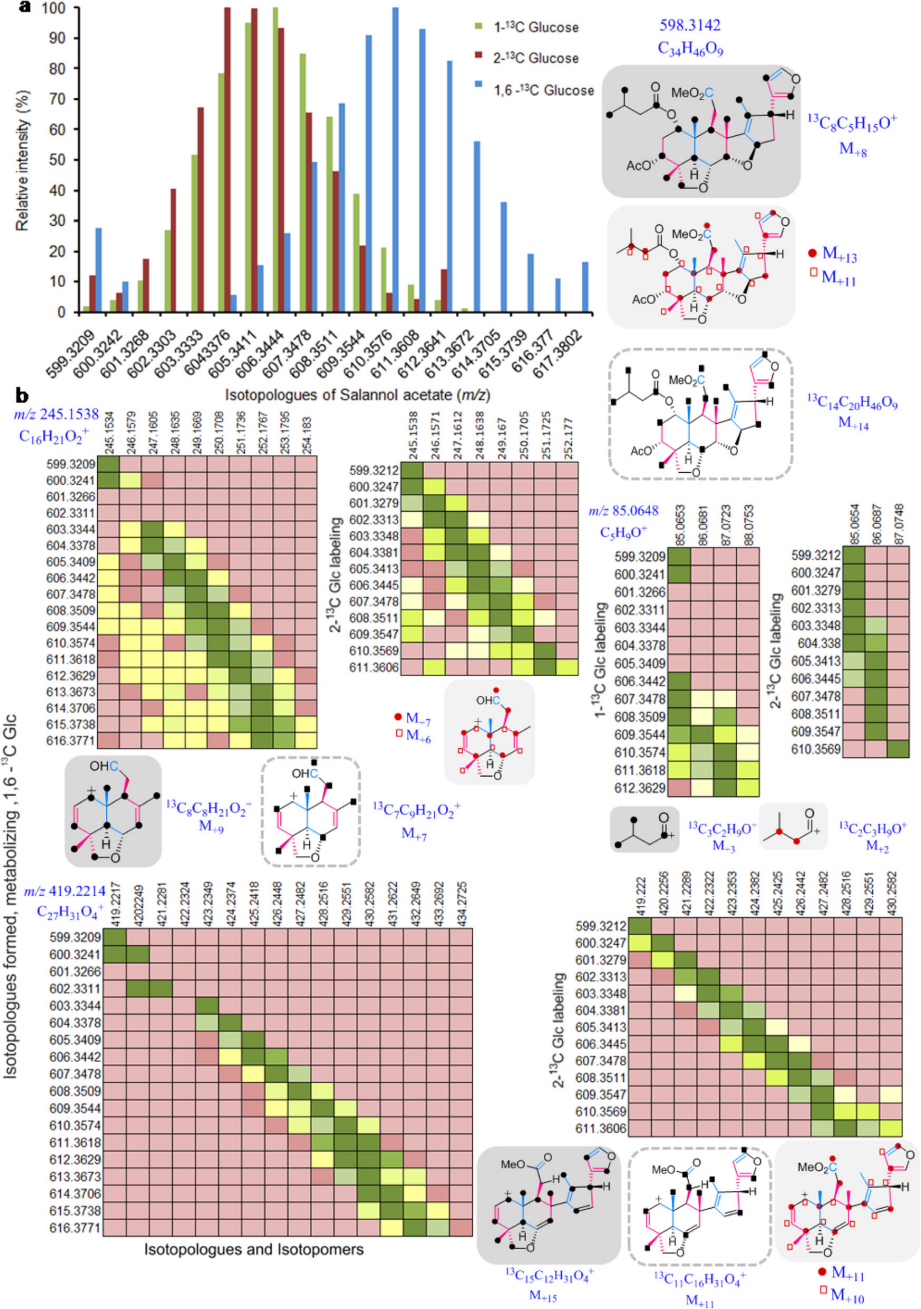
Relative intensity of salannolacetate isotopologues formed through labeling study with different tracers of Glucose ([1-^13^C], [2-^13^C] and [1,6-^13^C]) and the number of ^13^C carbon incorporation evident from tandem MS analysis of the isotopologues. (**a**) MS of salannolacetate isotopologues formed through different ^13^C Glc feeding experiments (**b**) MS/MS distribution of isotopologues of the fragments (*m/z* 85.0648, 245.1538, 419.2214). Heatmap at the left corresponding to isotopologues formed through either [1-^13^C] or [1,6-^13^C] Glucose and the one at the right correspond to isotopologues formed through [2-^13^C] Glc. (M represents the *m/z* of parent ion of the molecular ion/ fragment)

Salannolacetate from the unlabeled culture generated parent ion [M + H]^+^ of *m/z* 599.3209 where as the labeled cell biomass produced 14 and 18 isotopologues upon [1-^13^C] and[1,6-^13^C] Glc labeling, respectively (*m/z* 599.3209 to 613.3672 and 617.3802 respectively). In case of [2-^13^C] Glc labeling, isotopologues with 13 mass units were observed in addition to the parent ion (*m/z* 599.3209 to 612.3672) (Fig. 7a, Additional file 1: Figure S3 s-S3x).

In order to dissect the position of ^13^C in the above limonoid skeletons, tandem MS fragmentation of the limonoid obtained from the labeled and unlabeled culture need to be compared. Structure-fragment relationship for C-seco limonoids such as salannin, salannolacetate and 6-deacetylnimbinene has been established in our previous study. However, there is no report on ESI based tandem mass spectrometric characterization of azadirachtin. Herein, we report structure-fragment relationships for azadirachtin using UPLC-ESI-quadrupole/orbitrap-MS. These data will be utilized for tracing their biosynthetic pathway for these limonoids in neem suspension culture fed with [1-^13^C] Glc, [2-^13^C] Glc or [1,6-^13^C] Glc.

### Tandem mass spectrometric characterization of azadirachtin a

To understand the fragmentation pattern for azadirachtin A, we have subjected azadirachtin A and its derivatives such as 3-deacetylazadirachtin A, azadirachtin B, vepaol, azadirachtin H, 11-epi-azadirachtin D to UPLC-ESI(+)-quadrupole/orbitrap-MS/MS study. Mass spectral traces of azadirachtin A and its derivatives did not show molecular ion peak ([M + H]^+^) however had the fragment ion with a loss of water molecule, i.e. [M-H_2_O + H]^+^ and sodium adduct [M + Na]^+^. [M-H_2_O + H]^+^ adduct was considered as parent ion and was subjected to MS/MS fragmentation at various NCEs such as 10, 15, 20 and 25% (Additional file 1: Figure S2a). At 20% NCE, both the high and low molecular weight fragments were equally distributed in intensity and hence used to understand the fragmentation pattern for azadirachtin. The signature spectral fragments (daughter ions) obtained after fragmentation of each azadirachtin derivative at 20% NCE and their corresponding relative intensity have been populated in the table (Additional file 1: Table S1).

Probable fragmentation pathway for the formation of daughter ions of azadirachtin A, when the parent ion, [M-H_2_O + H]^+^ *m/z* 703.2596 was subjected to MS/MS at the NCE of 20% has been schematically represented (Additional file 1: Figure S2b). The mass fragmentation of parent molecular ion, *m/z* 703.2596, lead to the formation of fragment *m/z* 685.2491 [M-2H_2_O + H]^+^, due to loss of a molecule of H_2_O and it was identified at the relative intensity of 2.6%. This fragment *m/z* 685.2491, undergoes several neutral losses to generate fragments in the high molecular range at *m/z* 625.2280 [M-2H_2_O-AcOH+H]^+^ (3.2%), 607.2174 [M-3H_2_O-AcOH+H]^+^ (1.2%), 585.1967 [M-2H_2_O-Tiglic acid+H]^+^, 567.1861 [M-3H_2_O-Tiglic acid+H]^+^ (62.2%), 525.1755 [M-2H_2_O-Tiglic acid-AcOH+H]^+^ (30.4%), and 507.1650 [M-3H_2_O-Tiglic acid-AcOH+H]^+^ (85.6%). Hydrolysis of one of the ester group and/or removal of one or two CH_2_O molecules from *m/z* 507.1650 gave rise to the fragments *m/z* 493.1493 [M-3H_2_O-Tiglic acid-AcOH-CH_3_ + H]^+^ (15%), 463.1387 [M-3H_2_O-Tiglic acid-AcOH-CH_2_O-CH_3_ + H] (28.9%) and 447.1438 [M-3H_2_O-Tiglic acid-AcOH-2CH_2_O + H]^+^ (50%), respectively. A low relative ion intensity (13%) fragment of *m/z* 555.1861 [M-2H_2_O-Tiglic acid-CH_2_O + H]^+^ was formed after removal of a H_2_O, tiglic acid and a CH_2_O group from the parent ion.

The lower molecular weight fragments of *m/z* 329.1020 and *m/z* 269.0808 corresponded to the decalin portion (A and B ring) of azadirachtin molecule formed after the detachment of hydrofuran acetal moiety from the fragments *m/z* 507.1650 and *m/z* 447.1438 respectively. Finally, the hydrofuran acetal moiety of azadirachtin was identified as fragment of *m/z* 161.0597 with highest intensity of 100%. The fragments of *m/z* 400 to 600 range were possibly formed through protonation-mediated cleavage of tigloyl ester bond and thereby release of tiglate daughter ion (*m/z* 83.0491) with 76.4% intensity. This was in line with the fragmentation pattern obtained with salannin and 3-deacetylsalannin molecules [43].

3-deacetylazadirachtin A molecule differs from azadirachtin A by the absence of acetyl group at 3-C of ring A. The parental molecular ion, *m/z* 661.2491 [M-H_2_O + H]^+^ was subjected to fragmentation at NCE of 20% and each daughter fragment obtained were probed for its molecular formula and corresponding structure. The daughter ions of *m/z* 643.2382 [M-2H_2_O + H]^+^, 625.2281 [M-3H_2_O + H]^+^ and 607.2174 [M-4H_2_O + H]^+^ were obtained after successive removal of H_2_O molecules following protonation. Fragments such as *m/z* 525.1758, 507.1655, 465.1543, 447.1436 and 329.1013 obtained were in common with azadirachtin A fragmentation pattern (Additional file 1: Table S1).

Vepaol possesses a methoxy group in the hydrofuran moiety of azadirachtin A, thereby contributing to the increase in molecular weight of 32 Da. Mass spectra of vepaol showed the presence of three protonated ion peaks with *m/z* 735.2867, 721.2705, and 703.2598 which are formed through removal of either H_2_O molecule or methoxy group or both (Additional file 1: Table S1). All these three parental ions were subjected to fragmentation at NCE 20% and the resulting daughter ions for each of them were studied. Most of the daughter fragments of azadirachtin A were observed in common with vepaol, as it possess same structure of azadirachtin A after the removal of methoxy group (Additional file 1: Table S1).

Azadirachtin B is devoid of hydroxyl group at 11-C, tiglate at 1-C and 3-C acetyl functionality is replaced with tiglate group in comparison to azadirachtin A. The removal of tiglate moiety from the parent ion *m/z* 645.2542 ([M-H_2_O + H]^+^) yielded the fragment *m/z* 545.2017 [M-H_2_O-Tiglic acid+H]^+^. The fragments, *m/z* 509.1806 [M-3H_2_O-Tiglic acid-AcOH+H]^+^, 527.1912 [M-2H_2_O-Tiglic acid-AcOH+H]^+^ and 271.0965 obtained were on par with *m/z* 507.1650, *m/z* 525.1755 and *m/z* 269.0809 of azadirachtin A, respectively (Additional file 1: Table S1) due to the absence of double bond between 9-C and 11-C.

Azadirachtin H is a decarboxymethyl derivative of azadirachtin A at 11-C position and MS/MS at NCE 20% fragmented the parent ion to greater extent such that only lower *m/z* fragments formed from decalin portion were seen (Table S1). Higher mass fragments such as *m/z* 545.2017 [M-H_2_O-Tiglic acid+H]^+^, 509.1806 [M-3H_2_O-Tiglic acid+H]^+^, 467.1700 [M-2H_2_O-Tiglic acid-AcOH+H]^+^ and 449.1595 [M-3H_2_O-Tiglic acid-AcOH+H]^+^ were obtained at ≤15% NCE.

11-epi-azadirachtin D lacks one of the two methylester groups of azadirachtin A and produced two types of protonated ions with *m/z* 659.2704 [M-H_2_O + H]^+^ and 641.2598 [M-2H_2_O + H]^+^. The following fragments *m/z* 285.1121 and 345.1333 were formed from the decalin portion of the molecule (Table S1). The subsequent removal of the functional groups such as hydroxyl, tigloyl, acetyl groups generated fragments such as *m/z* 541.2068 [M-2H_2_O-Tiglic acid+H]^+^, 523.1963 [M-3H_2_O-Tiglic acid+H]^+^, 481.1857 [M-2H_2_O-Tiglic acid-AcOH+H]^+^, 463.1751 [M-3H_2_O-Tiglic acid-AcOH+H]^+^, *m/z* 451.1751 [M-2H_2_O-Tiglic acid-AcOH-CH_2_O + H]^+^ from both the parent ions.

The fragment *m/z* 161.0597 was identified in fragmentation pattern of most of the azadirachtin derivatives. The fragments, *m/z* 161.0597 and *m/z* 83.0491 corresponding to hydrofuran acetal moiety and tiglate group. Therefore either one acts as a signature fragment for azadirachtin class of limonoids from neem tree.

Therefore, structure-fragment relationship of azadirachtin A, thus obtained will not only help to infer the position of ^13^C labels incorporated into it, but also serves as a sensitive method for identification of azadirachtin in environmental as well as biological samples. We have studied already, the structure-fragment relationship for C-seco limonoids such as salannin, salannolacetate, nimbin, nimbinene, 6-deacetylnimbinene and 6-deacetylnimbin, isolated and purified from neem oil [43]. These results have been used for deducing the pattern of ^13^C incorporation into the same limonoids and their isotopologues identified in our feeding experiments.

### Tracing the limonoid biosynthesis through neem cell suspension feeding experiments

After understanding the fragmentation pattern for azadirachtin A, we subjected its isotopologues obtained through incorporation of ^13^C labels from [1-^13^C], [1,6 -^13^C] and [2-^13^C] Glc feeding experiments, in order to identify the position of each ^13^C label in the skeleton of limonoids. MS/MS traces obtained with NCE of 20% for each of the limonoid isotopologue were compared with that of unlabeled fragments to establish the number of ^13^C labels present in that fragment (Figs. 6b, 7b, Additional file 1: Figure S4, S5b, S6b). The ^13^C-enriched fragmentation pattern thus obtained was further subjected to retro-biosynthetic label tracing approach by applying to squalene intermediated model of triterpene origami for azadirachtin biosynthesis, to probe the origin of each carbon through MVA or/and MEP pathways. According to the biosynthetic pathway of triterpene origami, tetranor- and pentanor-limonoids are formed through loss of four and five isoprenogenic carbons from the C_30_ triterpene skeleton and gain of further non-isoprenogenic carbon as functional groups.

It is well established in literature [25, 27], that labeling with [1-^13^C] Glc culminates with IPP or DMAPP labeled at C-2, C-4, C-5 when they are formed through MVA pathway whereas ^13^C will be incorporated into isoprene units at C-1 and C-5 in case of their biosynthesis through MEP route (Fig. 5). In case of [1,6-^13^C] Glc labeling, two 2-^13^C acetyl-CoA are generated from a Glc molecule unlike that of one unlabeled and one 2-^13^C acetyl-CoA formed through [1-^13^C] Glc as tracer. Metabolism of acetyl-CoA formed from [2-^13^C] Glc result in labeling of isoprene units at C-1 and C-3 through MVA pathway, whereas at C-2 and C-4 in case of MEP pathway (Fig. 5). Based on the above information on labeling pattern of isoprene, and considering the number of incorporated ^13^C carbons on each of the fragments from tandem MS analysis, the pattern was fitted into the above origami model. Isotopologues generated for each of the signature fragment of labeled limonoids such as 6-deacetylnimbinene, salannin, salannolacetate and azadirachtin A are discussed in detail.

The fragment, *m/z* 147.0804 possess characteristic signature of C-seco limonoids such as salannolacetate, salannin, 3-deacetylsalannin, nimbin, 6-deacetylnimbin, nimbinene, 6-deacetylnimbinene and nimbanal corresponding to the C- and D-ring with 17-furan moiety, as per our earlier study [43]. This fragment encompasses two isoprene units and 1 carbon from third isoprene. Feeding experiment with [1-^13^C]/ [1,6-^13^C] Glc resulted in six isotopologues, possibly through three ^13^C in each of the two isoprenes thereby, ignoring the participation of MEP, as only two carbons of isoprene get labeled via latter pathway. Whereas [2-^13^C] Glc experiment did not provide any useful insight into tracing the pathway as two ^13^C labeled isoprenes can be obtained through both pathways however at different positions resulting in increase in 4 mass units to the fragment.

Amongst the nine limonoids identified in the culture, nimbinene and 6-deacetylnimbinene constitute the class of penta-nortriterpenoids. It is important to note that the maximum number of ^13^C incorporated into 6-deacetylnimbinene skeleton are 14 through [1,6-^13^C] Glc labeling, corroborating to the fact that three ^13^C may be lost during the excision of the terminal four carbons in the formation of proto-limonoid and another carbon from A ring during the formation of penta-nortriterpenoids (Additional file 1: Figure S5). The signature fragment, *m/z* 187.1119 corresponding to the decalin moiety was identified exclusively in this class of limonoids and this decalin moiety are constituted from first 3 isoprene units of squalene epoxide. Comparison of control and labeled culture, revealed the formation of 8 isotopologues in [1-^13^C] Glc labeling. If, it had been formed through MEP route, only five ^13^C labels would be incorporated into it. In case of [2-^13^C] Glc labeling, five mass units were observed however, labeling through either of the pathways may result in same number of labels into this fragment. Further, the fragment of *m/z* 409.2010, obtained after the removal of MeOH from the methylester group from the parent ion represents 6-deacetylnimbinene skeleton, composed of 4 complete isoprene units and 2 incomplete units which has lost its carbons from the triterpene skeleton, during the process of its biogenesis. It gave away fourteen isotopologues in [1-^13^C] Glc labeling experiment and eleven in [2-^13^C] Glc labeling, providing us with the evidence that isoprene units are derived from MVA route and also the methyl group is of non-isoprenogenic origin (Additional file 1: Figure S4, S5b) [44].

One of the signature fragment of salannin is *m/z* 387.1958, corresponding to its triterpene skeleton, which is without 3 functional groups viz, MeOH, tiglic acid and acetic acid (Additional file 1: Figure S4, S6b). Similiarly, the fragment, *m/z* 419.2214, of salannolacetate gave the same skeleton but with intact methylester moiety (only devoid of isovalerate and acetate group) (Fig. 7b, Additional file 1: Figure S4). These skeletal fragments comprises of 5 complete isoprene units and an incomplete isoprene unit (with only one carbon contribution from it). These fragments have been originated from 26 isoprenogenic carbons and in addition, 1 non-isoprenogenic carbon at methylester group in case of *m/z* 419.2214. [1,6 -^13^C] Glc labeling showed addition of 15 mass units for these fragments, which might have arrived from 5 isoprenes each with three ^13^C labels, indicating that they may have originated through MVA pathway. Eleven ^13^C incorporations occurring in [2-^13^C] Glc labeling further provides the evidence that isoprene units are formed through MVA as the alternative route will result in only 10 labels due to loss of one during the formation of proto-limonoids (Fig. 7b, Additional file 1: Figure S4and S6b).

**Fig. 8:**
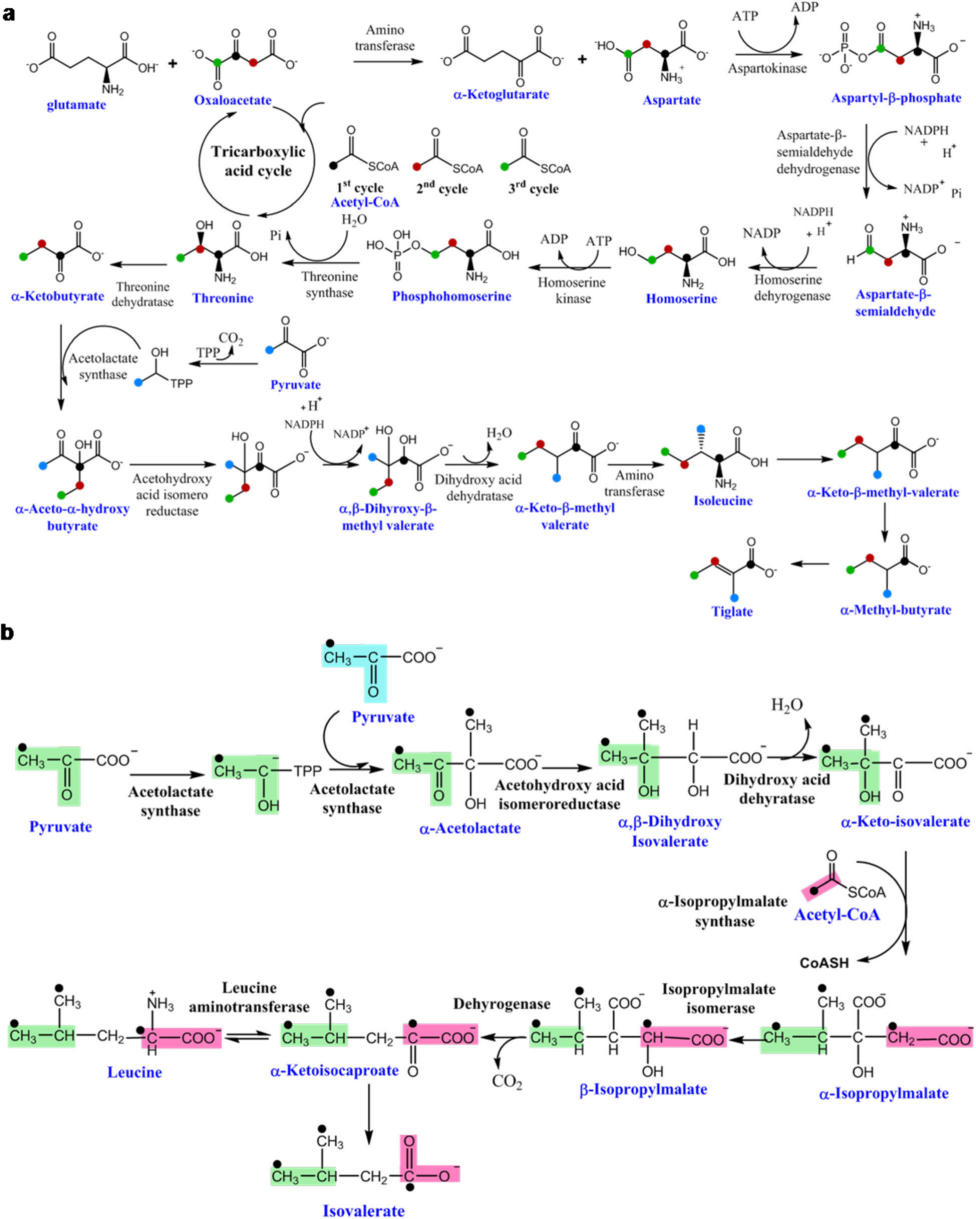
Tracing the flow of ^13^C carbon from [1-^13^C] /[1,6-^13^C] Glc through primary metabolic pathway for the formation of functional groups present in limonoids. (**a**) tiglate group present in azadirachtin and salannin is traced through isoleucine biosynthetic pathways. (**b**) isovalerate group present in salannolacetate is traced through leucine biosynthetic pathway

Further evidence was obtained after studying salannolacetate, for its fragment, *m/z* 245.1538 corresponding to decalin moiety, which is devoid of CH_2_O group at methylester moiety and other functional groups. This fragment is built from 3 complete isoprene units and 1 carbon of fourth isoprene. [1,6-^13^C] and [2-^13^C] Glc labeling study arrived, with addition of 9 and 7 mass units for this fragment respectively, (Fig. 7b), thus corroborating its formation through MVA route. The same pattern of increase in mass units was obtained for another decalin fragment *m/z* 273.1414 (with intact methylester moiety) corresponding to salannin and salannolacetete (Additional file 1: Figure S4). MS/MS traces of unlabeled salannolacetate generated daughter ion, *m/z* 85.0653 corresponding to isovalerate moiety and the ones of labeled salannolacetate showed increase of 3 mass units (*m/z* 86.0681, 87.0723, 88.0753) (Fig. 7b). Isovalerate biosynthesis was traced through primary metabolism leading to amino acid biosynthesis as it diverges from leucine biosynthesis, which corroborates with the formation of 3 mass units in our labeling experiment. Isovalerate was traced through glycolysis and amino acid leucine biosynthetic pathway, which is in agreement with previous study in *Propionibacterium* [45]. Two molecules of [3-^13^C] pyruvate and one [2-^13^C] acetyl-CoA formed from the glycolysis of [1,6-^13^C] Glc contribute for the biosynthesis of leucine (Fig. 8b). Isovalerate originates from ketoisocaproate, a penultimate metabolite in leucine biosynthetic pathway thereby acquiring two ^13^C from labeled pyruvate and one from acetyl-CoA. Our tandem MS data for the isotopologues of salannolacetate supports the presence of three ^13^C labels in isovalerate as evidenced from 3 additional units generated from the fragmentation of salannolacetate isotopologues (Fig. 7b).

When we examined the fragmentation pattern of azadirachtin A obtained from the unlabeled biomass, *m/z* 269.0808 was corresponding to the decalin moiety. Three isoprene units and a single carbon from fourth isoprene contribute to this moiety. ^13^C enrichment through [1-^13^C] Glc labeling showed additional 9 mass units to this fragment suggesting that each of the three ^13^C labels may have originated from a single isoprene unit. The same experiment with [2-^13^C] Glc labeling gave 7 additional mass units further corroborating its route of origin (Fig. 6b). The fragment *m/z* 507.1650 of azadirachtin A was formed devoid of all functional groups except the methylester moieties and this fragment encompasses 5 isoprenes and single carbon of terminal isoprene. This fragment showed additional 15 mass units upon [1-^13^C] Glc labeling and generated 11 mass units upon [2-^13^C] Glc labeling (Fig. 6b). The fragment *m/z* 161.0597 formed from the C, D rings and furan moiety of azadirachtin A, is originated from three isoprene units. This fragment displayed six and four additional mass units corresponding to the [1-^13^C] Glc and [2-^13^C] Glc labeling experiment respectively, indicating that it is biosynthesized by utilizing IPP and DMAPP formed through MVA pathway (Fig. 6b, Additional file 1: Figure S4).

The daughter ion fragment, *m/z* 83.0498, was identified as tiglate group from azadirachtin A, salannin and 3-deacetylsalannin. Four additional mass units (*m/z* 84.0531, 85.0565, 86.0597, 87.0718) were generated after fragmentation of labeled azadirachtin A from [1-^13^C] /[1,6-^13^C] Glc labeling whereas two mass units increase were observed through [2-^13^C] Glc labeling experiment. The earlier report supports the formation of tiglic acid through isoleucine biosynthetic pathway so its origami was traced through multiple primary metabolic pathways (Fig. 8). A plausible scheme (Fig. 8a, b) is presented to determine the carbon position specific biosynthetic origin of tiglate moiety present in azadirachtin A. The [2-^13^C] acetyl-CoA entering the TCA cycle derives its ^13^C label from the C-1 and C-6 positions of [1,6-^13^C] Glc thereby labeling oxaloacetate, the TCA intermediate in subsequent cycles. The resulting oxaloacetate gets ^13^C label at three carbons producing aspartate labeled at C-2, C-3 and C-4 positions which in turn is utilized in threonine biosynthetic pathway, labeling the same positions of carbon in threonine. The amino acid, threonine by addition of another ^13^C label from [3-^13^C] pyruvate followed by isomerization, reduction, dehydration and group transfer reactions forms isoleucine. Isoleucine is converted in two-step reactions to tiglate containing four ^13^C labels. This is corroborated from the four additional mass units generated from MS/MS fragmentation of isotopologues of azadirachtin and salannin (Figs. 6b, 8).

The elaborated pattern of ^13^C distribution in the limonoid skeleton and its functional group obtained through tandem MS analysis of the ex vivo biosynthesized metabolite gives us the evidence that MVA pathway contributes for the biosynthesis of limonoids and the functional groups such as tiglate and isovalerate are formed through isoleucine and leucine amino acid biosynthetic pathways (Figs. 6, 7, 8, Additional file 1: Figure S3, S4, S5, S6).

### Evaluation of contribution from MVA and MEP pathway for limonoid biosynthesis

**Fig. 9:**
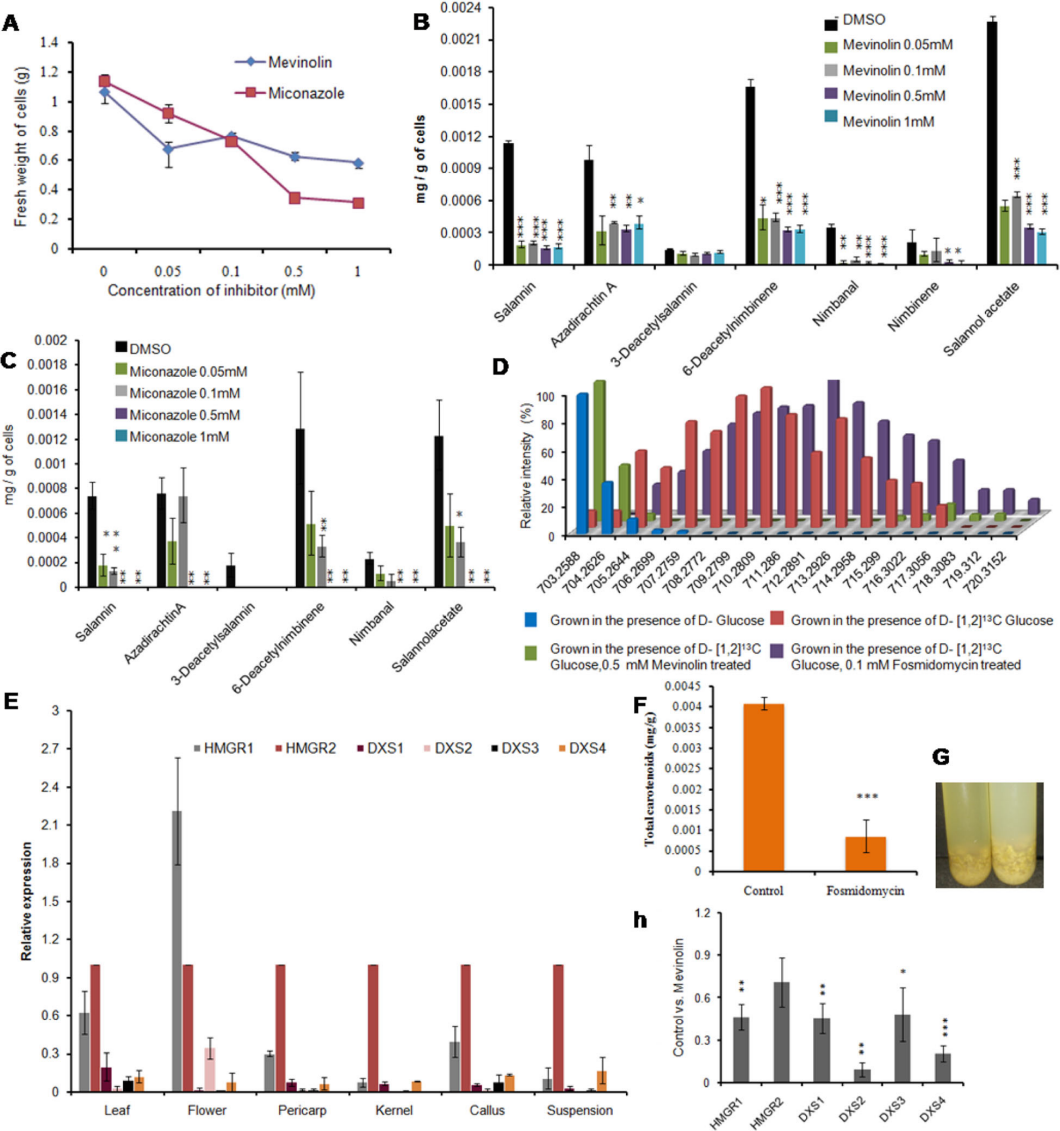
Expression profile and chemical inhibition of MVA and MEP pathway. (**a**) Effect of inhibitors, mevinolin and miconazole on growth of cells at different concentrations. (**b**) Effect of different concentrations of mevinolin on limonoid biosynthesis. (**c**)To study the involvement of cytochrome P450 enzymes in limonoid biosynthesis; miconazole an inhibitor of cytochrome enzymeswas used at different concentrations. (**d**) Chemically inhibited MVA and MEP pathway cells grown in the presence of [1,2-^13^C] Glc to monitor the formation of isotopologues. (**e**) Expression profile of HMGR and DXS genes in various tissues relative to the expression of HMGR2. (**f**) Effect of 0.1 mM fosmidomycin on MEP pathway metabolite, carotenoids. (**g**) control vs fosmidomycin treatment demonstrating the blanching of color of cell suspension due to drop in carotenoid level. (**h**) Comparison of expression profile of the studied genes in mevinolin treated cells with respect to the control

In order to establish further, the role of MVA and MEP pathways in limonoid biosynthesis, ^13^C-Glc labeling studies were carried out in presence of pathway specific chemical inhibitor. When suspension cultures treated with MVA pathway specific inhibitor, mevinolin, affected both the growth and limonoid content simultaneously (Fig. 9a, b). When 0.1 mM fosmidomycin was used to inhibit MEP pathway from the formation of isoprene units, the levels of total carotenoid content was decreased by 78.85% as compared to that of control (Fig. 9f). Further the suspension culture showed blanching during the course of exponential growth of the cells in presence of fosmidomycin (Fig.9g). Furthermore, the inhibition studies were carried out using both inhibitors individually in presence of Glc and [1,2-^13^C] Glc. Fosmidomycin treatment didn’t interfere with the biosynthesis of limonoids as evidenced from the formation of ^13^C limonoid isotopologues whereas in case of inhibition of MVA pathway with mevinolin, drastic decrease in the levels of ^13^C isotopologues was observed (Fig. 9d).

Miconazole, the inhibitor of cytochrome enzymes affects the growth of cells relatively higher to that of mevinolin at higher doses, 0.5 and 1 mM concentration (Fig. 9a). The limonoid biosynthesis is also severely affected with miconazole from 0.1 mM concentration and above. The concentration of limonoids biosynthesized is comparatively less in miconazole treated cells to that of the one with mevinolin (Fig. 9b, c).

### Expression profiling of genes involved in rate limiting step of MVA and MEP pathway

The spatial expression of the genes encoding the rate limiting enzymes involved in MVA and MEP pathway were studied in different neem tissues such as pericarp and kernel of fruit, leaf, flower, suspension and callus derived from kernel so as to correlate their transcript level to the limonoid biosynthesis. Two transcripts for HMGR and 4 for DXS from MVA and MEP pathway respectively were identified from the neem transcriptome [3] and the real time analysis for all the 6 sequences were performed to determine their expression profile. Very little known regarding the expression level of HMGR1 and HMGR2, here we are reporting the relative expression levels of HMGR1, HMGR2 and four DXS genes among various neem tissues (Fig. 9e). Even though all the isoforms of HMGR and DXS are expressed, the relative expression of HMGR2 was predominant in all the tissues studied, with the exception of flower (Fig. 9e). In kernel and pericarp, the expression of DXS transcripts was very low when compared to the HMGR levels that substantiate the results of ^13^C labeling and inhibitor studies that MVA pathway plays a major role in limonoid biosynthesis. We also compared the transcript abundance of each gene in different tissues studied (Additional file 1: Figure S7). Among the DXS isoforms, DXS1 and DXS3, showed higher level of expression in the leaf tissue whereas, DXS4 level was slightly higher in suspension culture when compared to the leaf tissue. DXS2 and HMGR1 showed comparatively very high level of expression in flower among the tissues studied. The level of expression of HMGR2 was high in kernel and suspension cells relative to the other tissues (Additional file 1: Figure S7). Further, to study the effect of pharmacological block of MVA pathway by inhibitor, mevinolin on the transcript level, the real time PCR profiling was carried out with mevinolin treated neem suspension cells. In comparison to control, the expression profile showed that the relative expression levels of all the seven studied genes of inhibitor treated cells were comparatively reduced (Fig. 9h).

## Discussion

Studies have been carried out to find the azadirachtin production from cell culture developed from different plant parts and thereby aiming to maximize its productivity [6, 7]. Our study gives insights into specific limonoid profile during growth and development of neem tree. The neem cotyledons inoculated over media has grown into seedling, the withered cotyledon has lost its limonoid content and limonoids might have been distributed into the growing plant part during its development. This has been hypothesized based on the quantitative comparison of individual limonoid content of the different plant parts and found that azadirachtin A was highest in stem, followed by root and leaf during plant development. Limonoid profiling studies of in vitro plant and neem sapling leaves demonstrated the high level occurrence of C-seco limonoids, specifically azadirachtin A in similitude to the pattern of kernel tissue (Fig. 2) whereas the leaves of wild grown neem tree were known to contain ring intact limonoid, nimocinolas a major compound [3].This growth specific differential limonoid profile can be explained in terms of chemical defense theory [46]. The high levels of potent insect deterrent azadirachtin A can protect the young leaf, as soft tissues are more susceptible to herbivory. Whereas azadirachtin A content is less (700 fold less than nimocinol) in mature leaf. Similar observation on defense mechanism has been reported in tea leaves, where caffeine is found to accumulate in young leaves and its biosynthesis has been halted as the leaf ages and get cellulose and lignin deposits for its protection from predators [47].

Our present study indicates that undifferentiated, suspension cells and callus derived from kernel contains C-seco limonoids. C-seco limonoids are continued to be biosynthesized in subsequent subculturing (Fig. 3), and on the other hand shows the absence of ring intact limonoids. This follows the trend of limonoid content as that of its parent tissue, the fruit kernel however the occurrence of individual C-seco limonoids varies. Azadirachtin A was found to be the abundant C-seco limonoids in kernel whereas kernel derived cell lines contain 6-deacetylnimbin and 6-deacetylnimbinene in high levels (Fig. 3). Attenuation of limonoid biosynthesis in cell lines of the suspension derived from callus maintained more than a year was clearly evident from the staggered limonoid isotopologues generated (Fig. 4).

As signified from literature [32, 33, 48], labeling studies with stable isotopes have greatly helped in studying the various biosynthetic pathways in living systems. The present study has been carried out with isotopomers and isotopologues of glucose, (1-^13^C, 2-^13^C and 1,6-^13^C glucose) in order to trace the limonoid biosynthetic pathway and with 1,2-^13^C glucose to track limonoid formation during the blockage of either of the isoprene biosynthetic pathway. In *Croton sublyratus* and *Withania somnifera*, sterols and triterpenes are biosynthesized through both the pathways [34, 49] but the study of ^13^C labeling of limonoids in the neem cell suspension strongly supports the fact that MVA pathway contributes for the isoprene units of limonoids. The cross-talk between both pathways (MVA and MEP) has been observed in normal condition or during environmental stress for the biosynthesis of different metabolites in plant system [37–41], was not observed with the cell lines of neem. Tiglate and isovalerate group constitutes an important functional group of C-seco limonoids. Tiglate and isovalerate biosynthesis has been traced through glycolysis, TCA cycle and amino acid biosynthetic pathways with ^13^C label originated from C-1 and C-6 positions of glucose through amino acid isoleucine and leucine respectively, and is in consistent with earlier reports [50–53] (Fig. 8).

**Fig. 10:**
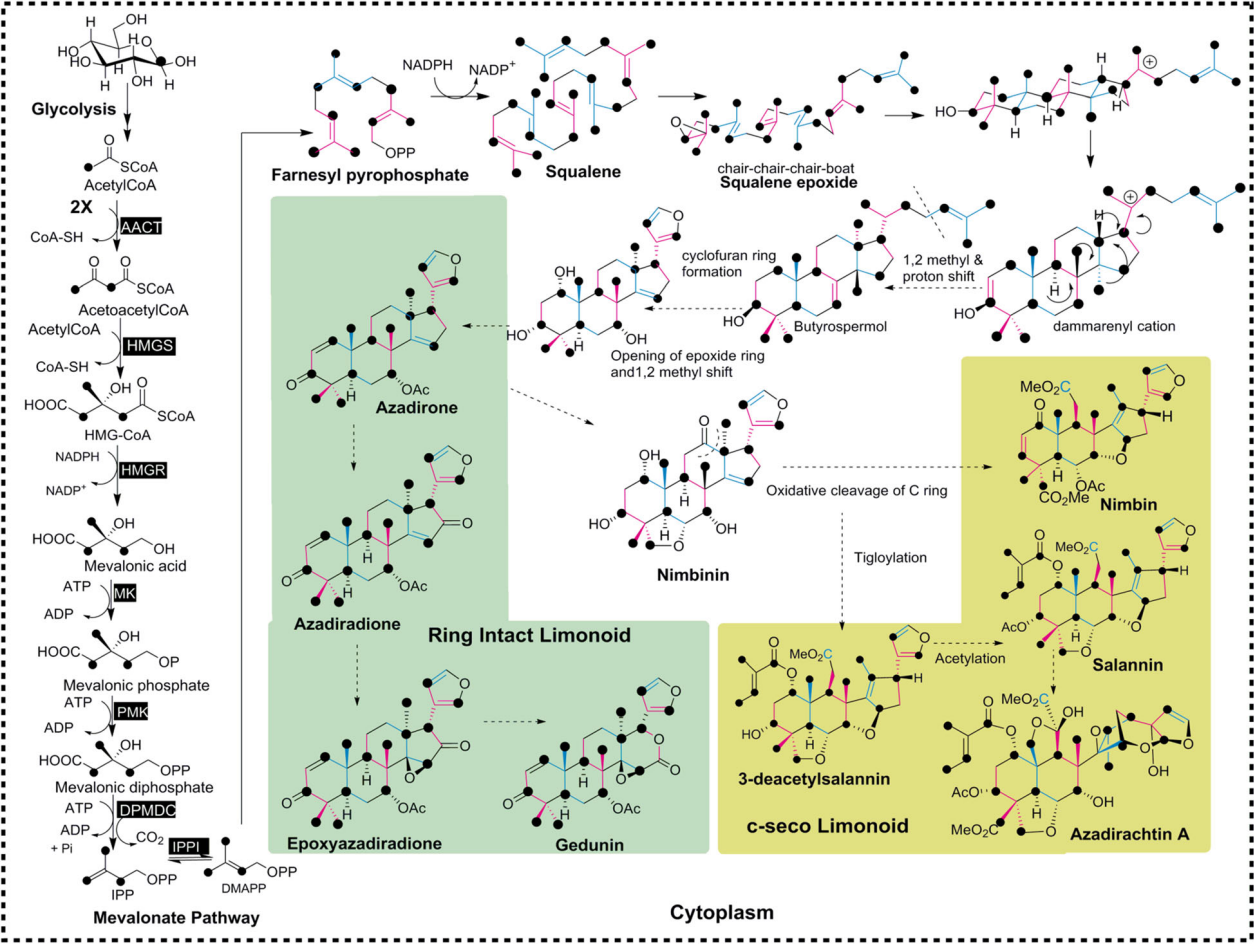
Simplified scheme showing limonoid biosynthesis in neem tree being contributed by isoprene units formed through MVA pathways evident from the labeling experiment with ^13^C glucose. C-seco and ring intact limonoid are represented in yellow and green boxes respectively. (Alternate isoprene units of the limonoid skeleton are shown in pink and blue, dotted arrow shown here are the uncharacterized steps in the pathway)

Thus, ex vivo labeling studies carried out with ^13^C labeled glucose gives proof of evidence that MVA pathway contributes for the isoprene units of limonoid skeleton and further, inhibitor treatment combined with [1,2-^13^C] Glc labeling experiment shows that blocking the MEP pathway doesn’t have any impact on the limonoid biosynthesis. The blanching of cells due to drop in carotenoids during fosmidomycin inhibition was consistent with the leaf bleaching observed in *Arabidopsis* seedling [39]. Spatial expression profile of genes encoding for the rate-limiting step of isoprene biosynthetic pathway shows that HMGR relative expression level being the highest when compared to MEP pathway genes in all the tissues and cells studied, further bolstering the finding that mevalonate pathway contributes for the isoprene units of limonoid skeleton (Fig. 9e). Though blocking of MVA pathway at 0.05 mM mevinolin concentration does not affect the growth significantly, the metabolic process of cell and limonoid biosynthesis were decreased, which is also evident from the marginal reduction in transcript level of HMGR and DXS genes, the rate-limiting steps of both the pathways. Miconazole, the inhibitor of cytochrome enzymes [48], severely affected limonoid biosynthesis in cell suspension culture when compared to Mevinolin (Fig. 9), which signifies the fact that cytochrome P450 enzymes play a major role in the limonoid biosynthetic pathway, as most of the downstream steps in the pathway are subsequent oxidation and reduction reactions (Fig. 10).

## Conclusion

Study of limonoid biosynthesis in neem tree is of potential significance as it produces agriculturally and pharmacologically important molecules.This study provides insights into the limonoid synthesizing ability of in vitro cultures cell lines and neem tree. The work emphasizes the crucial role of MVA pathway and found to be the sole source of isoprene units for limonoid biosynthesis. Further, no identified cross-talk between both MEP and MVA isoprene biosynthetic pathways was observed. Amino acid leucine and isoleucine biosynthetic pathways contributes to the building blocks for the functional groups of limonoid. Based on High Resolution Mass Spectrometry based tandem MS of labeled limonoids obtained from feeding studies, the biosynthetic pathway has been traced for these complex molecules.

## Methods

### Plant materials

One of the healthy trees in CSIR-NCL campus was used for sample collection for all the analysis reported in our work. Neem leaves, flowers, and fruits at different developmental stages were collected from the same tree during the month of April–June as per laboratory guidelines, they were flash frozen and stored at − 80 °C until required for the experiments. For the initiation of cell culture, fruits in third to fourth developmental stages were collected freshly.

### Growth conditions for neem plantlets and cell cultures

Hard green neem (*Azadirachta indica)* fruits were surface sterilized and the cotyledons (kernel) was obtained from the fruit. The cotyledon was inoculated over MS (Murashige and Skoog) basal media with 0.25% Phytagel™ (Sigma) and incubated at 25 °C in a growth chamber (Percival Scientific) with a cycle of 16-h light/8-h darkness.

For formation of callus, the kernel was inoculated over MS basal media supplemented with 3% sucrose, 2 mg/l NAA and 0.2 mg/l BA at pH 5.8 and solidified with 0.25% Phytagel™ under the conditions of 600 lm of light in 16-h light/8-h darkness cycle at 25 °C*. Azadirachta indica* suspension cell cultures were initiated from cultured callus and maintained in liquid MS (Murashige and Skoog) basal media of same composition mentioned earlier and grown with rotatory gyration at 125 rpm at 25 °C in dark.

### Harvesting cells and extraction of limonoids

The suspension culture cells were collected from media by centrifugation at 2500 × *g* for 10 min at 25 °C. The cells were homogenized in ice-cold methanol. The supernatant was collected after centrifugation at 2500 × *g* for 10 min at 25 °C. The cells were again resuspended in twice the volume of methanol and extracted twice again. The methanol extract was pooled together and evaporated to dryness. The residue was partitioned between water and ethylacetate thrice. The ethylacetate pool was then evaporated to dryness under reduced pressure and constituted with LC-MS grade methanol to the volume of 1 ml. To quantify limonoids in the media, it was partitioned with ethylacetate and extracted, as mentioned above.

### Limonoid profiling in callus and cell culture

Targeted metabolic profiling of callus at different subculturing stage of callus and cell suspension was carried out with LC-ESI-MS as described below by comparison with the 19 limonoid standards isolated and characterized from neem fruit and oil [43, 54]. The identified limonoids were subjected to tandem MS to confirm their identity based on the presence or absence of the signature fragments as established in our previous study [43, 54].

### LC-ESI-mass spectrometry conditions

Analysis of neem limonoids was performed with Thermo Scientific QExactive™ hybrid quadrupole-Orbitrap mass spectrometer associated with Accela 1250 pump and Accela open AS. The conditions of HESI source include capillary temperature of 320 °C, heater temperature at 350 °C, s-lens RF level of 50, Spray voltage of 3.6 kV, spray current of 0.9 μA with sheath gas flow rate of 41, Auxiliary gas flow rate of 9 and sweep gas flow rate of 3. Standards as well as the extracted samples were analyzed in positive ionization mode, in full MS-scan with scan range of 100 to 1000 *m/z*. Following were the properties of the scan performed- resolution 70,000, AGC target 1e6, Maximum IT 200 ms. Waters Acquity UPLC BEH C_18_ column (particle size 1.7 μm, 2.1 X 100 mm) was used as the stationary phase while the solvent system of methanol and water containing 0.1% formic acid served as the mobile phase. The gradient started with 40% methanol (5 min isocratic), it was then increased to 50% (5 min isocratic), followed by 60% methanol for the next 15 min and over the next 4 min it was isocratic with 65% methanol. It was then increased to 90% methanol for 4 min. For the last 2 min, it was isocratic with 40% methanol. Constant flow rate of 0.3 ml min^− 1^ was maintained throughout the run time of 35 min. The chromatograms and mass spectral data were processed by Xcaliburqual browser (version 2.3; Thermo Scientific).

### Time course of cellular growth and Limonoid formation

0.2 g of callus tissue was inoculated in liquid MS to generate suspension and the cells were harvested at 12 different time points. Fresh weight of cells at different time points were estimated and limonoids were extracted from cell mass at each time point. Based on the calibration curve of authentic standards, 9 limonoids were quantified by considering the peak area of extracted ion chromatogram (EIC) corresponding to the combined protonated and sodiated molecular ions [3]. From the in vitro plantlets, leaf, stem, root and withered cotyledon were collected and quantification of limonoids was performed as mentioned above.

### Feeding experiment with ^13^C Glc tracers

For feeding experiments, cells from 2 days old suspension was harvested from media and sub-cultured into media with labeled carbon source. Feeding experiments with D-[1-^13^C] Glc, D-[2-^13^C] Glc and D-[1,6-^13^C] Glc (99% enriched, Cambridge Isotope Lab. Inc., Andover, MA) were performed separately in 25 ml Erlenmeyer flasks containing 5 ml MS liquid with 3% of labeled Glucose in duplicates.

### Establishment of structure-fragment relationship for azadirachtin A

To establish the structure-fragment relationship for azadirachtin A, derivatives of azadirachtin such as azadirachtin B, 11-epi-azadirachtin D, azadirachtin H, 3-deacetylazadirachtin A, vepaol isolated and purified from neem seeds were subjected to MS/MS at various NCEs such as 10%, 15%, 20% and 25%. Based on the comparison of structure of different molecules of azadirachtin and their respective fragmentation pattern comprising of fragments from higher to lower *m/z* values, the structure of each fragment obtained after fragmentation of azadirachtin A molecule was established.

### Tandem mass spectrometry of limonoids and its isotopologues

Tandem Mass spectrometry of limonoids and its isotopologues was done in data dependent acquisition mode (PRM) with the protonated molecular ion as a precursor for MS/MS. Isolation window was set to 1, the charge state of 1, NCE 20% and at their corresponding retention time for all the nine limonoids and its isotopologues obtained from cell suspension were set for tandem mass spectrometry. The MS/MS distribution for each isotopologue of four limonoids, azadirachtin A, salannin, salannolacetate and 6-deacetylnimbinene were evaluated individually for different ^13^C glucose labeling. Consideration of *m/z* values accurately to fourth decimal for individual isotopologues and isotopomers eliminated the noise generated during MS and MS/MS analysis.

### Inhibition studies

Mevinolin and fosmidomycin (Sigma) were solubilized in DMSO and water respectively, filter sterilized using membrane filter of 0.22 μm pore size (Millipore) and introduced into culture under aseptic conditions. The inhibitor of cytochrome P450 system, miconazole was added to the neem suspension cultures at different concentrations along with control for each treatment containing the same solvent in which inhibitors are dissolved. Suspension cultures were treated in triplicates with mevinolin and miconazole at different concentrations such as 0, 0.05, 0.1, 0.5 and 1 mM. Quantification of biomass of cells was done by determining the fresh weight of cells by centrifugation in swinging bucket at 1000 × *g* (Thermofisher Scientific).

Mevinolin (Final concentration 0.05 mM) and fosmidomycin (Final concentration 0.1 mM) were added to the cell suspension culture after 2 days of initiation of culture after replacing the cells with fresh media containing no sugars. 3% D- [1,2-^13^C] Glc (99% enriched, Cambridge Isotope Lab. Inc., Andover, MA) was supplemented to the 5 ml culture after inhibitor treatment. Two independent experiments were performed each in duplicates. Limonoid extraction was carried out from the cells after ten days of fosmidomycin addition. To further analyze the total carotenoid content, the cells were frozen in liquid nitrogen, made into fine powder and extracted with acetone in the dark. The extract was centrifuged to remove debris and concentrated to the volume of 3 mL. It was read spectrometrically at 470 nm and the total carotenoid contents were calculated as described earlier [24].

### Quantitative (real-time) RT PCR studies

For quantitative PCR analysis, RNA isolation was carried out using Spectrum™ plant total RNA isolation kit (Sigma) from neem callus, suspension, leaf, flower, pericarp, kernel tissues and also from the control and 0.05 mM mevinolin treated suspension cells. RNA isolation was followed by DNase treatment by using DNase I Amplification Grade kit (Sigma). cDNA was synthesized using SuperScript® III First-Strand Synthesis System (Invitrogen) from 3 μg of total RNA. Through transcriptome analysis, we could identify two ORFs encoding for HMG-CoA reductase (HMGR) and 4 for deoxyxylulose-phosphate synthase (DXS), the enzyme involved in the catalysis of rate limiting step in MVA and MEP pathway respectively [3]. qPCR primers were designed for the above 6 transcripts along with the housekeeping genes, elongation initiation factor 4a (ELF4A) and actin (adopted from Rajakani et al. [55]) for normalization (Additional file 1: Table S2). Real-time PCR was carried out in AriaMxRealtime PCR system (Agilent Technologies) by using FastStart Universal SYBR Green Master (Roche) with cDNA template diluted 20 times. The cycling conditions comprised of initial denaturation at 95 °C for 5 min followed by 40 amplification cycles consisting of 95 °C for 15 s and 58 °C for 30 s. The real-time amplification data was analyzed using software (Agilent Technologies). All reactions were performedin triplicate assays for 3 biological replicates and relative quantifications were performed using ΔΔC_T_ method. PCR efficiencies were calculated based on the slope of dilution curve of control cDNA of cell suspension. The two housekeeping genes gave same level of expression and the results were normalized with ELF4A.

### Statistical analysis

Asterisks on the tops of bar denotes the values that were determined by Student’s t-test using Microsoft Office Excel 2007. They were found to be significantly different from their respective controls (*, **, ***, indicate *p* < 0.05, 0.01 and 0.001, respectively).

## Additional files

Additional file 1: This file contains MS data of limonoids, MS/MS data for confirmation of limonoids from cell culture, ^13^C isotopologue distribution for limonoids, primer information, real time PCR for rate-limiting genes of MVA and MEP pathway. **Figure S1.** MS/MS confirmation of limonoids identified in cell culture. **Figure S2.** Variation of intensity of daughter ions of azadirachtin A and its structure fragment relationship. **Figure S3.** Comparison of isotopologues of limonoids obtained from feeding experiment (with different ^13^C labeled glucose) with that of the control. **Figure S4.** Comparison of MS/MS of limonoids obtained from feeding experiment (with different ^13^C labeled glucose) with that of the control. **Figure S5.** Relative isotopologue distribution and Heatmap for distribution of isotopologues for specific fragments of 6-deacetylnimbinene. **Figure S6.** Relative isotopologue distribution and Heatmap for distribution of isotopologues for specific fragments of salannin. **Figure S7.** Relative expression level of genes of MVA and MEP pathway in different neem tissues. **Table S1.** MS and MS/MS data for azadirachtin A and its derivatives. **Table S2.** Primers used in real time PCR analysis. (PDF 2627 kb)

## Abbreviations

DXS: Deoxyxylulose-phosphate synthase
Glc: Glucose
HMGR: 3-hydroxy-3-methyl-glutaryl-coenzyme A reductase
HRMS: High Resolution Mass Spectrometry
MEP: Methylerythritol 4-phosphate
MVA: Mevalonate
NCE: Normalized Collision Energy
TCA: Tricarboxylic acid
UPLC: Ultra Pressure Liquid Chromatography
