# Supplementary material for: Tracing the biosynthetic origin of limonoids and their functional groups through stable isotope labeling and inhibition in neem tree (Azadirachta indica) cell suspension
Source: BMC Plant Biol. 2018 Oct 11;18:230. doi: 10.1186/s12870-018-1447-6 (PMC6186041; doi:10.1186/s12870-018-1447-6)
Supplement: Supplementary file 1 — This file contains MS data of limonoids, MS/MS data for confirmation of limonoids from cell culture, 13C isotopologue distribution for limonoids, primer information, real time PCR for rate-limiting genes of MVA and MEP pathway. Figure S1. MS/MS confirmation of limonoids identified in cell culture. Figure S2. Variation of intensity of daughter ions of azadirachtin A and its structure fragment relationship. Figure S3. Comparison of isotopologues of limonoids obtained from feeding experiment (with different 13C labeled glucose) with that of the control. Figure S4. Comparison of MS/MS of limonoids obtained from feeding experiment (with different 13C labeled glucose) with that of the control. Figure S5. Relative isotopologue distribution and Heatmap for distribution of isotopologues for specific fragments of 6-deacetylnimbinene. Figure S6. Relative isotopologue distribution and Heatmap for distribution of isotopologues for specific fragments of salannin. Figure S7. Relative expression level of genes of MVA and MEP pathway in different neem tissues. Table S1. MS and MS/MS data for azadirachtin A and its derivatives. Table S2. Primers used in real time PCR analysis. (PDF 2627 kb) [file 12870_2018_1447_MOESM1_ESM.pdf]

Tracing the biosynthetic origin of limonoids and their functional groups through stable isotope labeling and pathway inhibition in *Azadirachta indica* cell suspension

Thiagarayaselvam Aarthy, Fayaj A. Mulani, Avinash Pandreka, Ashish Kumar, Sharvani S. Nandikol, Saikat Haldar, Hirekodathakallu V. Thulasiram

Additional file

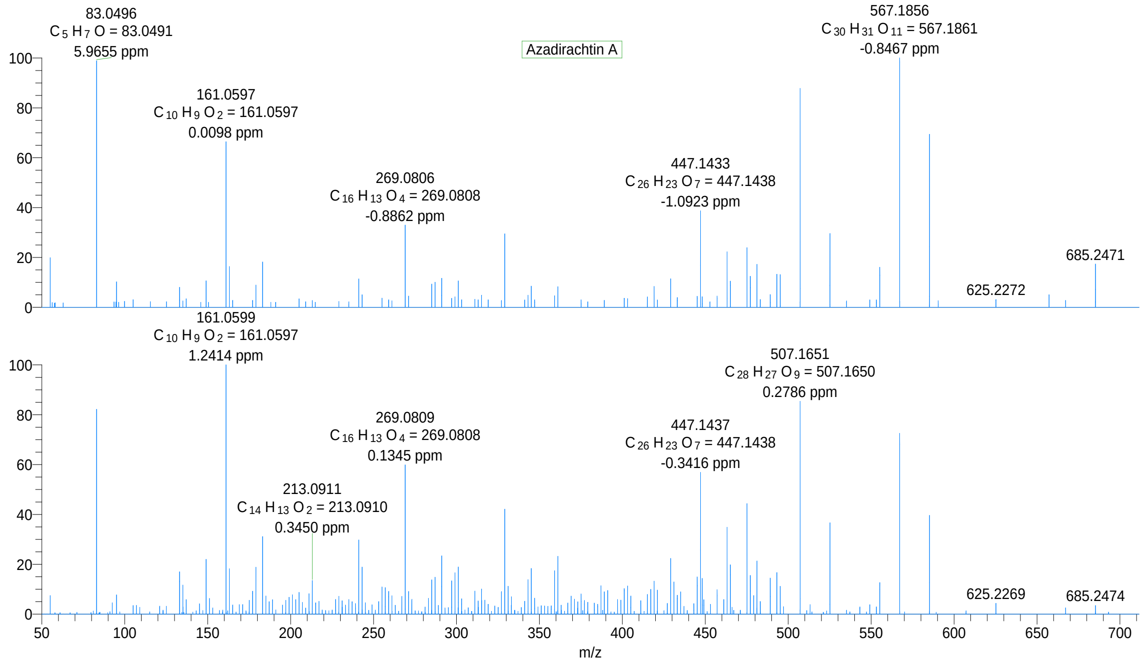

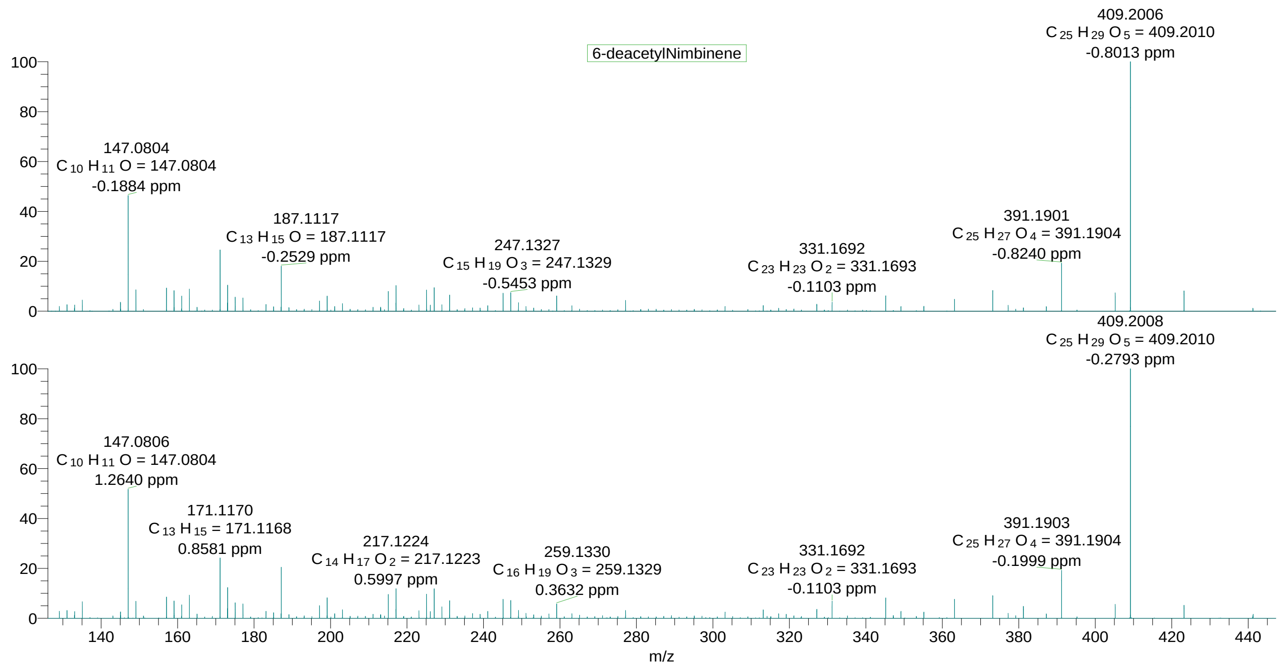
6-deacetylNimbinene
409.2006
C 25 H 29 O 5 = 409.2010
-0.8013 ppm
147.0804
C 10 H 11 O = 147.0804
-0.1884 ppm
187.1117
C 13 H 15 O = 187.1117
-0.2529 ppm
247.1327
C 15 H 19 O 3 = 247.1329
-0.5453 ppm
331.1692
C 23 H 23 O 2 = 331.1693
-0.1103 ppm
391.1901
C 25 H 27 O 4 = 391.1904
-0.8240 ppm
409.2008
C 25 H 29 O 5 = 409.2010
-0.2793 ppm
147.0806
C 10 H 11 O = 147.0804
1.2640 ppm
171.1170
C 13 H 15 = 171.1168
0.8581 ppm
217.1224
C 14 H 17 O 2 = 217.1223
0.5997 ppm
259.1330
C 16 H 19 O 3 = 259.1329
0.3632 ppm
331.1692
C 23 H 23 O 2 = 331.1693
-0.1103 ppm
391.1903
C 25 H 27 O 4 = 391.1904
-0.1999 ppm
m/z

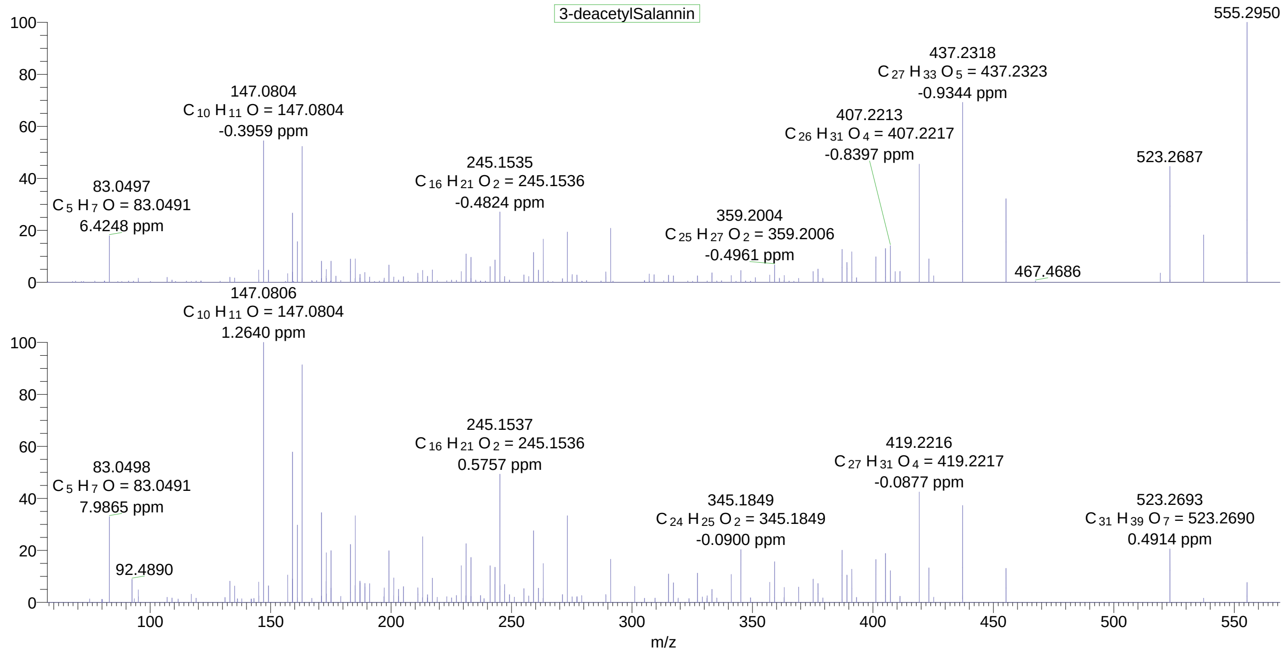
3-deacetylSalannin
555.2950
437.2318
C 27 H 33 O 5 = 437.2323
-0.9344 ppm
407.2213
C 26 H 31 O 4 = 407.2217
-0.8397 ppm
147.0804
C 10 H 11 O = 147.0804
-0.3959 ppm
245.1535
C 16 H 21 O 2 = 245.1536
-0.4824 ppm
523.2687
83.0497
C 5 H 7 O = 83.0491
6.4248 ppm
359.2004
C 25 H 27 O 2 = 359.2006
-0.4961 ppm
467.4686
147.0806
C 10 H 11 O = 147.0804
1.2640 ppm
245.1537
C 16 H 21 O 2 = 245.1536
0.5757 ppm
83.0498
C 5 H 7 O = 83.0491
7.9865 ppm
419.2216
C 27 H 31 O 4 = 419.2217
-0.0877 ppm
345.1849
C 24 H 25 O 2 = 345.1849
-0.0900 ppm
523.2693
C 31 H 39 O 7 = 523.2690
0.4914 ppm
92.4890
m/z

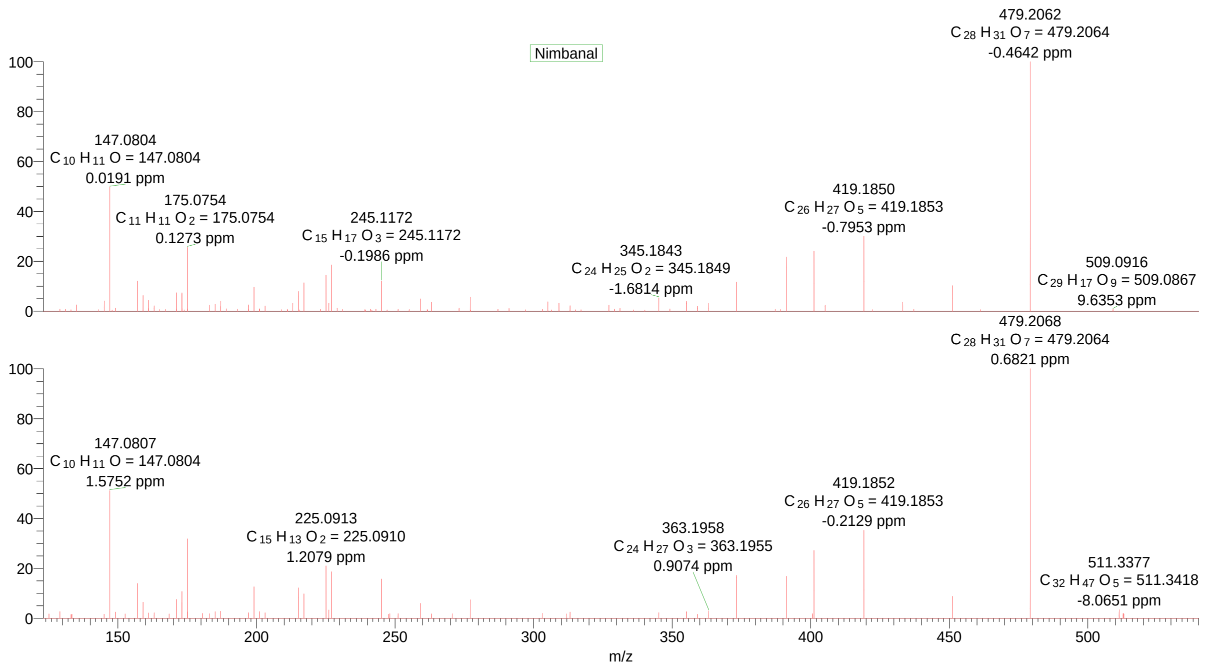
Nimbanal
479.2062
$C_{28}H_{31}O_7$ = 479.2064
-0.4642 ppm
147.0804
$C_{10}H_{11}O$ = 147.0804
0.0191 ppm
175.0754
$C_{11}H_{11}O_2$ = 175.0754
0.1273 ppm
245.1172
$C_{15}H_{17}O_3$ = 245.1172
-0.1986 ppm
345.1843
$C_{24}H_{25}O_2$ = 345.1849
-1.6814 ppm
419.1850
$C_{26}H_{27}O_5$ = 419.1853
-0.7953 ppm
509.0916
$C_{29}H_{17}O_9$ = 509.0867
9.6353 ppm
479.2068
$C_{28}H_{31}O_7$ = 479.2064
0.6821 ppm
147.0807
$C_{10}H_{11}O$ = 147.0804
1.5752 ppm
225.0913
$C_{15}H_{13}O_2$ = 225.0910
1.2079 ppm
363.1958
$C_{24}H_{27}O_3$ = 363.1955
0.9074 ppm
419.1852
$C_{26}H_{27}O_5$ = 419.1853
-0.2129 ppm
511.3377
$C_{32}H_{47}O_5$ = 511.3418
-8.0651 ppm
m/z

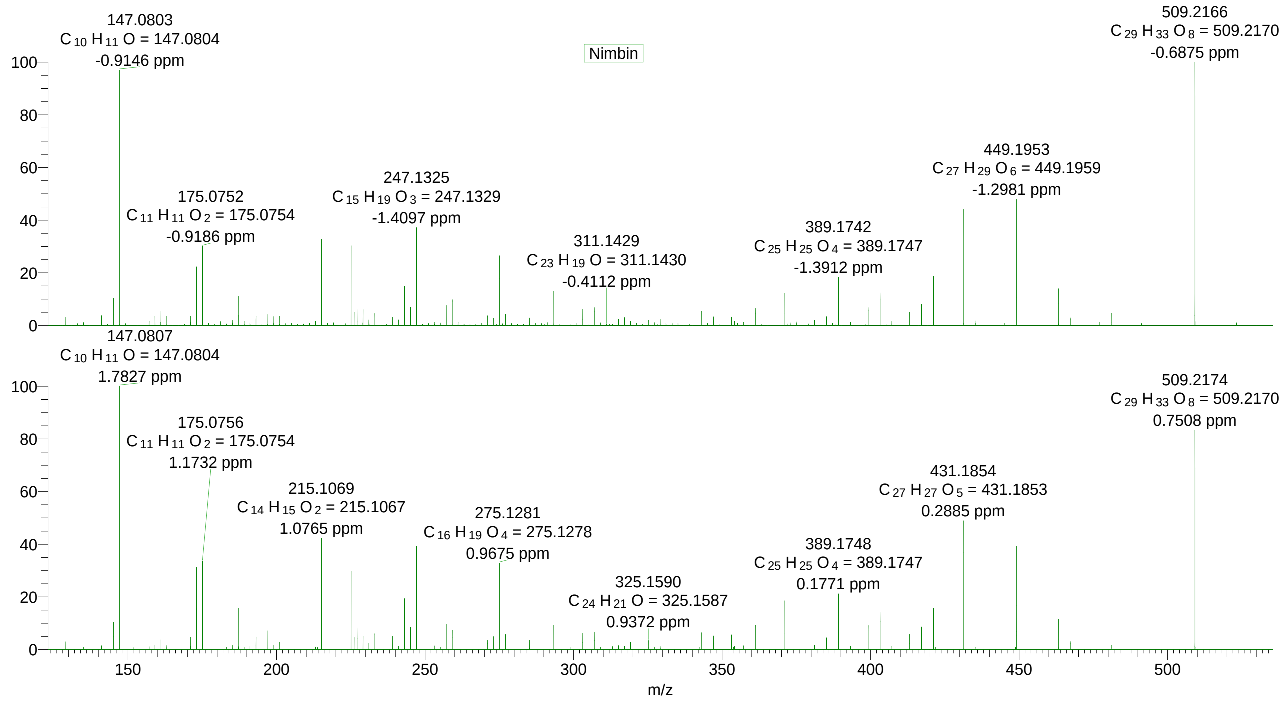
Nimbin
147.0803
$C_{10}H_{11}O$ = 147.0804
-0.9146 ppm
509.2166
$C_{29}H_{33}O_8$ = 509.2170
-0.6875 ppm
175.0752
$C_{11}H_{11}O_2$ = 175.0754
-0.9186 ppm
247.1325
$C_{15}H_{19}O_3$ = 247.1329
-1.4097 ppm
311.1429
$C_{23}H_{19}O$ = 311.1430
-0.4112 ppm
389.1742
$C_{25}H_{25}O_4$ = 389.1747
-1.3912 ppm
449.1953
$C_{27}H_{29}O_6$ = 449.1959
-1.2981 ppm
147.0807
$C_{10}H_{11}O$ = 147.0804
1.7827 ppm
175.0756
$C_{11}H_{11}O_2$ = 175.0754
1.1732 ppm
215.1069
$C_{14}H_{15}O_2$ = 215.1067
1.0765 ppm
275.1281
$C_{16}H_{19}O_4$ = 275.1278
0.9675 ppm
325.1590
$C_{24}H_{21}O$ = 325.1587
0.9372 ppm
389.1748
$C_{25}H_{25}O_4$ = 389.1747
0.1771 ppm
431.1854
$C_{27}H_{27}O_5$ = 431.1853
0.2885 ppm
509.2174
$C_{29}H_{33}O_8$ = 509.2170
0.7508 ppm
m/z

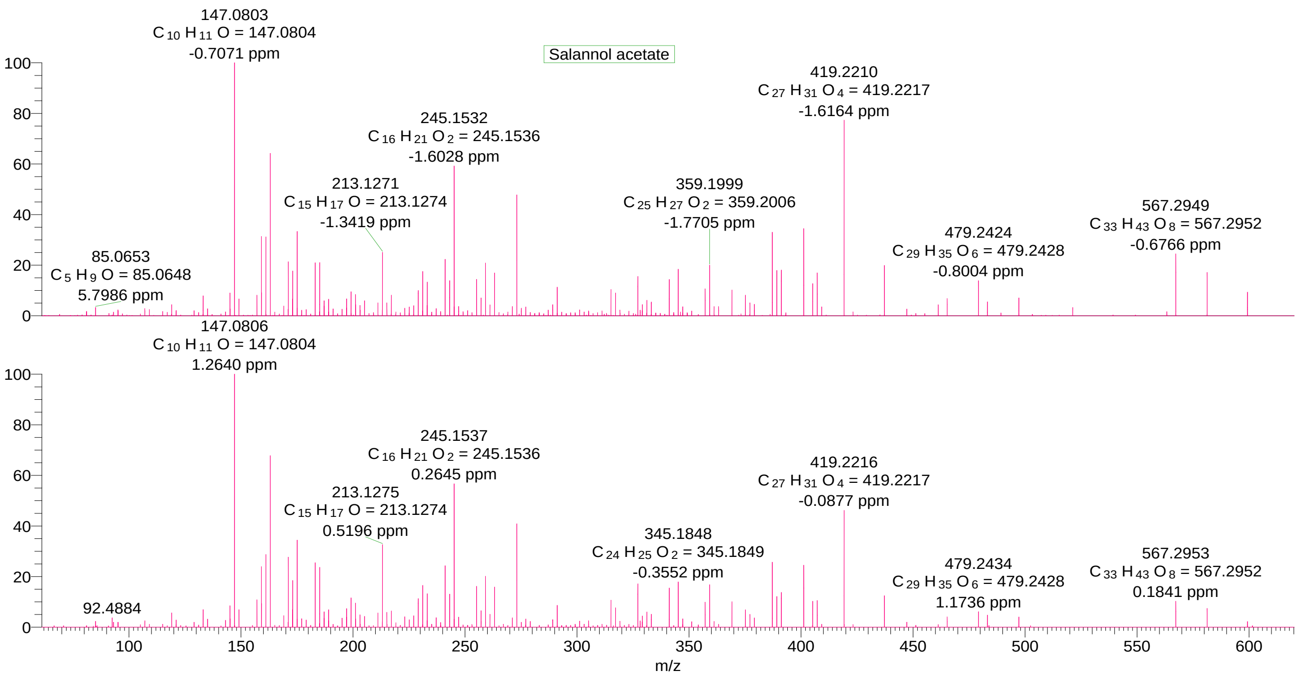
Salannol acetate
147.0803
C 10 H 11 O = 147.0804
-0.7071 ppm
245.1532
C 16 H 21 O 2 = 245.1536
-1.6028 ppm
213.1271
C 15 H 17 O = 213.1274
-1.3419 ppm
85.0653
C 5 H 9 O = 85.0648
5.7986 ppm
359.1999
C 25 H 27 O 2 = 359.2006
-1.7705 ppm
419.2210
C 27 H 31 O 4 = 419.2217
-1.6164 ppm
479.2424
C 29 H 35 O 6 = 479.2428
-0.8004 ppm
567.2949
C 33 H 43 O 8 = 567.2952
-0.6766 ppm
147.0806
C 10 H 11 O = 147.0804
1.2640 ppm
245.1537
C 16 H 21 O 2 = 245.1536
0.2645 ppm
213.1275
C 15 H 17 O = 213.1274
0.5196 ppm
92.4884
345.1848
C 24 H 25 O 2 = 345.1849
-0.3552 ppm
419.2216
C 27 H 31 O 4 = 419.2217
-0.0877 ppm
479.2434
C 29 H 35 O 6 = 479.2428
1.1736 ppm
567.2953
C 33 H 43 O 8 = 567.2952
0.1841 ppm
0
20
40
60
80
100
100
150
200
250
300
350
400
450
500
550
600
m/z

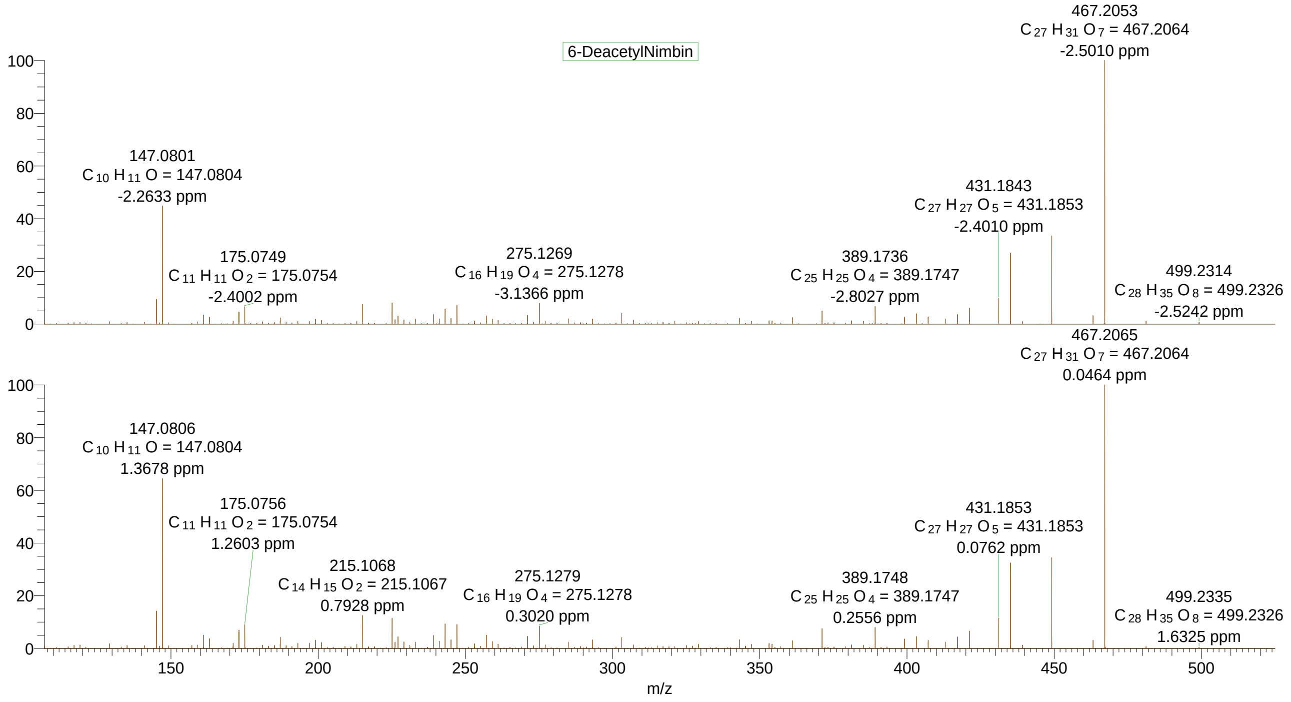
6-DeacetylNimbin
467.2053
C 27 H 31 O 7 = 467.2064
-2.5010 ppm
147.0801
C 10 H 11 O = 147.0804
-2.2633 ppm
175.0749
C 11 H 11 O 2 = 175.0754
-2.4002 ppm
275.1269
C 16 H 19 O 4 = 275.1278
-3.1366 ppm
389.1736
C 25 H 25 O 4 = 389.1747
-2.8027 ppm
431.1843
C 27 H 27 O 5 = 431.1853
-2.4010 ppm
499.2314
C 28 H 35 O 8 = 499.2326
-2.5242 ppm
467.2065
C 27 H 31 O 7 = 467.2064
0.0464 ppm
147.0806
C 10 H 11 O = 147.0804
1.3678 ppm
175.0756
C 11 H 11 O 2 = 175.0754
1.2603 ppm
215.1068
C 14 H 15 O 2 = 215.1067
0.7928 ppm
275.1279
C 16 H 19 O 4 = 275.1278
0.3020 ppm
389.1748
C 25 H 25 O 4 = 389.1747
0.2556 ppm
431.1853
C 27 H 27 O 5 = 431.1853
0.0762 ppm
499.2335
C 28 H 35 O 8 = 499.2326
1.6325 ppm
0
20
40
60
80
100
150
200
250
300
350
400
450
500
m/z

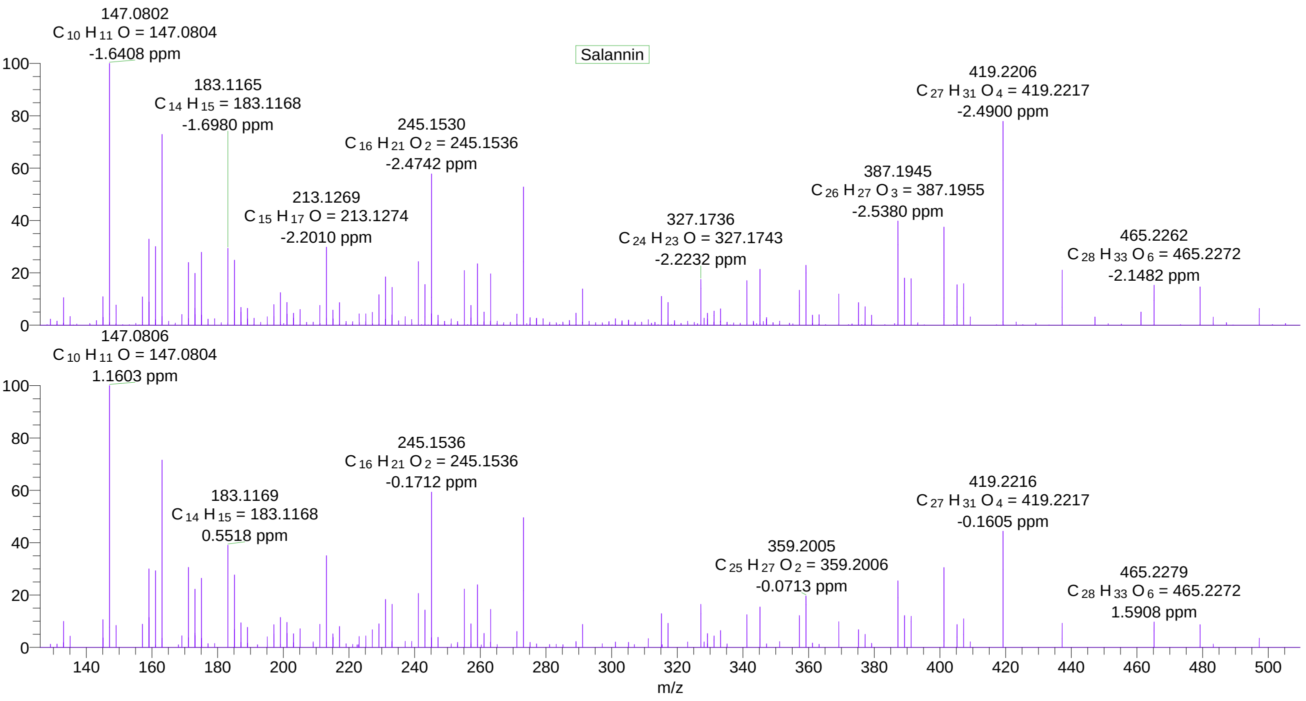

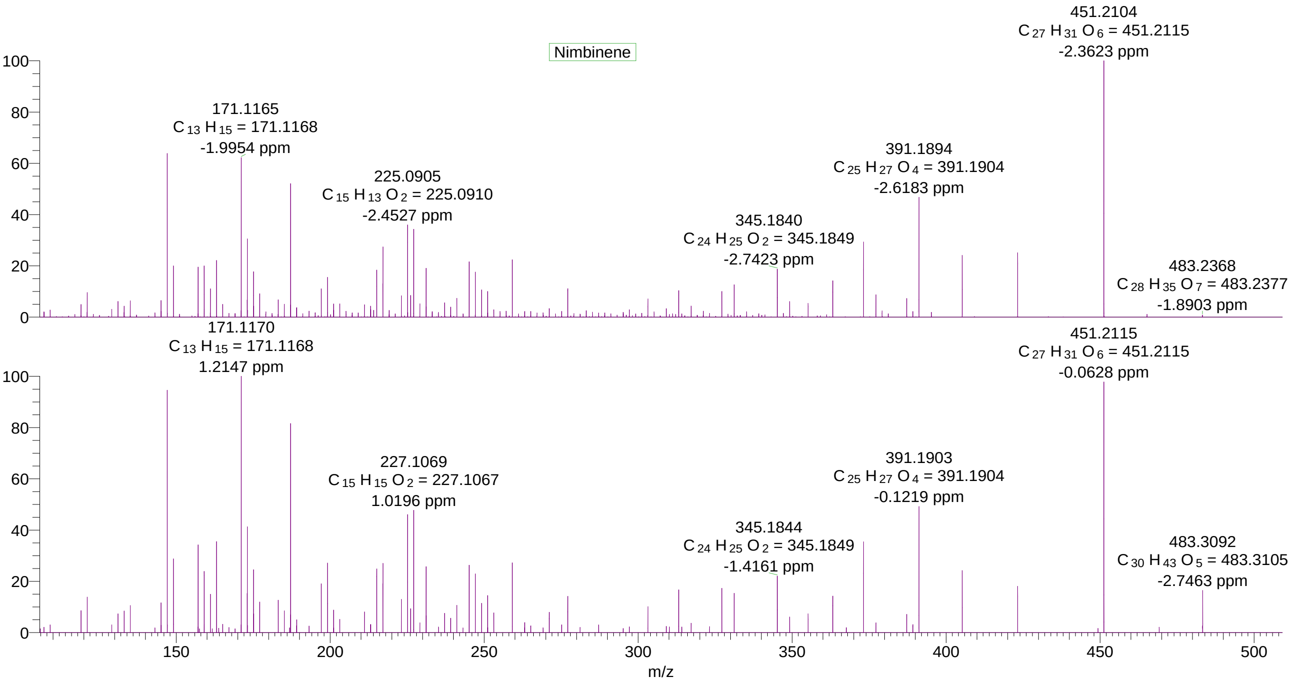

Fig. S1. Overlay of MS/MS spectra of 9 limonoids standards (bottom) and the limonoids biosynthesized from cell culture (top).

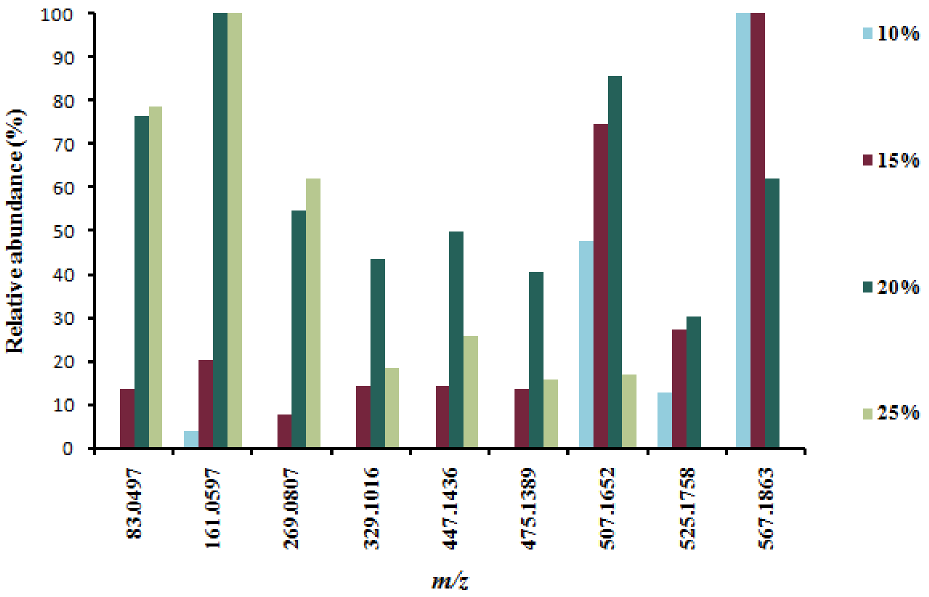

Relative abundance (%)
100
90
80
70
60
50
40
30
20
10
0
83.0497
161.0597
269.0807
329.1016
447.1436
475.1389
507.1652
525.1758
567.1863
m/z
10%
15%
20%
25%

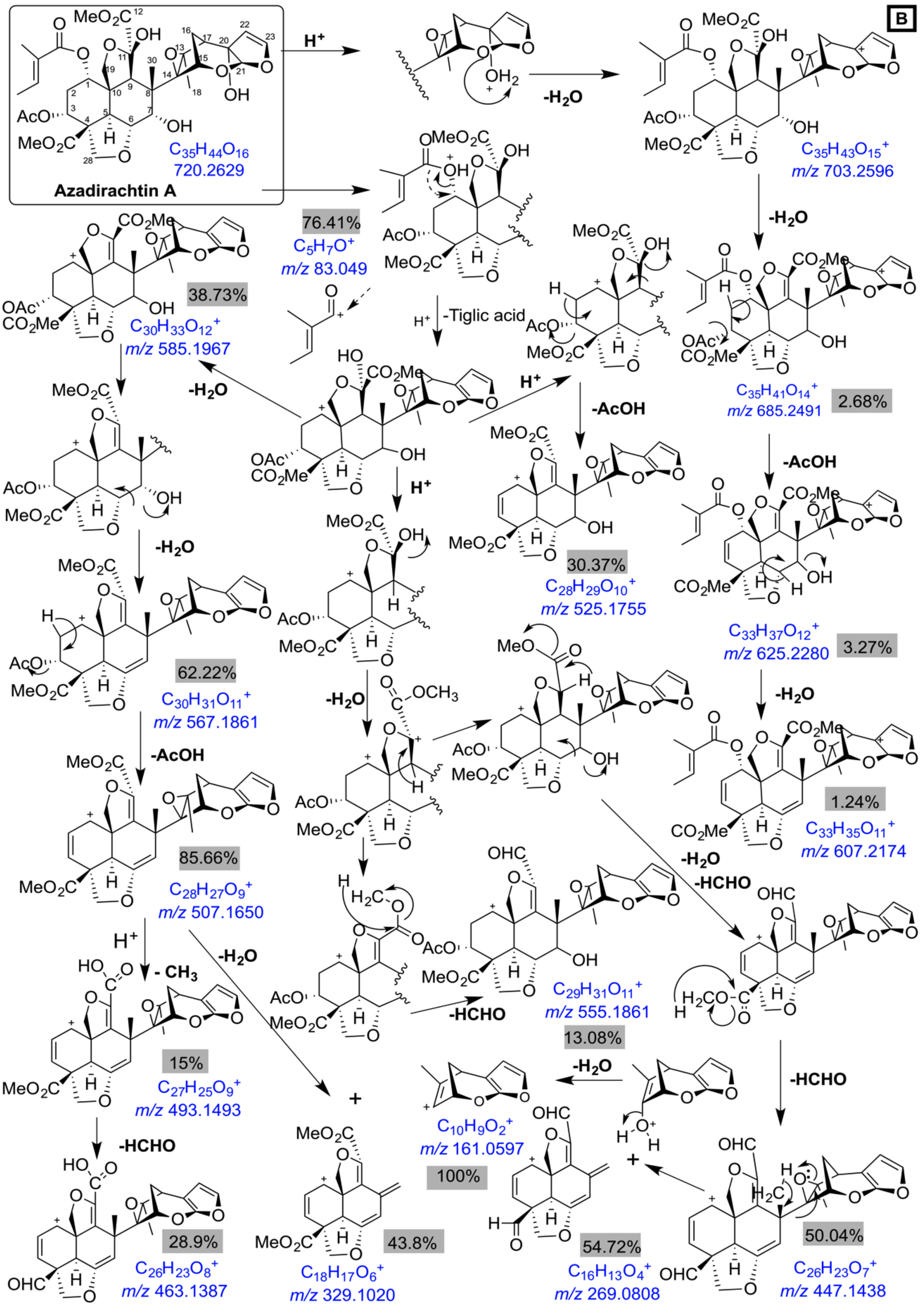

Fig. S2. (a) Variation in relative abundance of MS/MS daughter ions obtained at varying normalized collision energies (NCEs) 10, 15, 20, 25% for azadirachtin A. (b) The structure-fragment relationship of azadirachtin A was inferred from the tandem MS fragmentation

studies of five azadirachtin derivatives at various NCEs based on which the putative fragmentation pathway of azadirachtin A was extrapolated.

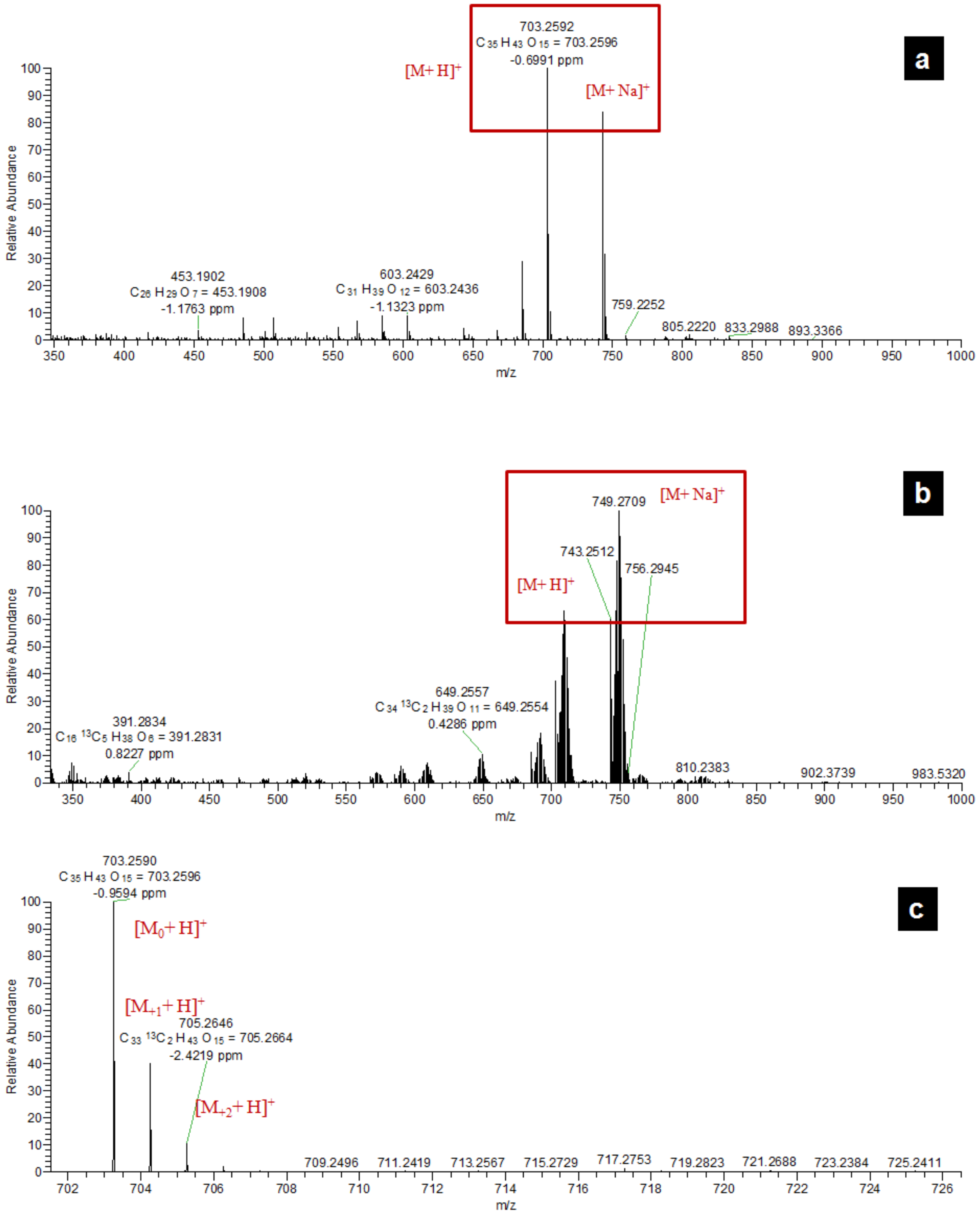

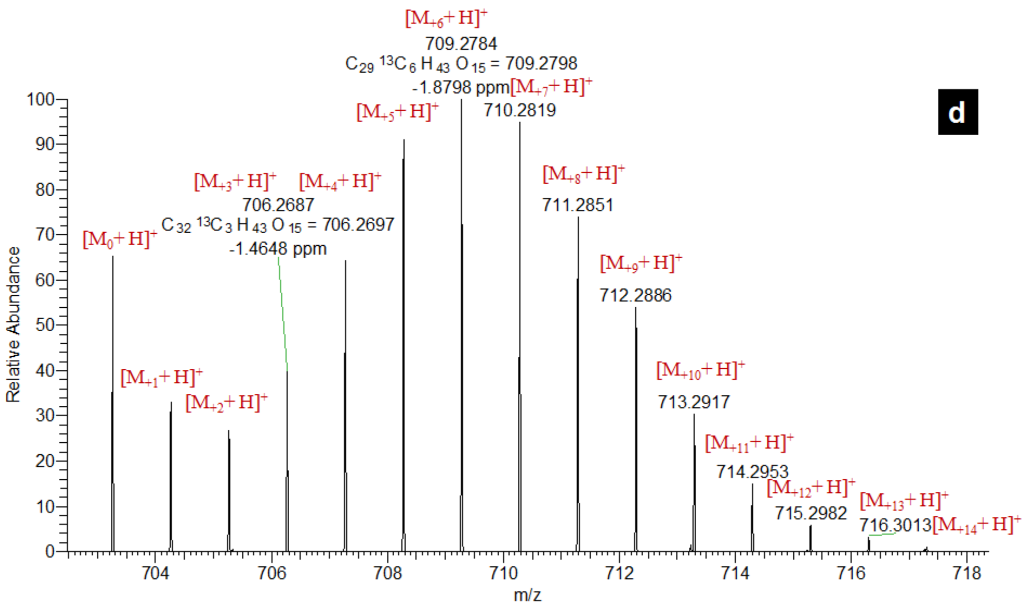
d
[M+6+ H]+
709.2784
C29 13C6 H43 O15 = 709.2798
-1.8798 ppm
[M+7+ H]+
710.2819
[M+5+ H]+
[M+3+ H]+
[M+4+ H]+
706.2687
C32 13C3 H43 O15 = 706.2697
-1.4648 ppm
[M0+ H]+
[M+1+ H]+
[M+2+ H]+
[M+8+ H]+
711.2851
[M+9+ H]+
712.2886
[M+10+ H]+
713.2917
[M+11+ H]+
714.2953
[M+12+ H]+
715.2982
[M+13+ H]+
716.3013
[M+14+ H]+
Relative Abundance
m/z

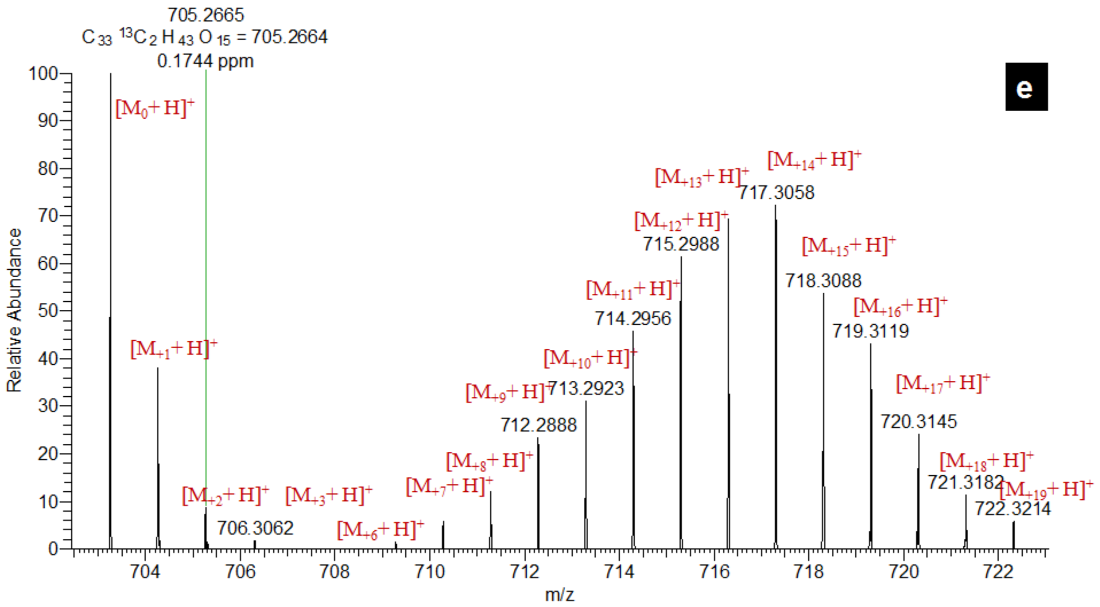
e
705.2665
C33 13C2 H43 O15 = 705.2664
0.1744 ppm
[M0+ H]+
[M+1+ H]+
[M+2+ H]+
706.3062
[M+3+ H]+
[M+6+ H]+
[M+7+ H]+
[M+8+ H]+
[M+9+ H]+
712.2888
[M+10+ H]+
713.2923
[M+11+ H]+
714.2956
[M+12+ H]+
715.2988
[M+13+ H]+
[M+14+ H]+
717.3058
[M+15+ H]+
718.3088
[M+16+ H]+
719.3119
[M+17+ H]+
720.3145
[M+18+ H]+
721.3182
[M+19+ H]+
722.3214
Relative Abundance
m/z

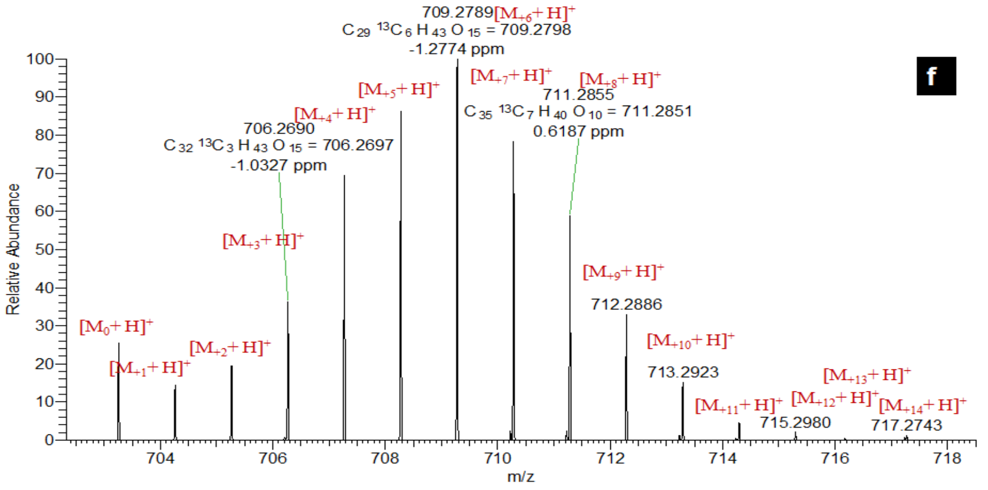
f
709.2789
[M+6+ H]+
C29 13C6 H43 O15 = 709.2798
-1.2774 ppm
[M+7+ H]+
[M+8+ H]+
711.2855
C35 13C7 H40 O10 = 711.2851
0.6187 ppm
[M+5+ H]+
[M+4+ H]+
706.2690
C32 13C3 H43 O15 = 706.2697
-1.0327 ppm
[M+3+ H]+
[M0+ H]+
[M+1+ H]+
[M+2+ H]+
[M+9+ H]+
712.2886
[M+10+ H]+
713.2923
[M+11+ H]+
[M+12+ H]+
715.2980
[M+13+ H]+
[M+14+ H]+
717.2743
Relative Abundance
m/z

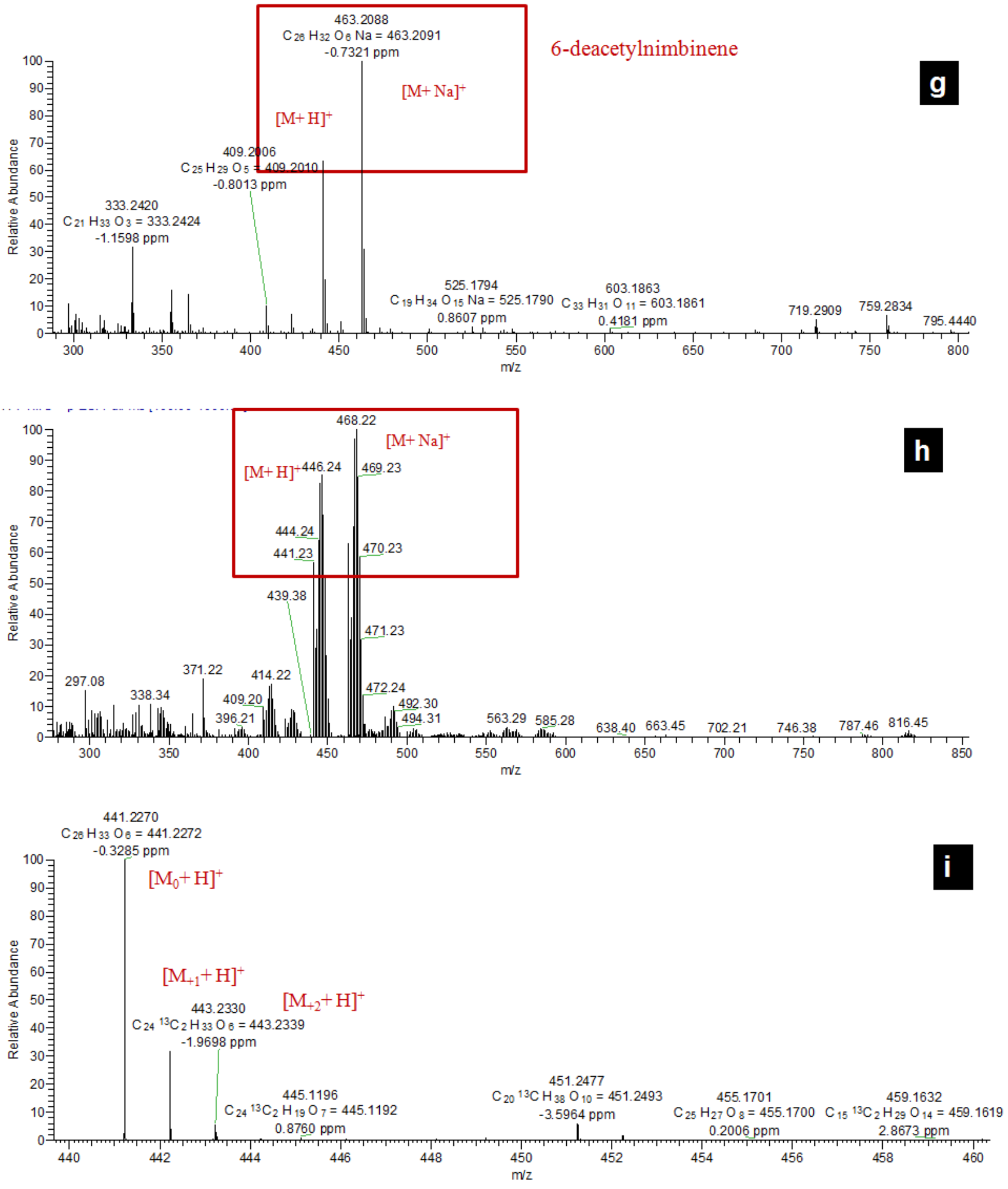
6-deacetylnimbinene
g
463.2088
C26 H32 O6 Na = 463.2091
-0.7321 ppm
[M+ Na]+
[M+ H]+
409.2006
C25 H29 O5 = 409.2010
-0.8013 ppm
333.2420
C21 H33 O3 = 333.2424
-1.1598 ppm
525.1794
C19 H34 O15 Na = 525.1790
0.8607 ppm
603.1863
C33 H31 O11 = 603.1861
0.4181 ppm
719.2909
759.2834
795.4440
Relative Abundance
m/z
h
468.22
[M+ Na]+
[M+ H]+
446.24
469.23
444.24
441.23
470.23
439.38
471.23
297.08
371.22
414.22
472.24
338.34
409.20
492.30
396.21
494.31
563.29
585.28
638.40
663.45
702.21
746.38
787.46
816.45
Relative Abundance
m/z
i
441.2270
C26 H33 O6 = 441.2272
-0.3285 ppm
[M0+ H]+
[M+1+ H]+
[M+2+ H]+
443.2330
C24 13C2 H33 O6 = 443.2339
-1.9698 ppm
445.1196
C24 13C2 H19 O7 = 445.1192
0.8760 ppm
451.2477
C20 13C H38 O10 = 451.2493
-3.5964 ppm
455.1701
C25 H27 O8 = 455.1700
0.2006 ppm
459.1632
C15 13C2 H29 O14 = 459.1619
2.8673 ppm
Relative Abundance
m/z

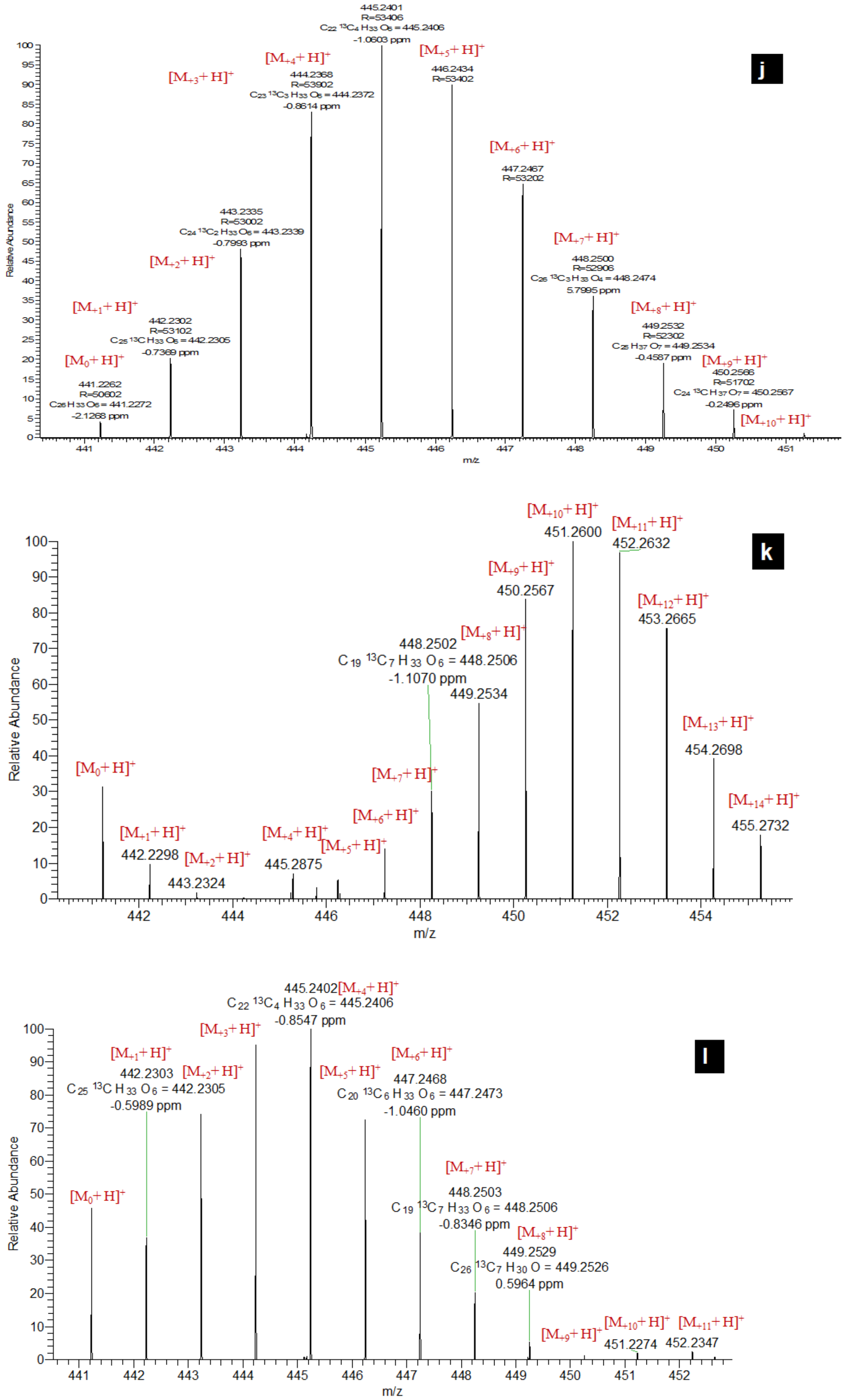
j
k
l
Relative Abundance
m/z
[M0+ H]+
441.2262
R=50602
C26 H33 O6 = 441.2272
-2.1268 ppm
[M+1+ H]+
442.2302
R=53102
C25 13C H33 O6 = 442.2305
-0.7369 ppm
[M+2+ H]+
443.2335
R=53002
C24 13C2 H33 O6 = 443.2339
-0.7993 ppm
[M+3+ H]+
[M+4+ H]+
444.2368
R=53902
C23 13C3 H33 O6 = 444.2372
-0.8614 ppm
445.2401
R=53406
C22 13C4 H33 O6 = 445.2406
-1.0603 ppm
[M+5+ H]+
446.2434
R=53402
[M+6+ H]+
447.2467
R=53202
[M+7+ H]+
448.2500
R=52906
C26 13C3 H33 O4 = 448.2474
5.7995 ppm
[M+8+ H]+
449.2532
R=52302
C25 H37 O7 = 449.2534
-0.4587 ppm
[M+9+ H]+
450.2566
R=51702
C24 13C H37 O7 = 450.2567
-0.2496 ppm
[M+10+ H]+
442.2298
443.2324
445.2875
448.2502
C 19 13C7 H 33 O 6 = 448.2506
-1.1070 ppm
449.2534
450.2567
451.2600
[M+11+ H]+
452.2632
[M+12+ H]+
453.2665
[M+13+ H]+
454.2698
[M+14+ H]+
455.2732
445.2402
C 22 13C4 H 33 O 6 = 445.2406
-0.8547 ppm
442.2303
C 25 13C H 33 O 6 = 442.2305
-0.5989 ppm
447.2468
C 20 13C6 H 33 O 6 = 447.2473
-1.0460 ppm
448.2503
C 19 13C7 H 33 O 6 = 448.2506
-0.8346 ppm
449.2529
C 26 13C7 H 30 O = 449.2526
0.5964 ppm
451.2274
452.2347

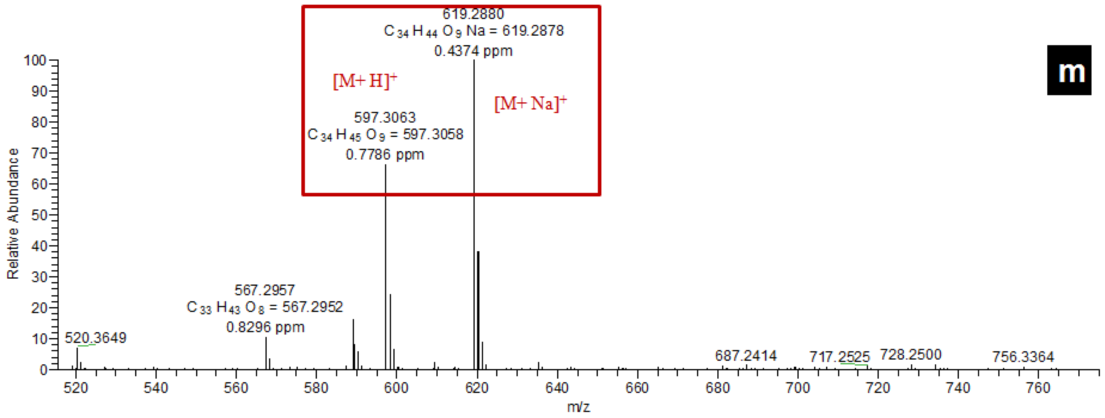
m
619.2880
C 34 H 44 O 9 Na = 619.2878
0.4374 ppm
[M+ H]+
[M+ Na]+
597.3063
C 34 H 45 O 9 = 597.3058
0.7786 ppm
567.2957
C 33 H 43 O 8 = 567.2952
0.8296 ppm
520.3649
687.2414
717.2525
728.2500
756.3364
Relative Abundance
m/z

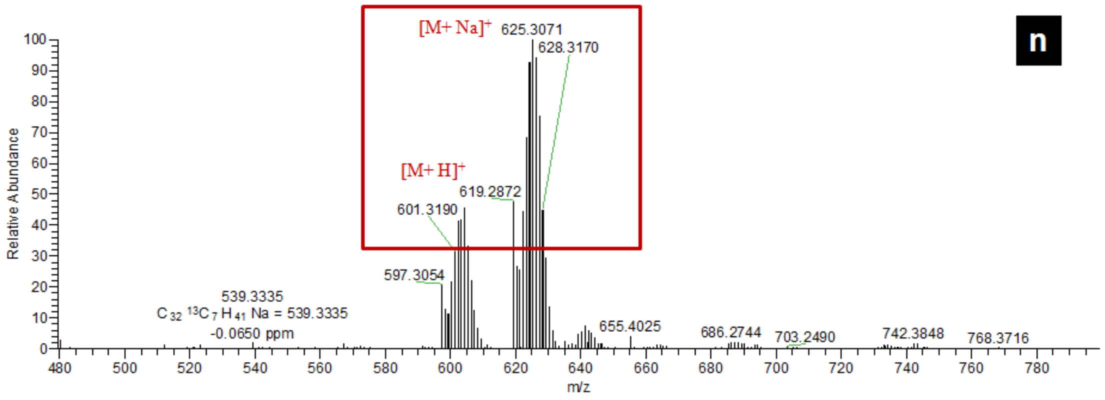
n
[M+ Na]+
625.3071
628.3170
[M+ H]+
619.2872
601.3190
597.3054
539.3335
C 32 13C 7 H 41 Na = 539.3335
-0.0650 ppm
655.4025
686.2744
703.2490
742.3848
768.3716
Relative Abundance
m/z

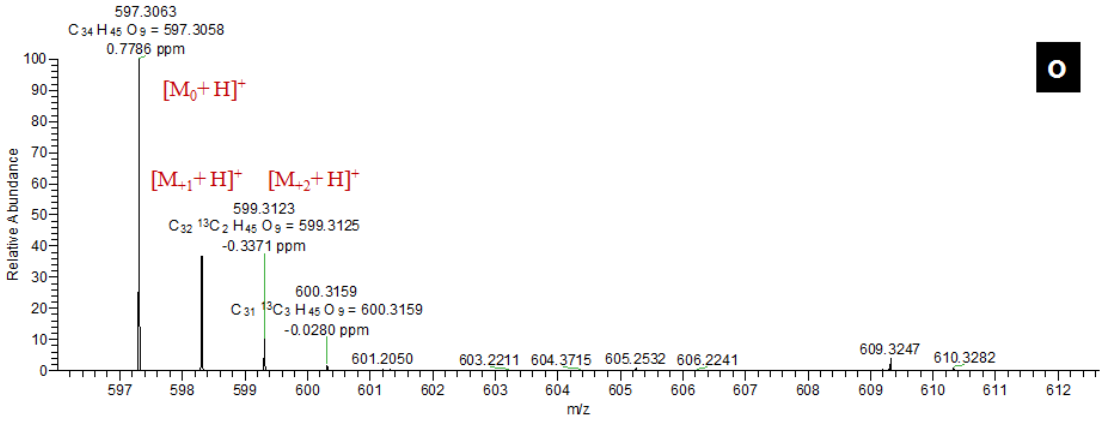
o
597.3063
C 34 H 45 O 9 = 597.3058
0.7786 ppm
[M0+ H]+
[M+1+ H]+
[M+2+ H]+
599.3123
C 32 13C 2 H 45 O 9 = 599.3125
-0.3371 ppm
600.3159
C 31 13C 3 H 45 O 9 = 600.3159
-0.0280 ppm
601.2050
603.2211
604.3715
605.2532
606.2241
609.3247
610.3282
Relative Abundance
m/z

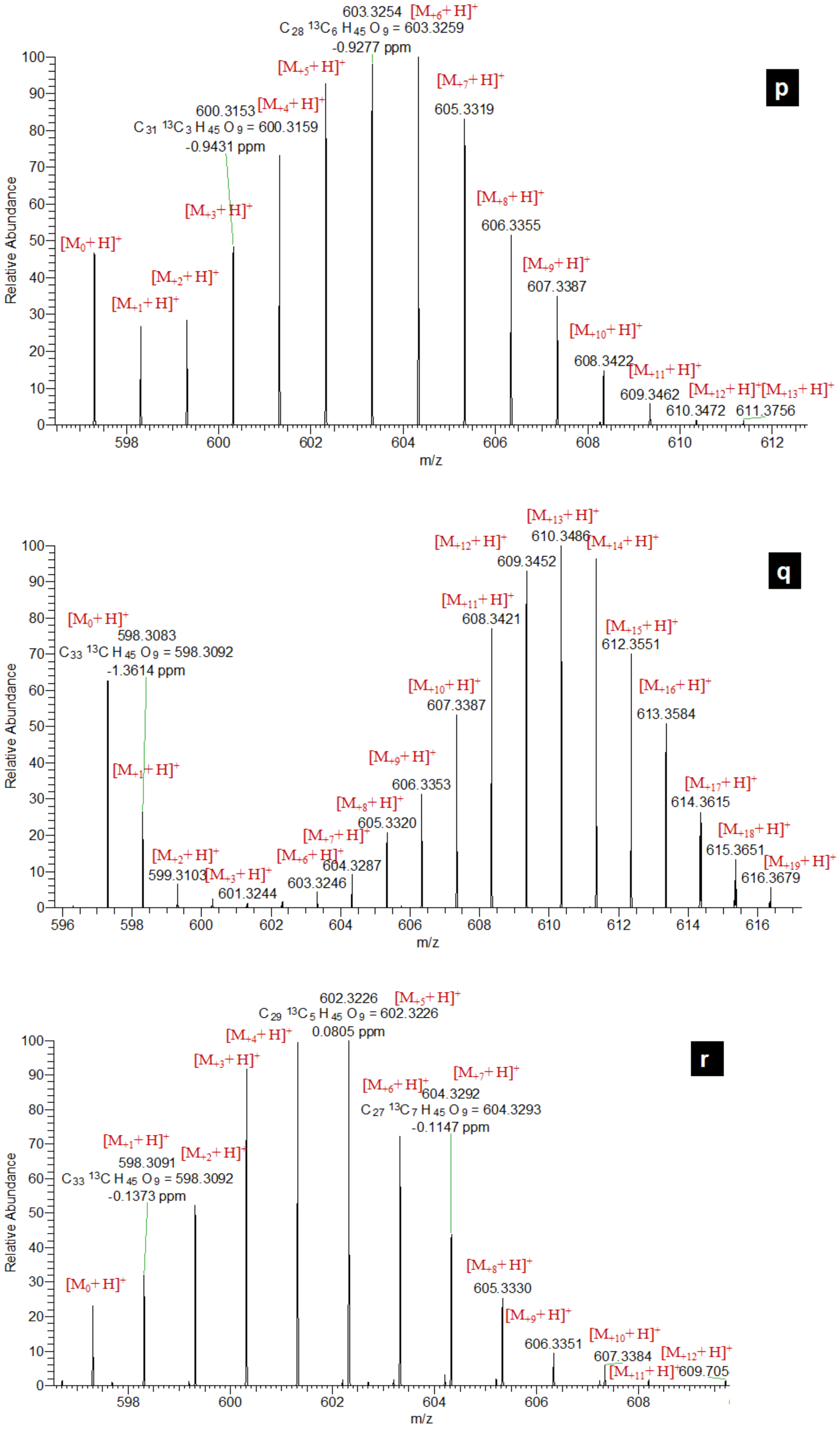
p
603.3254 [M+6+H]+
C 28 13C6 H 45 O 9 = 603.3259
-0.9277 ppm
[M+5+H]+
[M+4+H]+
600.3153
C 31 13C 3 H 45 O 9 = 600.3159
-0.9431 ppm
[M+7+H]+
605.3319
[M+3+H]+
[M0+H]+
[M+2+H]+
[M+1+H]+
[M+8+H]+
606.3355
[M+9+H]+
607.3387
[M+10+H]+
608.3422
[M+11+H]+
609.3462
[M+12+H]+
[M+13+H]+
610.3472
611.3756
Relative Abundance
m/z
q
[M+13+H]+
610.3486
[M+12+H]+
609.3452
[M+14+H]+
[M+11+H]+
608.3421
[M0+H]+
598.3083
C33 13C H 45 O 9 = 598.3092
-1.3614 ppm
[M+15+H]+
612.3551
[M+10+H]+
607.3387
[M+16+H]+
613.3584
[M+9+H]+
606.3353
[M+1+H]+
[M+17+H]+
614.3615
[M+8+H]+
605.3320
[M+7+H]+
[M+18+H]+
615.3651
[M+2+H]+
[M+6+H]+
604.3287
[M+19+H]+
599.3103
[M+3+H]+
603.3246
616.3679
601.3244
Relative Abundance
m/z
r
602.3226 [M+5+H]+
C 29 13C 5 H 45 O 9 = 602.3226
0.0805 ppm
[M+4+H]+
[M+3+H]+
[M+6+H]+
604.3292
[M+7+H]+
C 27 13C 7 H 45 O 9 = 604.3293
-0.1147 ppm
[M+1+H]+
598.3091
[M+2+H]+
C33 13C H 45 O 9 = 598.3092
-0.1373 ppm
[M0+H]+
[M+8+H]+
605.3330
[M+9+H]+
606.3351
[M+10+H]+
607.3384
[M+12+H]+
[M+11+H]+
609.705
Relative Abundance
m/z

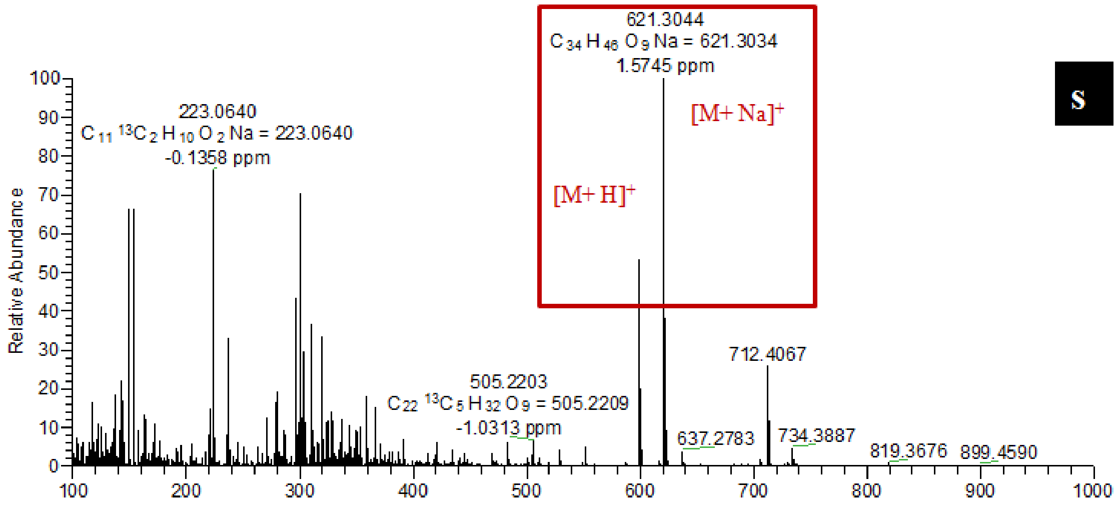
s
621.3044
C 34 H 46 O 9 Na = 621.3034
1.5745 ppm
[M+ Na]+
[M+ H]+
223.0640
C 11 13C 2 H 10 O 2 Na = 223.0640
-0.1358 ppm
505.2203
C 22 13C 5 H 32 O 9 = 505.2209
-1.0313 ppm
712.4067
637.2783
734.3887
819.3676
899.4590
Relative Abundance

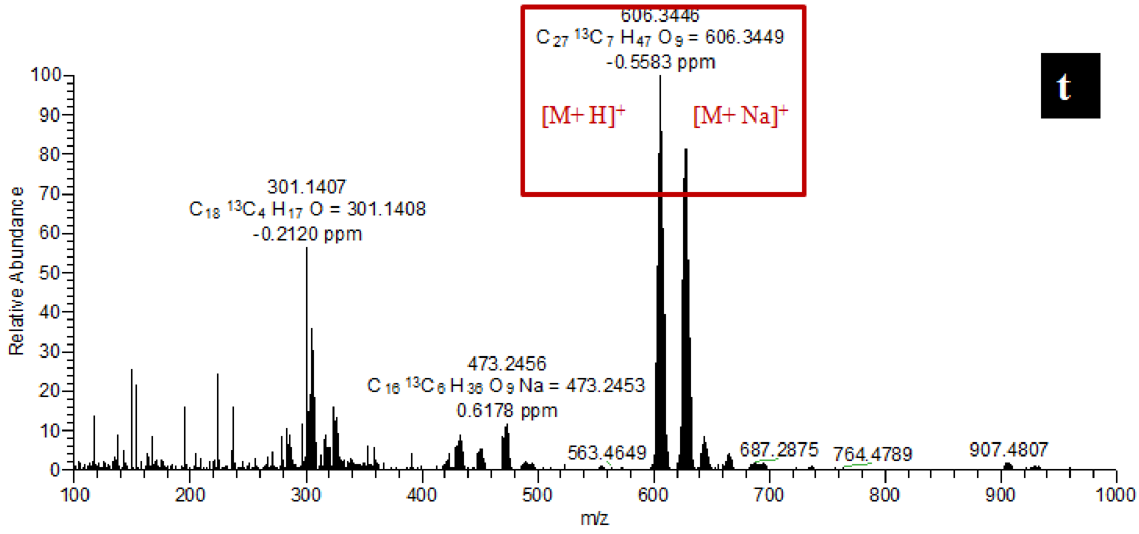
t
606.3446
C 27 13C 7 H 47 O 9 = 606.3449
-0.5583 ppm
[M+ H]+
[M+ Na]+
301.1407
C 18 13C 4 H 17 O = 301.1408
-0.2120 ppm
473.2456
C 16 13C 6 H 36 O 9 Na = 473.2453
0.6178 ppm
563.4649
687.2875
764.4789
907.4807
Relative Abundance
m/z

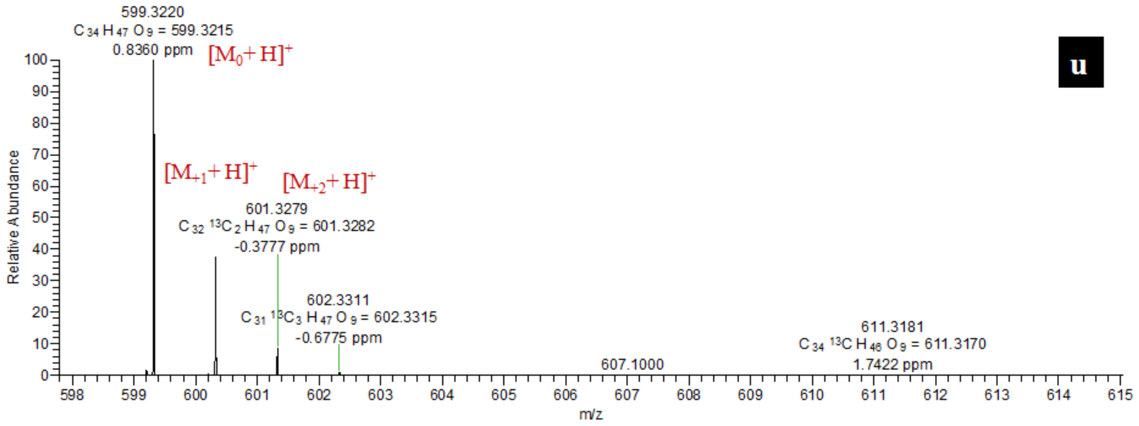
u
599.3220
C 34 H 47 O 9 = 599.3215
0.8360 ppm
[M0+ H]+
[M+1+ H]+
[M+2+ H]+
601.3279
C 32 13C 2 H 47 O 9 = 601.3282
-0.3777 ppm
602.3311
C 31 13C 3 H 47 O 9 = 602.3315
-0.6775 ppm
607.1000
611.3181
C 34 13C H 46 O 9 = 611.3170
1.7422 ppm
Relative Abundance
m/z

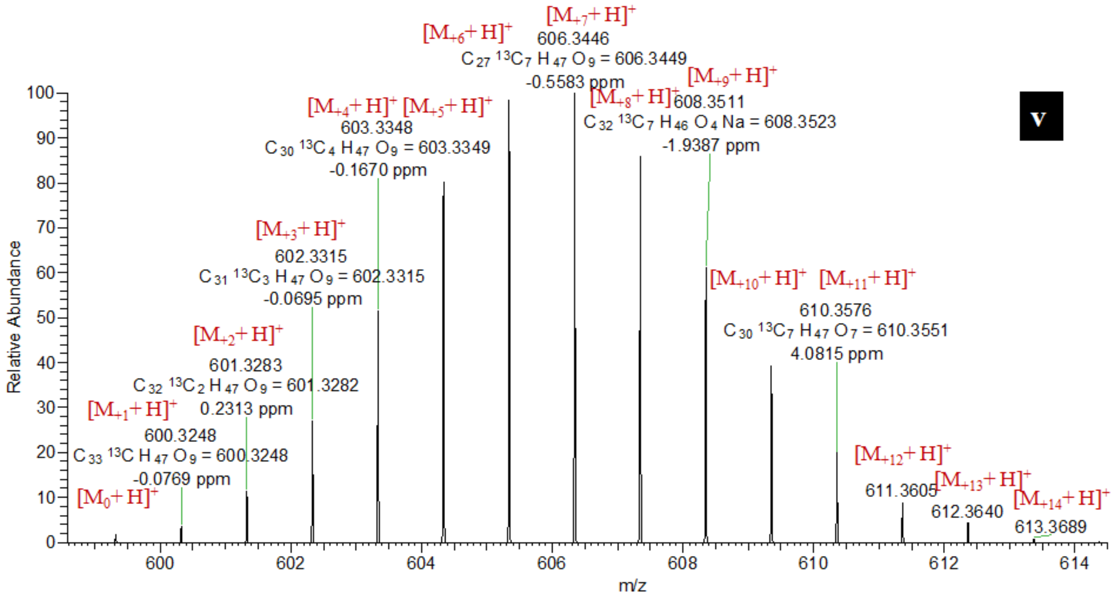
v
Relative Abundance
m/z
[M0+ H]+
[M+1+ H]+
600.3248
C33 13C H47 O9 = 600.3248
-0.0769 ppm
[M+2+ H]+
601.3283
C32 13C2 H47 O9 = 601.3282
0.2313 ppm
[M+3+ H]+
602.3315
C31 13C3 H47 O9 = 602.3315
-0.0695 ppm
[M+4+ H]+ [M+5+ H]+
603.3348
C30 13C4 H47 O9 = 603.3349
-0.1670 ppm
[M+6+ H]+
[M+7+ H]+
606.3446
C27 13C7 H47 O9 = 606.3449
-0.5583 ppm
[M+8+ H]+ 608.3511
[M+9+ H]+
C32 13C7 H46 O4 Na = 608.3523
-1.9387 ppm
[M+10+ H]+ [M+11+ H]+
610.3576
C30 13C7 H47 O7 = 610.3551
4.0815 ppm
[M+12+ H]+
611.3605
[M+13+ H]+
612.3640
[M+14+ H]+
613.3689

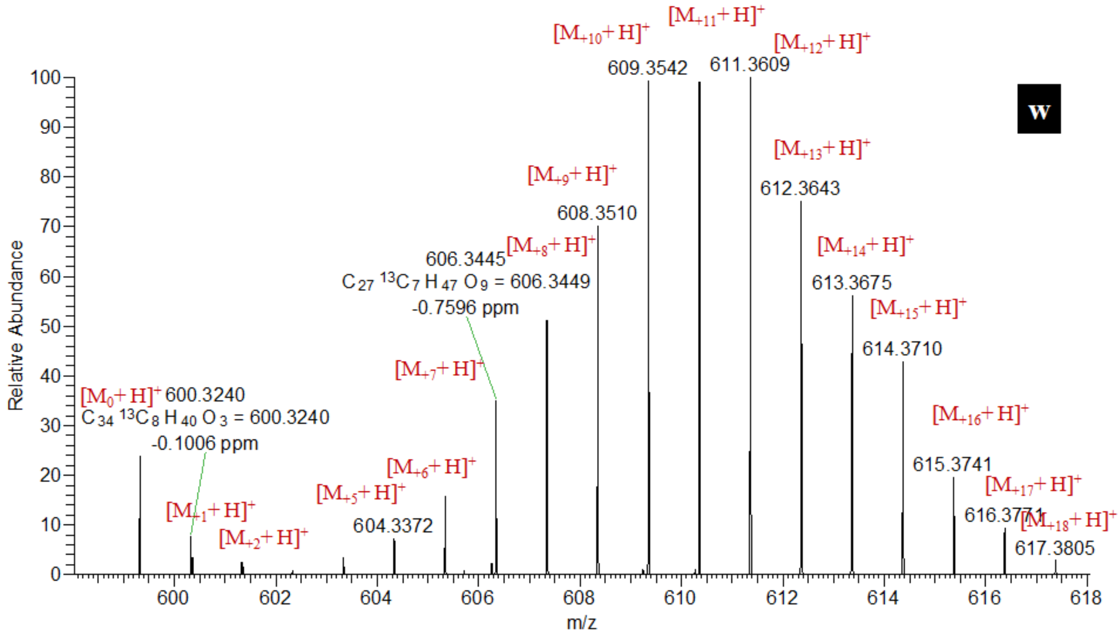
w
Relative Abundance
m/z
[M0+ H]+ 600.3240
C34 13C8 H40 O3 = 600.3240
-0.1006 ppm
[M+1+ H]+
[M+2+ H]+
[M+5+ H]+
604.3372
[M+6+ H]+
[M+7+ H]+
606.3445
C27 13C7 H47 O9 = 606.3449
-0.7596 ppm
[M+8+ H]+
[M+9+ H]+
608.3510
[M+10+ H]+
609.3542
[M+11+ H]+
611.3609
[M+12+ H]+
[M+13+ H]+
612.3643
[M+14+ H]+
613.3675
[M+15+ H]+
614.3710
[M+16+ H]+
615.3741
[M+17+ H]+
616.3771
[M+18+ H]+
617.3805

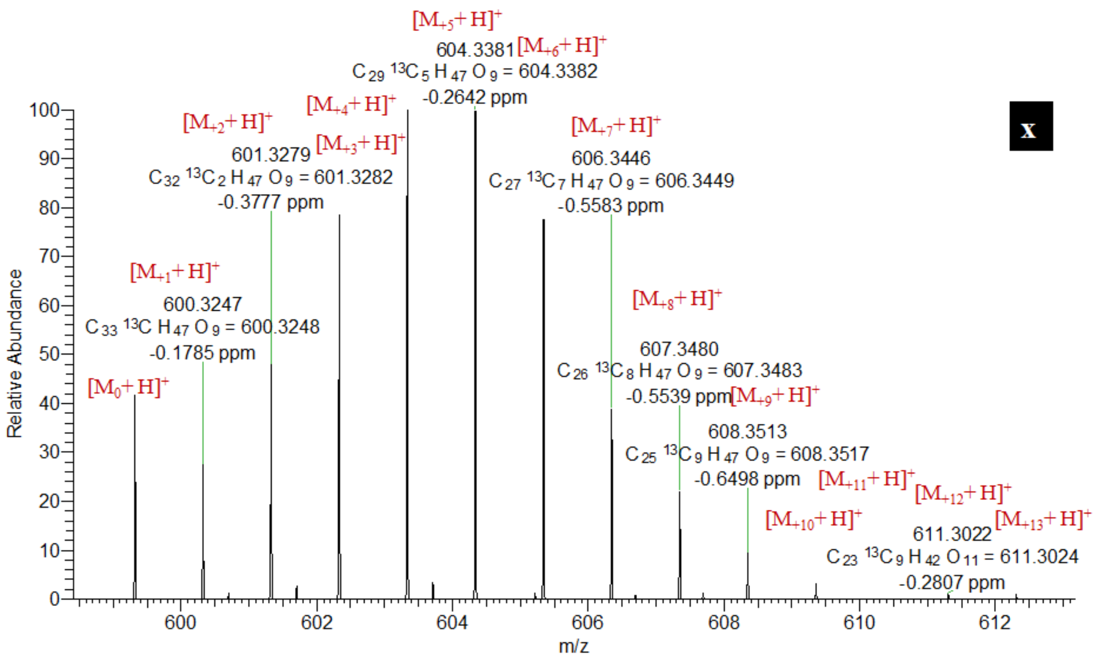

Fig. S3. Comparison of mass spectra of the unlabeled and ^{13}C limonoids from the neem cell suspension (a) azadirachtin A. (b) labeled azadirachtin A. (c) Natural abundance ^{13}C of protonated molecular ion of azadirachtin A. (d) 1-^{13}C Glc labeling of azadirachtin A. (e) 1,6-^{13}C Glc labeling of azadirachtin A. (f) 2-^{13}C Glc labeling of azadirachtin A. (g) 6-deacetylnimbinene. (h) labeled 6-deacetylnimbinene. (i) Natural abundance ^{13}C of protonated molecular ion of 6-deacetylnimbinene. (j) 1-^{13}C Glc labeling of 6-deacetylnimbinene. (k) 1,6-^{13}C Glc labeling of 6-deacetylnimbinene. (l) 2-^{13}C Glc labeling of 6-deacetylnimbinene. (m) salannin. (n) labeled salannin. (o) Natural abundance ^{13}C of protonated molecular ion of salannin. (p) 1-^{13}C Glc labeling of salannin. (q) 1,6-^{13}C Glc labeling of salannin. (r) 2-^{13}C Glc labeling of salannin. (s) salannolacetate. (t) labeled salannolacetate. (u) Natural abundance ^{13}C of protonated molecular ion of salannolacetate. (v) 1-^{13}C Glc labeling of salannolacetate. (w) 1,6-^{13}C Glc labeling of salannolacetate. (x) 2-^{13}C Glc labeling of salannolacetate.

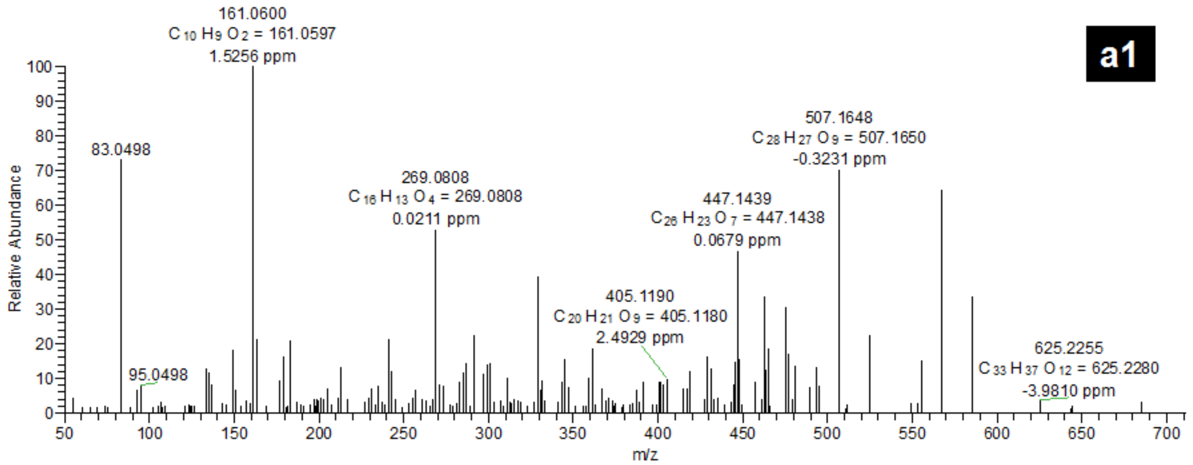

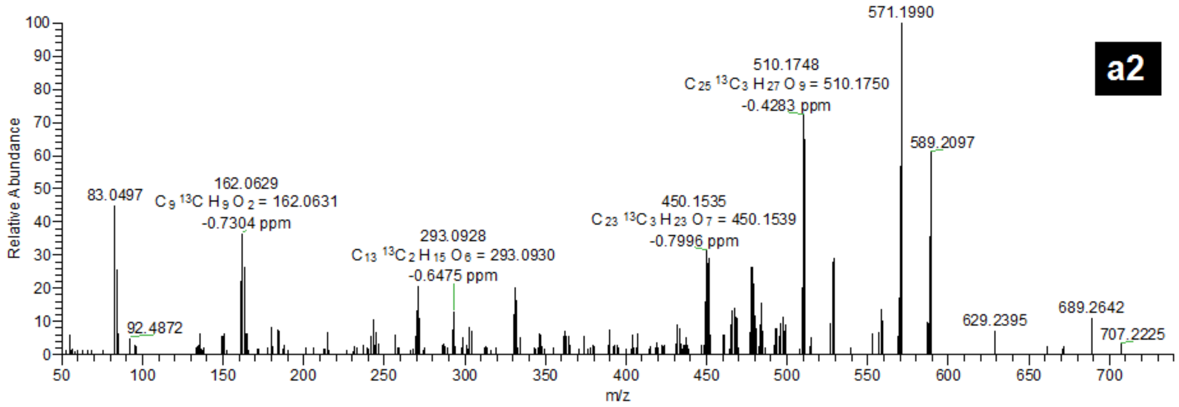
a2
Relative Abundance
m/z
571.1990
510.1748
C25 13C3 H27 O9 = 510.1750
-0.4283 ppm
589.2097
450.1535
C23 13C3 H23 O7 = 450.1539
-0.7996 ppm
83.0497
162.0629
C9 13C H9 O2 = 162.0631
-0.7304 ppm
293.0928
C13 13C2 H15 O6 = 293.0930
-0.6475 ppm
92.4872
629.2395
689.2642
707.2225

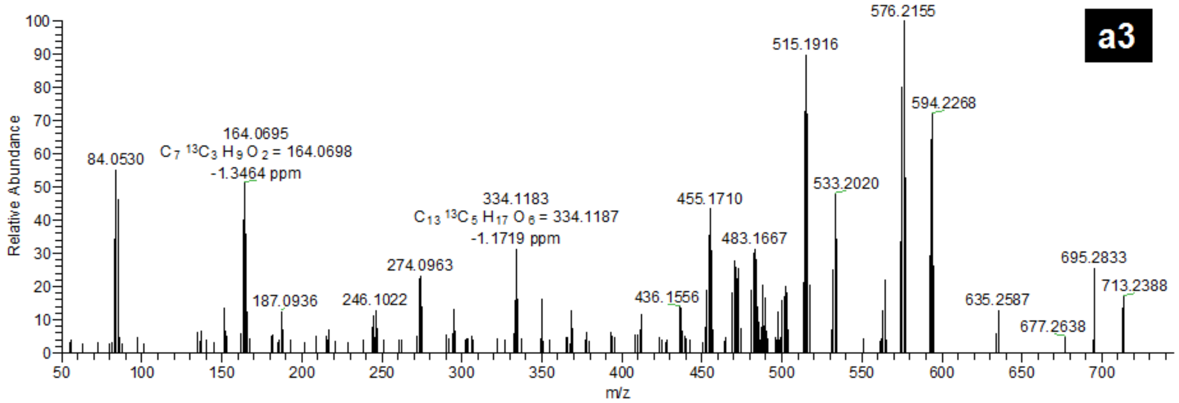
a3
Relative Abundance
m/z
576.2155
515.1916
594.2268
84.0530
164.0695
C7 13C3 H9 O2 = 164.0698
-1.3464 ppm
533.2020
334.1183
C13 13C5 H17 O6 = 334.1187
-1.1719 ppm
455.1710
483.1667
274.0963
695.2833
713.2388
187.0936
246.1022
436.1556
635.2587
677.2638

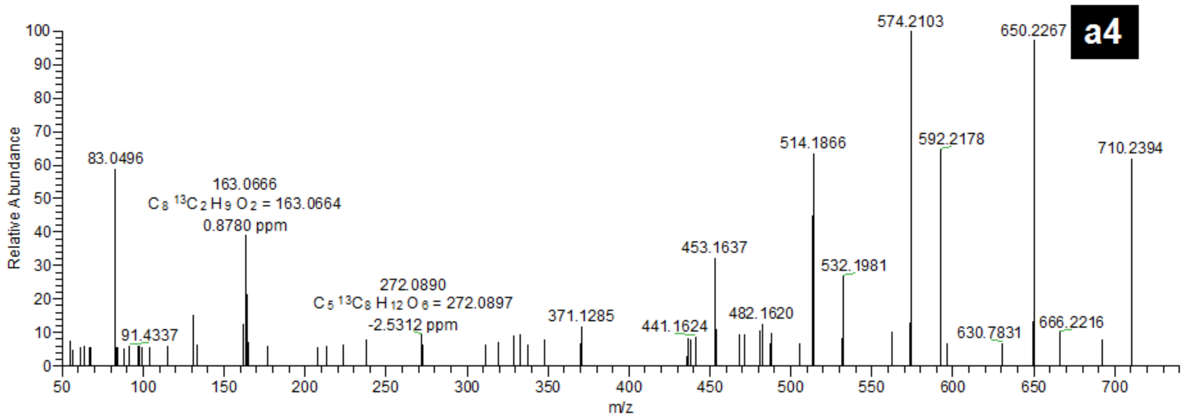
a4
Relative Abundance
m/z
574.2103
650.2267
514.1866
592.2178
710.2394
83.0496
163.0666
C8 13C2 H9 O2 = 163.0664
0.8780 ppm
453.1637
532.1981
272.0890
C5 13C8 H12 O6 = 272.0897
-2.5312 ppm
371.1285
441.1624
482.1620
91.4337
630.7831
666.2216

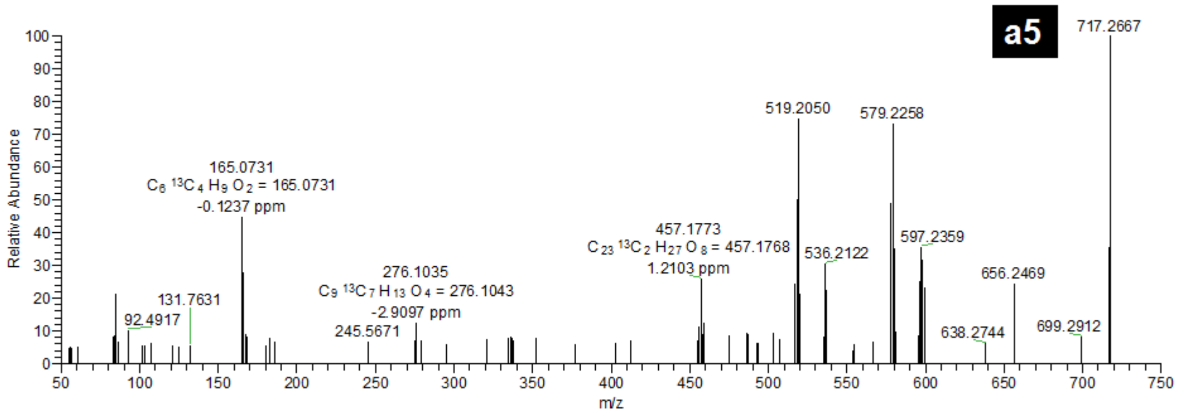
a5
Relative Abundance
m/z
717.2667
519.2050
579.2258
165.0731
C6 13C4 H9 O2 = 165.0731
-0.1237 ppm
457.1773
C23 13C2 H27 O8 = 457.1768
1.2103 ppm
536.2122
597.2359
276.1035
C9 13C7 H13 O4 = 276.1043
-2.9097 ppm
656.2469
131.7631
92.4917
245.5671
638.2744
699.2912

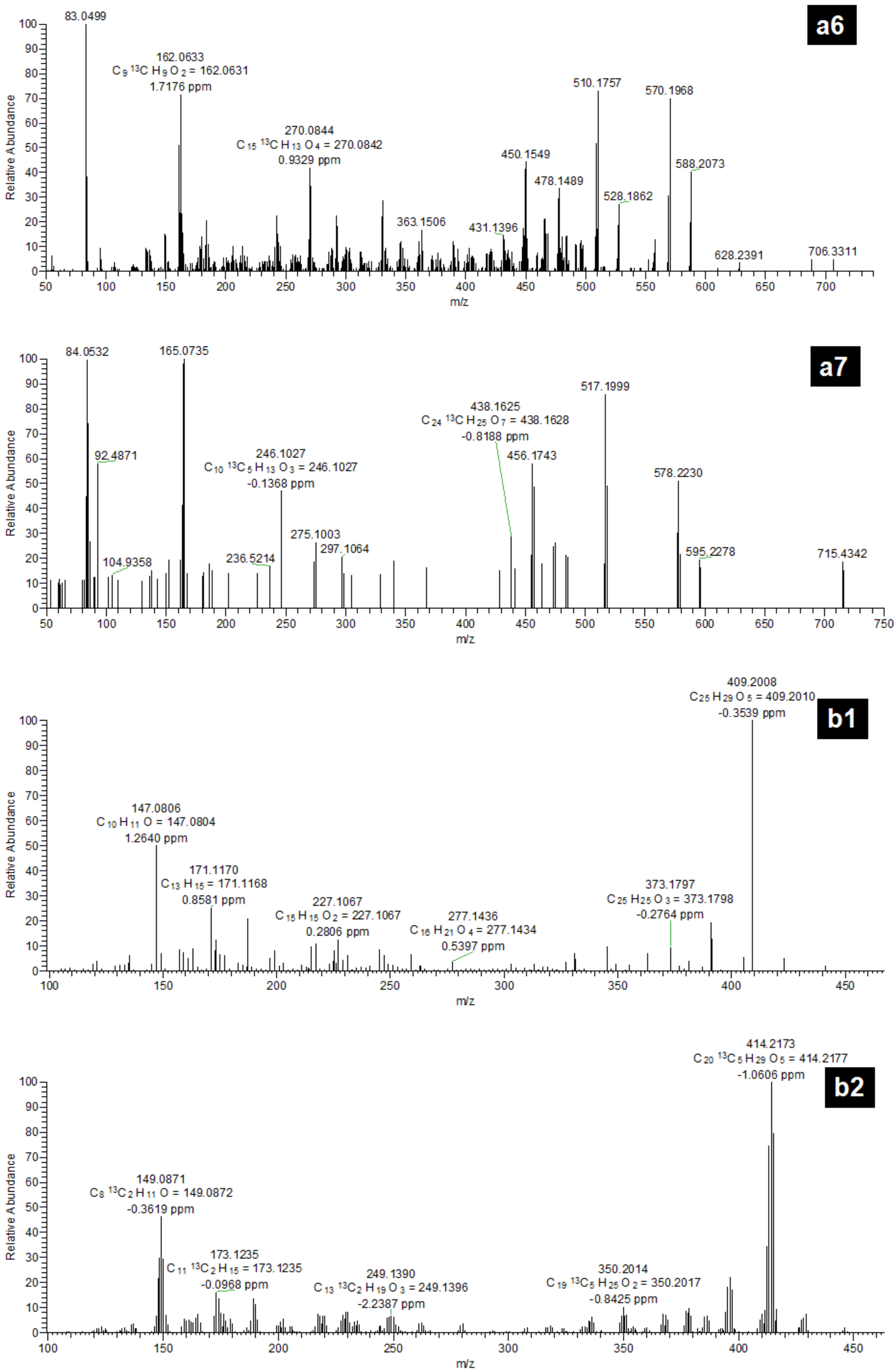
a6
83.0499
162.0633
C 9 13C H 9 O 2 = 162.0631
1.7176 ppm
270.0844
C 15 13C H 13 O 4 = 270.0842
0.9329 ppm
363.1506
431.1396
450.1549
478.1489
510.1757
528.1862
570.1968
588.2073
628.2391
706.3311
Relative Abundance
m/z
a7
84.0532
165.0735
92.4871
104.9358
246.1027
C 10 13C 5 H 13 O 3 = 246.1027
-0.1368 ppm
236.5214
275.1003
297.1064
438.1625
C 24 13C H 25 O 7 = 438.1628
-0.8188 ppm
456.1743
517.1999
578.2230
595.2278
715.4342
Relative Abundance
m/z
b1
409.2008
C 25 H 29 O 5 = 409.2010
-0.3539 ppm
147.0806
C 10 H 11 O = 147.0804
1.2640 ppm
171.1170
C 13 H 15 = 171.1168
0.8581 ppm
227.1067
C 15 H 15 O 2 = 227.1067
0.2806 ppm
277.1436
C 16 H 21 O 4 = 277.1434
0.5397 ppm
373.1797
C 25 H 25 O 3 = 373.1798
-0.2764 ppm
Relative Abundance
m/z
b2
414.2173
C 20 13C 5 H 29 O 5 = 414.2177
-1.0606 ppm
149.0871
C 8 13C 2 H 11 O = 149.0872
-0.3619 ppm
173.1235
C 11 13C 2 H 15 = 173.1235
-0.0968 ppm
249.1390
C 13 13C 2 H 19 O 3 = 249.1396
-2.2387 ppm
350.2014
C 19 13C 5 H 25 O 2 = 350.2017
-0.8425 ppm
Relative Abundance
m/z

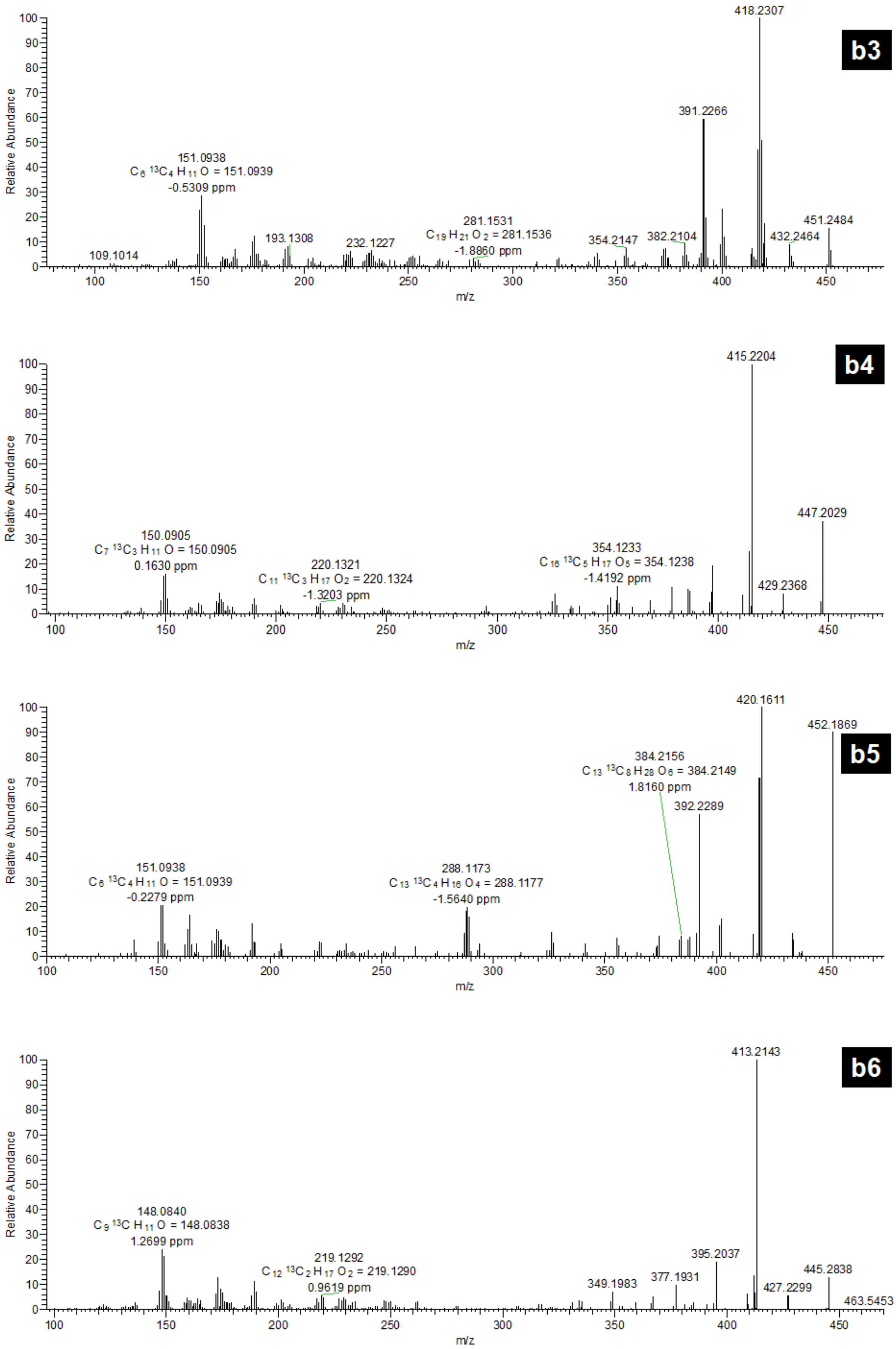

b3
418.2307
391.2266
151.0938
C6 13C4 H11 O = 151.0939
-0.5309 ppm
281.1531
C19 H21 O2 = 281.1536
-1.8860 ppm
193.1308
232.1227
354.2147
382.2104
432.2464
451.2484
109.1014
Relative Abundance
m/z
b4
415.2204
447.2029
150.0905
C7 13C3 H11 O = 150.0905
0.1630 ppm
220.1321
C11 13C3 H17 O2 = 220.1324
-1.3203 ppm
354.1233
C16 13C5 H17 O5 = 354.1238
-1.4192 ppm
429.2368
Relative Abundance
m/z
b5
420.1611
452.1869
384.2156
C13 13C8 H28 O6 = 384.2149
1.8160 ppm
392.2289
151.0938
C6 13C4 H11 O = 151.0939
-0.2279 ppm
288.1173
C13 13C4 H16 O4 = 288.1177
-1.5640 ppm
Relative Abundance
m/z
b6
413.2143
148.0840
C9 13C H11 O = 148.0838
1.2699 ppm
219.1292
C12 13C2 H17 O2 = 219.1290
0.9619 ppm
395.2037
349.1983
377.1931
427.2299
445.2838
463.5453
Relative Abundance
m/z

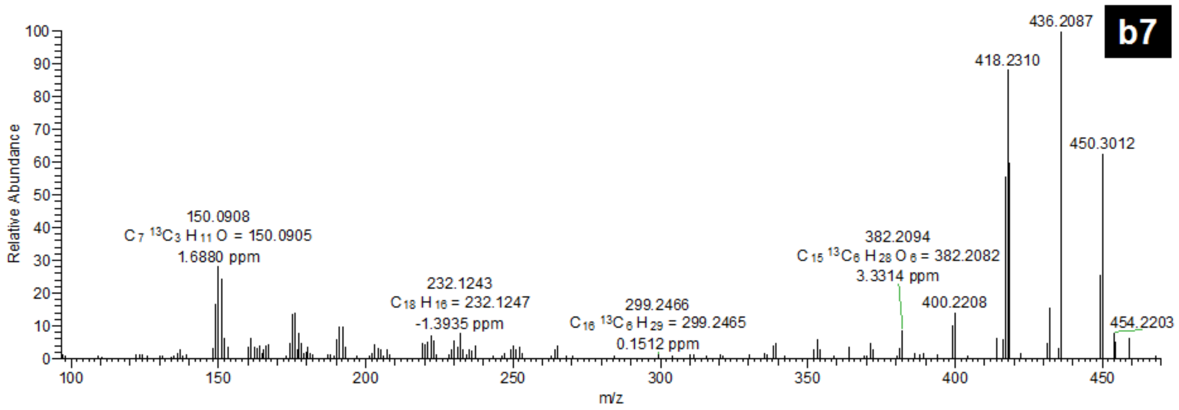
b7
Relative Abundance
m/z
436.2087
418.2310
450.3012
150.0908
$C_7{}^{13}C_3H_{11}O$ = 150.0905
1.6880 ppm
382.2094
$C_{15}{}^{13}C_6H_{28}O_6$ = 382.2082
3.3314 ppm
232.1243
$C_{18}H_{16}$ = 232.1247
-1.3935 ppm
299.2466
$C_{16}{}^{13}C_6H_{29}$ = 299.2465
0.1512 ppm
400.2208
454.2203

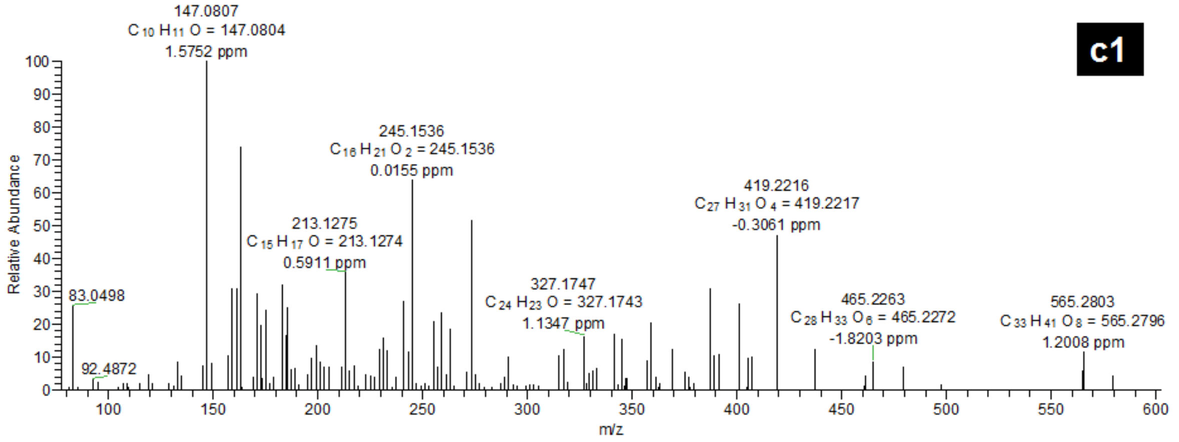
c1
Relative Abundance
m/z
147.0807
$C_{10}H_{11}O$ = 147.0804
1.5752 ppm
245.1536
$C_{16}H_{21}O_2$ = 245.1536
0.0155 ppm
419.2216
$C_{27}H_{31}O_4$ = 419.2217
-0.3061 ppm
213.1275
$C_{15}H_{17}O$ = 213.1274
0.5911 ppm
83.0498
327.1747
$C_{24}H_{23}O$ = 327.1743
1.1347 ppm
465.2263
$C_{28}H_{33}O_6$ = 465.2272
-1.8203 ppm
565.2803
$C_{33}H_{41}O_8$ = 565.2796
1.2008 ppm
92.4872

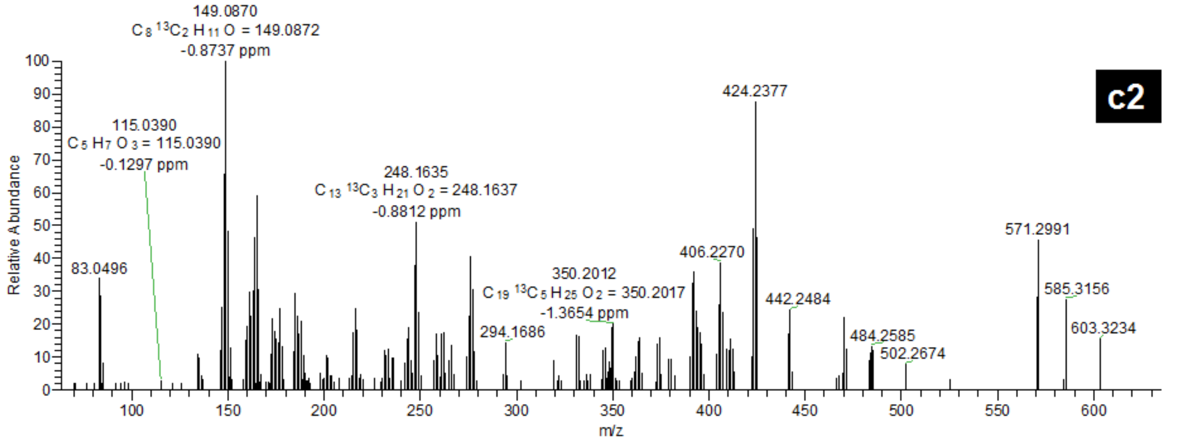
c2
Relative Abundance
m/z
149.0870
$C_8{}^{13}C_2H_{11}O$ = 149.0872
-0.8737 ppm
424.2377
115.0390
$C_5H_7O_3$ = 115.0390
-0.1297 ppm
248.1635
$C_{13}{}^{13}C_3H_{21}O_2$ = 248.1637
-0.8812 ppm
571.2991
406.2270
83.0496
350.2012
$C_{19}{}^{13}C_5H_{25}O_2$ = 350.2017
-1.3654 ppm
585.3156
442.2484
294.1686
484.2585
603.3234
502.2674

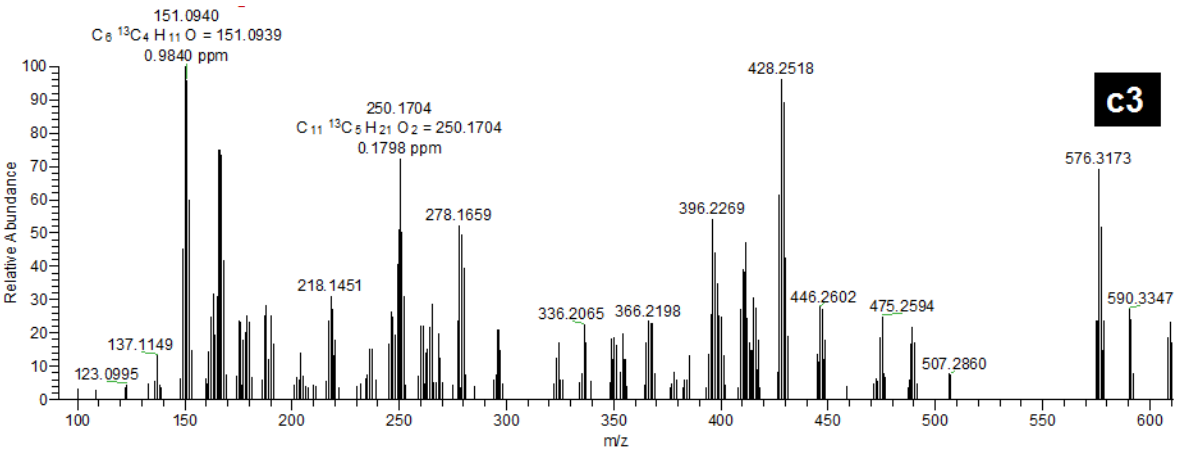
c3
Relative Abundance
m/z
151.0940
$C_6{}^{13}C_4H_{11}O$ = 151.0939
0.9840 ppm
428.2518
250.1704
$C_{11}{}^{13}C_5H_{21}O_2$ = 250.1704
0.1798 ppm
576.3173
278.1659
396.2269
218.1451
446.2602
590.3347
336.2065
366.2198
475.2594
137.1149
123.0995
507.2860

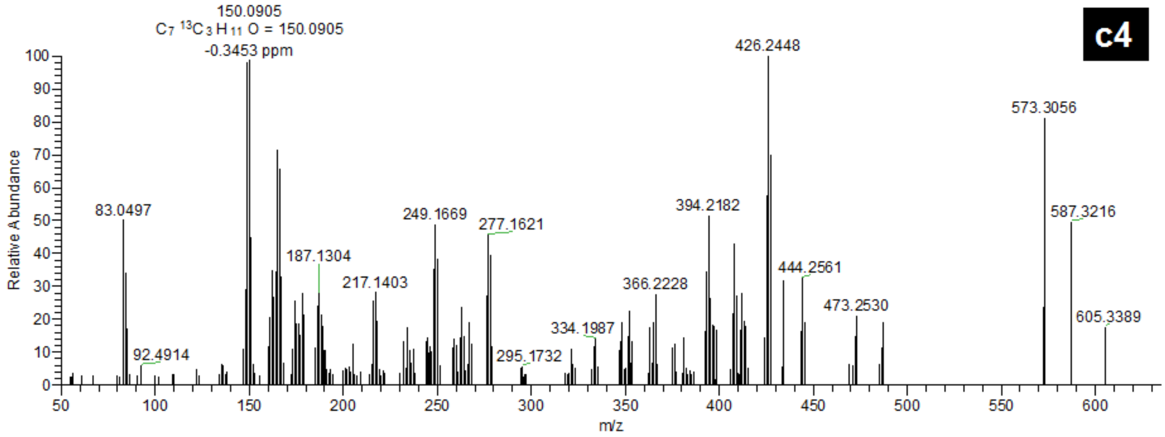
c4
150.0905
C7 13C3 H11 O = 150.0905
-0.3453 ppm
426.2448
573.3056
83.0497
92.4914
187.1304
217.1403
249.1669
277.1621
295.1732
334.1987
366.2228
394.2182
444.2561
473.2530
587.3216
605.3389
Relative Abundance
m/z

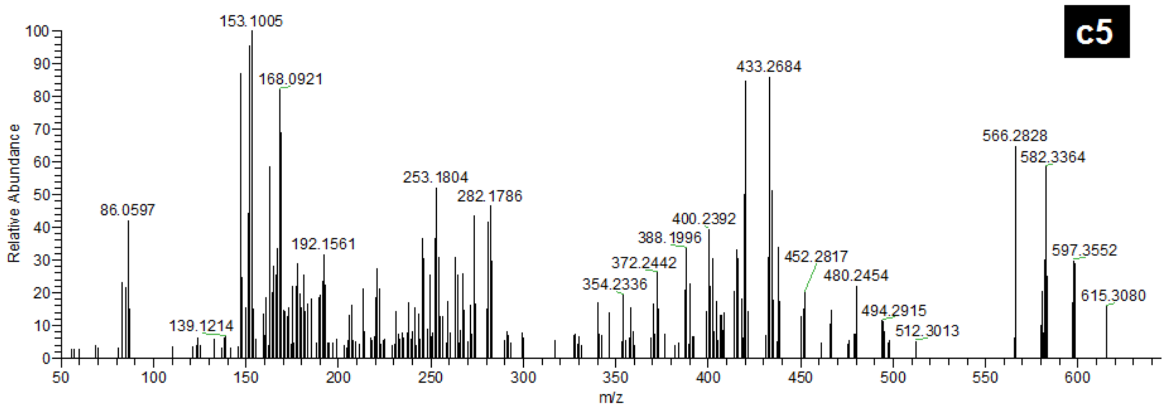
c5
153.1005
168.0921
86.0597
139.1214
192.1561
253.1804
282.1786
354.2336
372.2442
388.1996
400.2392
433.2684
452.2817
480.2454
494.2915
512.3013
566.2828
582.3364
597.3552
615.3080
Relative Abundance
m/z

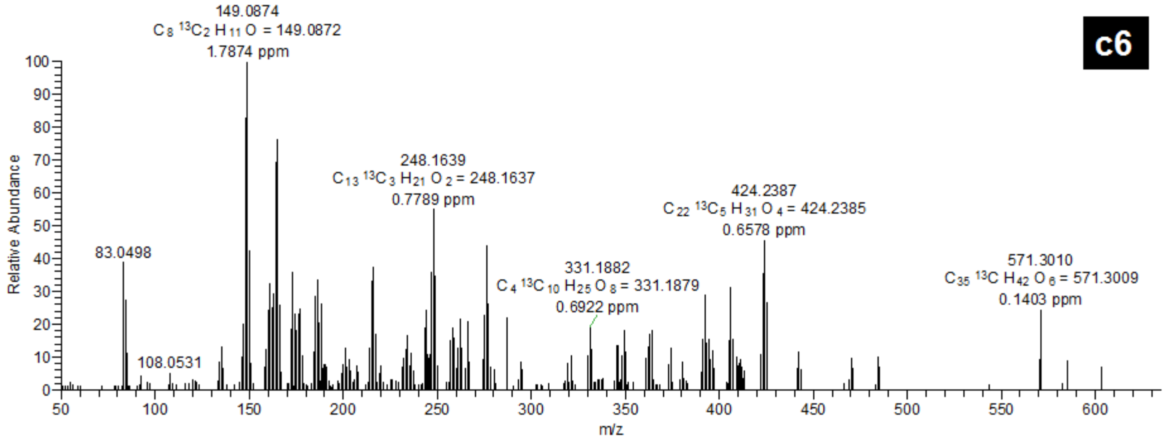
c6
149.0874
C8 13C2 H11 O = 149.0872
1.7874 ppm
83.0498
108.0531
248.1639
C13 13C3 H21 O2 = 248.1637
0.7789 ppm
331.1882
C4 13C10 H25 O8 = 331.1879
0.6922 ppm
424.2387
C22 13C5 H31 O4 = 424.2385
0.6578 ppm
571.3010
C35 13C H42 O6 = 571.3009
0.1403 ppm
Relative Abundance
m/z

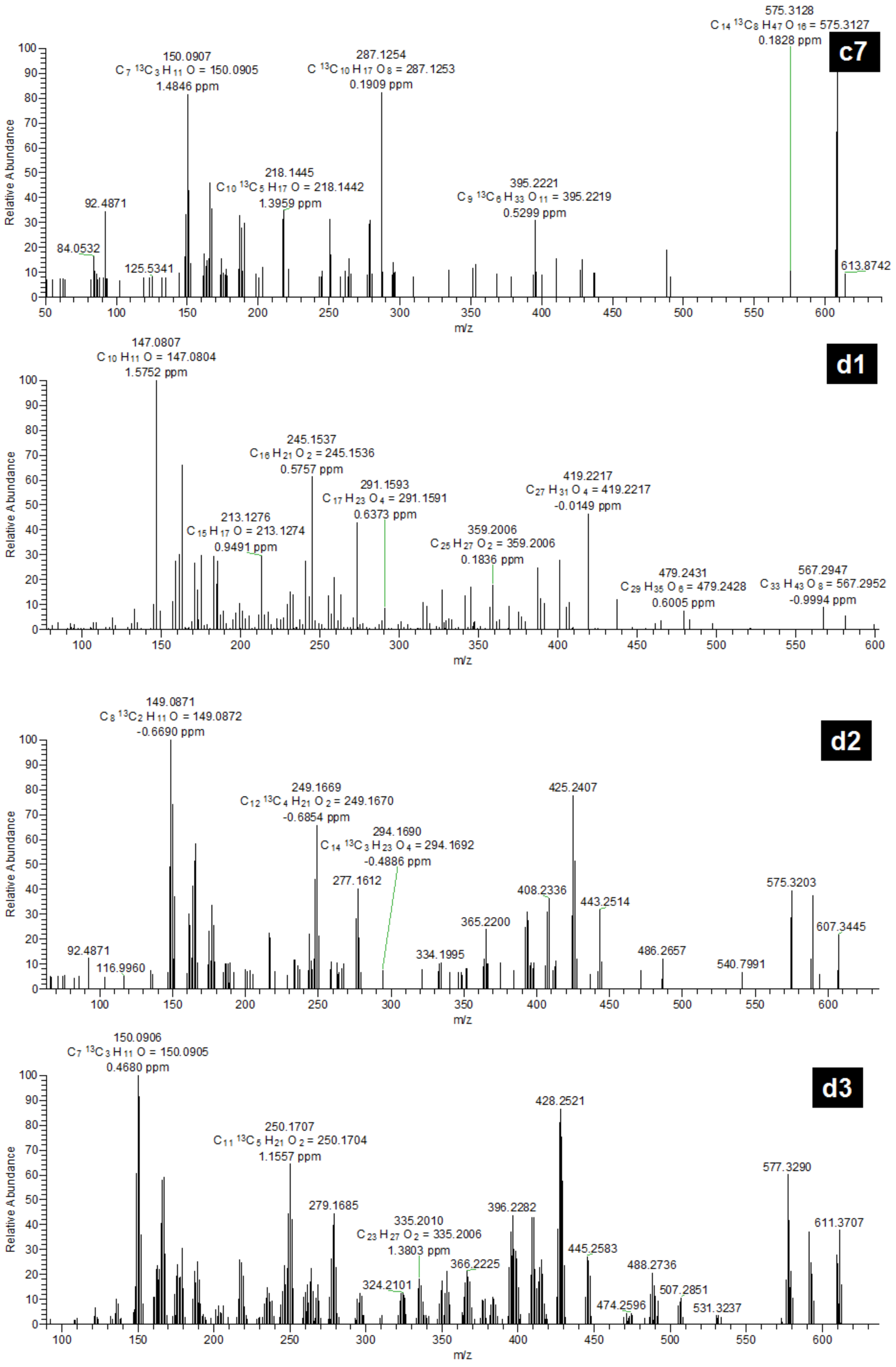
c7
575.3128
C 14 13C 8 H 47 O 16 = 575.3127
0.1828 ppm
150.0907
C 7 13C 3 H 11 O = 150.0905
1.4846 ppm
287.1254
C 13C 10 H 17 O 8 = 287.1253
0.1909 ppm
218.1445
C 10 13C 5 H 17 O = 218.1442
1.3959 ppm
395.2221
C 9 13C 6 H 33 O 11 = 395.2219
0.5299 ppm
92.4871
84.0532
125.5341
613.8742
Relative Abundance
m/z
d1
147.0807
C 10 H 11 O = 147.0804
1.5752 ppm
245.1537
C 16 H 21 O 2 = 245.1536
0.5757 ppm
291.1593
C 17 H 23 O 4 = 291.1591
0.6373 ppm
419.2217
C 27 H 31 O 4 = 419.2217
-0.0149 ppm
213.1276
C 15 H 17 O = 213.1274
0.9491 ppm
359.2006
C 25 H 27 O 2 = 359.2006
0.1836 ppm
479.2431
C 29 H 35 O 6 = 479.2428
0.6005 ppm
567.2947
C 33 H 43 O 8 = 567.2952
-0.9994 ppm
Relative Abundance
m/z
d2
149.0871
C 8 13C 2 H 11 O = 149.0872
-0.6690 ppm
249.1669
C 12 13C 4 H 21 O 2 = 249.1670
-0.6854 ppm
294.1690
C 14 13C 3 H 23 O 4 = 294.1692
-0.4886 ppm
425.2407
277.1612
408.2336
443.2514
575.3203
607.3445
365.2200
334.1995
486.2657
540.7991
92.4871
116.9960
Relative Abundance
m/z
d3
150.0906
C 7 13C 3 H 11 O = 150.0905
0.4680 ppm
250.1707
C 11 13C 5 H 21 O 2 = 250.1704
1.1557 ppm
428.2521
577.3290
279.1685
335.2010
C 23 H 27 O 2 = 335.2006
1.3803 ppm
396.2282
611.3707
445.2583
366.2225
488.2736
324.2101
474.2596
507.2851
531.3237
Relative Abundance
m/z

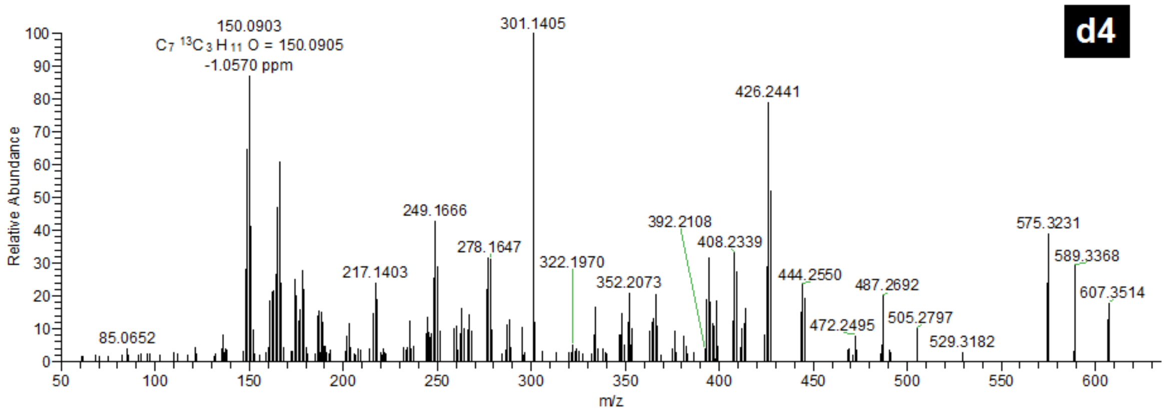
d4
Relative Abundance
m/z
150.0903
C7 13C3 H11 O = 150.0905
-1.0570 ppm
301.1405
426.2441
249.1666
278.1647
322.1970
352.2073
392.2108
408.2339
217.1403
85.0652
444.2550
472.2495
487.2692
505.2797
529.3182
575.3231
589.3368
607.3514

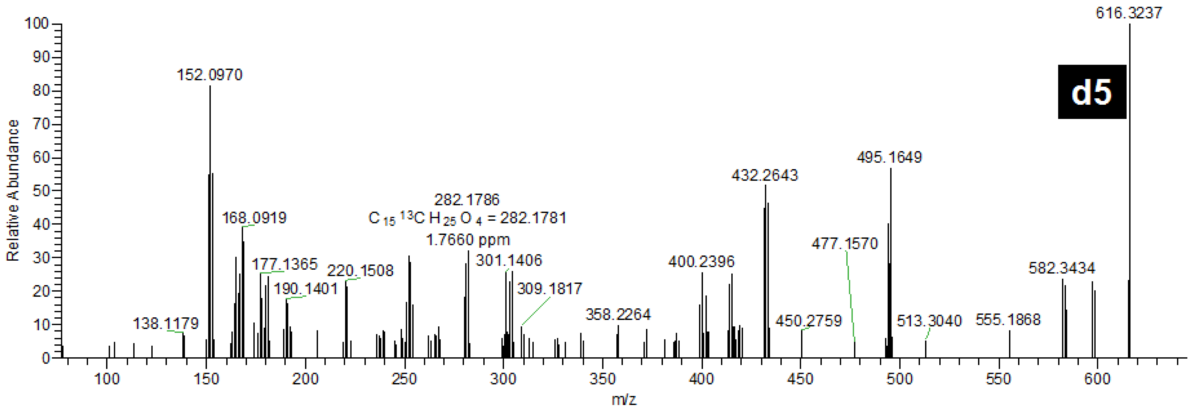
d5
Relative Abundance
m/z
616.3237
152.0970
168.0919
177.1365
190.1401
220.1508
282.1786
C15 13C H25 O4 = 282.1781
1.7660 ppm
301.1406
309.1817
138.1179
358.2264
400.2396
432.2643
450.2759
477.1570
495.1649
513.3040
555.1868
582.3434

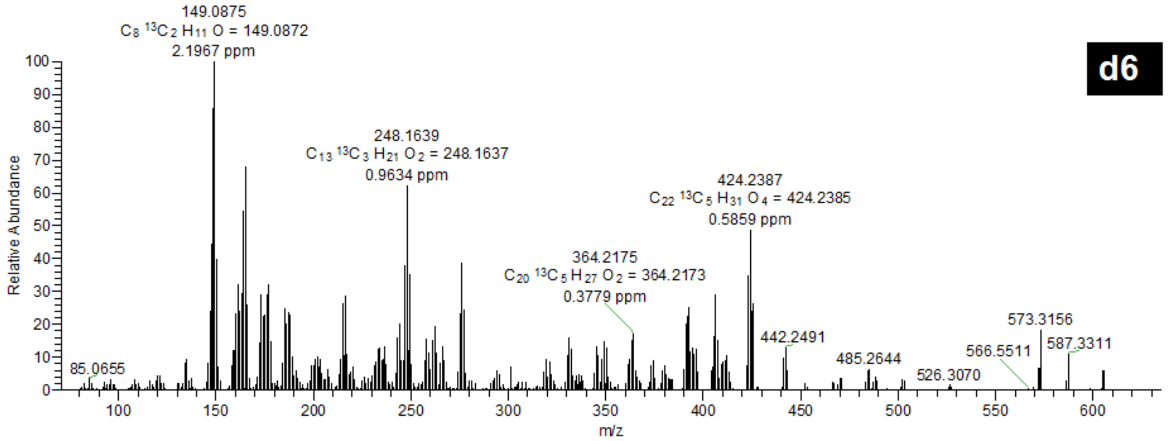
d6
Relative Abundance
m/z
149.0875
C8 13C2 H11 O = 149.0872
2.1967 ppm
248.1639
C13 13C3 H21 O2 = 248.1637
0.9634 ppm
424.2387
C22 13C5 H31 O4 = 424.2385
0.5859 ppm
364.2175
C20 13C5 H27 O2 = 364.2173
0.3779 ppm
85.0655
442.2491
485.2644
526.3070
566.5511
573.3156
587.3311

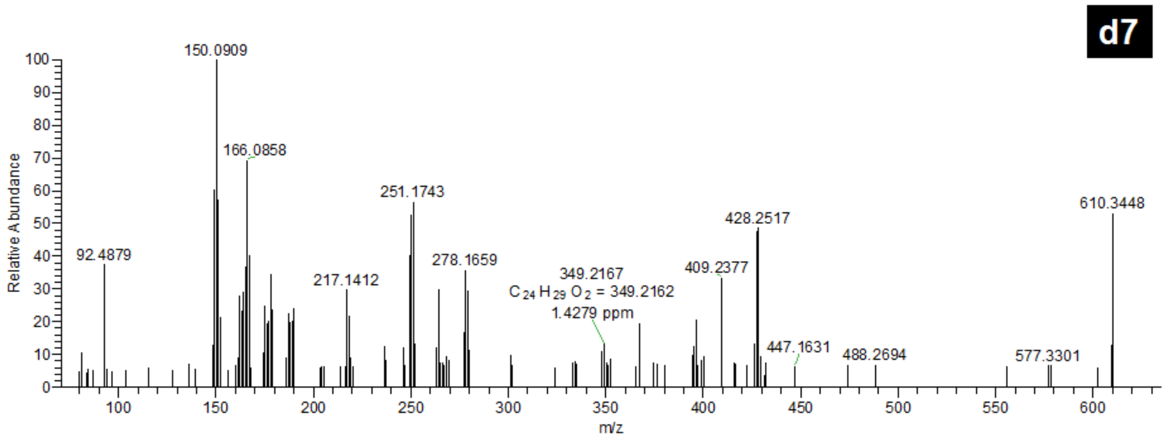
d7
Relative Abundance
m/z
150.0909
166.0858
251.1743
92.4879
217.1412
278.1659
349.2167
C24 H29 O2 = 349.2162
1.4279 ppm
409.2377
428.2517
447.1631
488.2694
577.3301
610.3448

Fig. S4. Comparison of MS/MS spectra of the unlabeled and ^{13}C labeled limonoids from the neem cell suspension. (a1) azadirachtin A. azadirachtin isotopologues obtained through 1-^{13}C Glc labeling. (a2) *m/z* 707. (a3) *m/z* 713. azadirachtin isotopologues obtained through 1,6-^{13}C Glc labeling. (a4) *m/z* 710. (a5) *m/z* 717; azadirachtin isotopologues obtained through 2-^{13}C Glc labeling (a6) *m/z* 706; (a7) *m/z* 716. (b1) 6-deacetylnimbinene. 6-deacetylnimbinene isotopologues obtained through 1-^{13}C Glc labeling (b2) *m/z* 446. (b3) *m/z* 452. 6-deacetylnimbinene isotopologues obtained through 1,6-^{13}C Glc labeling. (b4) *m/z* 447. (b5) *m/z* 452. 6-deacetylnimbinene isotopologues obtained through 2-^{13}C Glc labeling. (b6) *m/z* 445. (b7) *m/z* 450. (c1) salannin. salannin isotopologues obtained through 1-^{13}C Glc labeling (c2) *m/z* 603. (c3) *m/z* 610. salannin isotopologues obtained through 1,6-^{13}C Glc labeling. (c4) *m/z* 605. (c5) *m/z* 615. salannin isotopologues obtained through 2-^{13}C Glc labeling (c6) *m/z* 603. (c7) *m/z* 608. (d1) salannolacetate. salannolacetate isotopologues obtained through 1-^{13}C Glc labeling (d2) *m/z* 607. (d3) *m/z* 611. salannolacetate isotopologues obtained through 1,6-^{13}C Glc labeling (d4) *m/z* 607. (d5) *m/z* 616. salannolacetate isotopologues obtained through 2-^{13}C Glc labeling (d6) *m/z* 605. (d7) *m/z* 610.

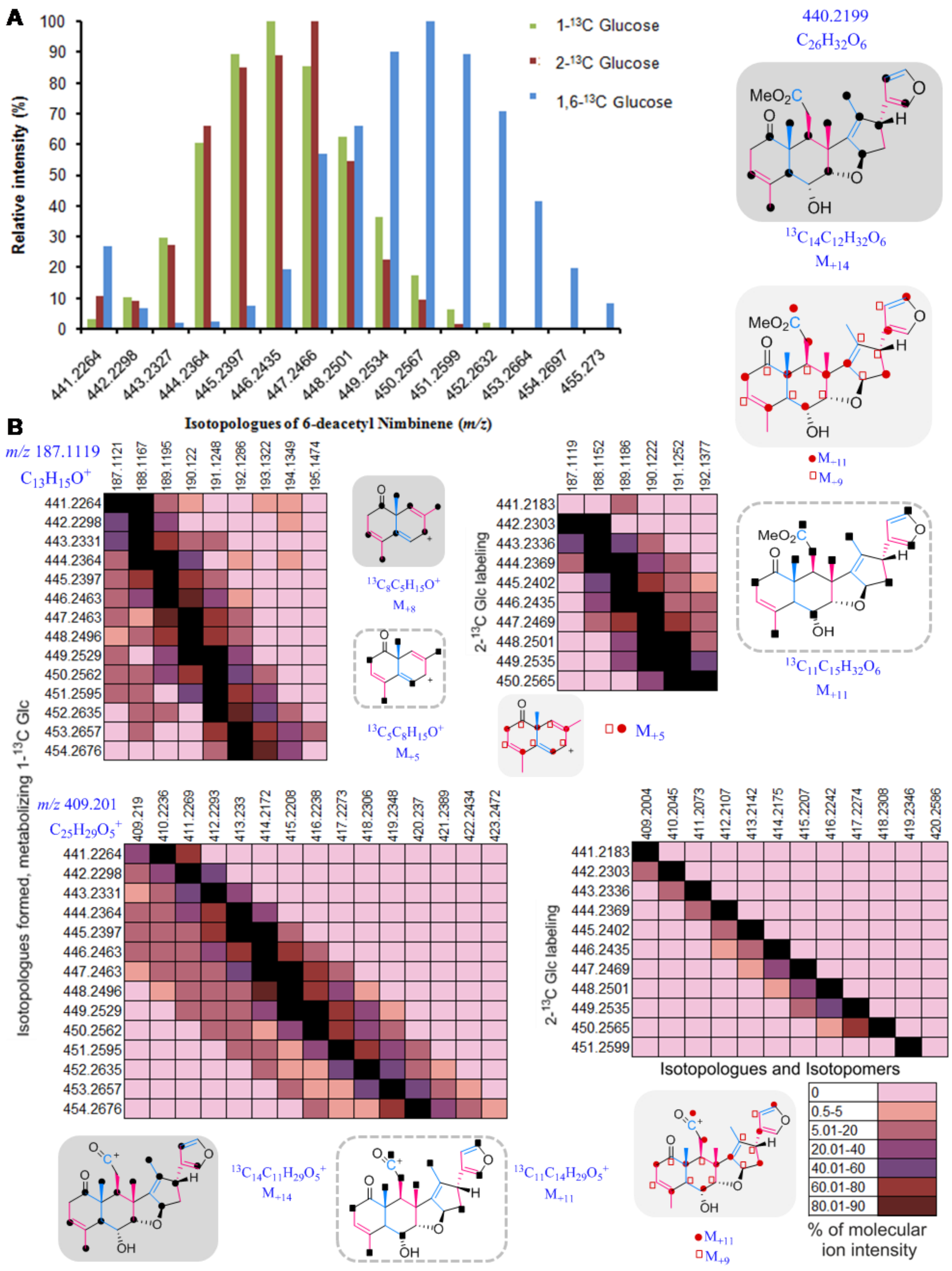

Fig. S5. Comparison of relative intensity of isotopologues obtained with the uptake of different ^{13}C-glucose tracers. (a) MS of 6-deacetylnimbinene isotopologues (b) Heatmap for distribution of isotopologues for specific fragments (*m/z* 187.1119, 409.2010 contributing to different part of skeleton respectively) for each 6-deacetylnimbinene isotopologues obtained from [1-^{13}C] Glc (left) and [2-^{13}C] Glc (right) labeling experiments. (M represents the *m/z* of parent ion of the molecular ion/ fragment)

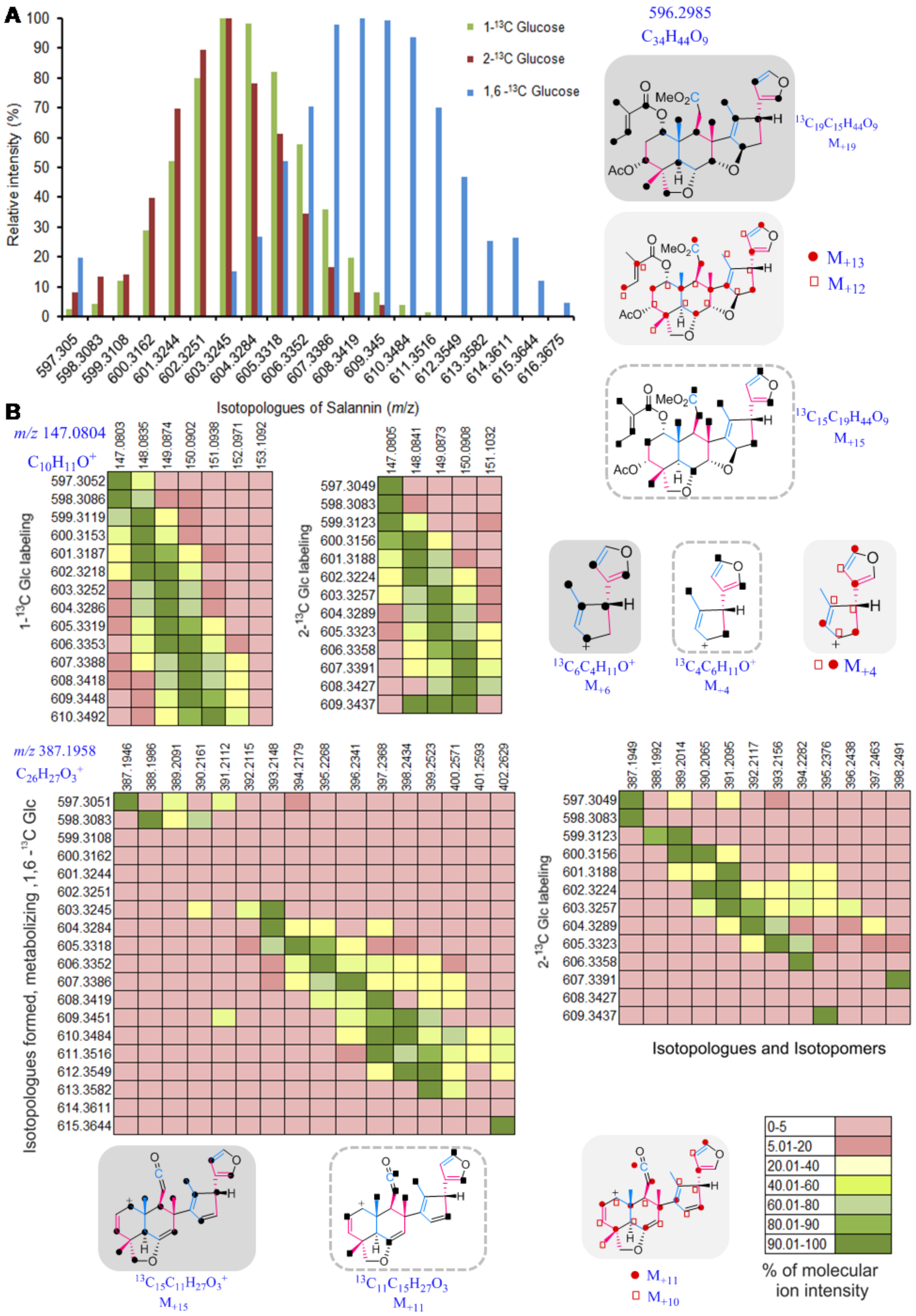

Fig. S6. Relative intensity of salannin isotopologues obtained from labeling study with different tracers such as [1-^{13}C], [2-^{13}C], [1,6-^{13}C] Glc. (a) MS of salannin isotopologues

formed from three different Glc tracers (b) MS/MS distribution of isotopologues of the fragments (shown here are *m/z* 147.0804 and 387.1946 representing the skeleton). (M represents the *m/z* of parent ion of the molecular ion/ fragment) (heatmap at left corresponds to isotopologues formed through [1-^{13}C]/ [1,6-^{13}C] Glc labeling and for the [2-^{13}C] Glc labeling at the right).

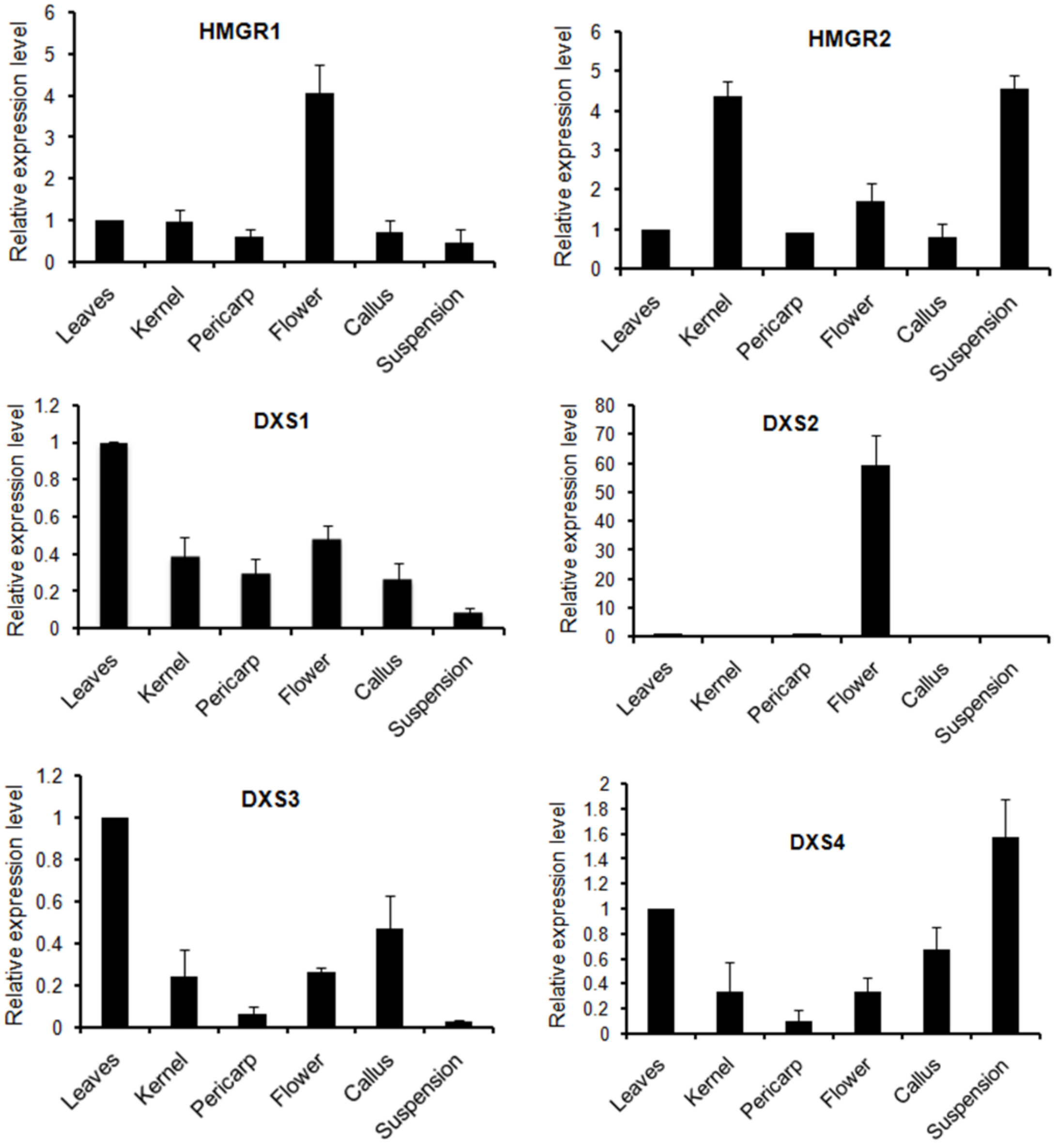

Fig. S7. Relative expression level of genes of MVA and MEP pathway encoding for the rate limiting enzymes in different neem tissues. HMGR, 3-hydroxy-3-methyl-glutaryl-coenzyme A reductase; DXS, 1-deoxy-D-xylulose-5-phosphate synthase.

Molecule	Molecular mass	Precursor ion (*m/z*)	Molecular formula of precursor ion	Adduct	Daughter ion *m/z* (formula, Relative intensity) at NCE 20%
**Azadirachtin A**	720.2629	703.2604	$C_{35}H_{43}O_{15}$	M-H_2O+H	83.0497 (C_5H_7O, 76.41), 161.0596 ($C_{10}H_9O_2$, 100), 241.0859 ($C_{15}H_{13}O_3$, 26.71), 269.0809 ($C_{16}H_{13}O_4$, 54.72), 329.1016 ($C_{18}H_{17}O_6$, 43.8), 447.1433 ($C_{26}H_{23}O_7$, 50.04), 463.1374 ($C_{26}H_{23}O_8$, 28.9), 475.1392 ($C_{27}H_{23}O_8$, 40.55), 507.1650 ($C_{28}H_{27}O_9$, 85.66) 525.1754 ($C_{28}H_{29}O_{10}$, 30.37), 567.1860 ($C_{30}H_{31}O_{11}$, 62.22), 585.1970 ($C_{30}H_{33}O_{12}$, 38.73)
**Azadirachtin B**	662.2575	645.2548	$C_{33}H_{41}O_{13}$	M-H_2O+H	83.0497 (C_5H_7O, 100), 161.0596 ($C_{10}H_9O_2$, 40.63), 179.0701 ($C_{10}H_{11}O_3$, 12.54), 253.0857 ($C_{16}H_{13}O_3$, 5.14), 271.0962 ($C_{16}H_{15}O_4$, 13.18), 287.0910 ($C_{16}H_{15}O_5$, 10.29), 289.1066 ($C_{16}H_{17}O_5$, 79.39), 389.1372 ($C_{24}H_{21}O_5$, 6.72), 497.1801 ($C_{27}H_{29}O_9$, 12.2), 509.1799 ($C_{28}H_{29}O_9$, 7.51), 527.1910 ($C_{28}H_{31}O_{10}$, 6.38), 545.2030 ($C_{28}H_{33}O_{11}$,5.54), 643.2717 ($C_{34}H_{43}O_{12}$,)
**3-deacetyl Azadirachtin A**	678.2524	661.2498	$C_{33}H_{41}O_{14}$	M-H_2O+H	161.0598 ($C_{10}H_9O_2$, 26.55), 269.0809 ($C_{16}H_{13}O_4$, 15.61), 329.1013 ($C_{18}H_{17}O_6$, 12.55), 347.1122 ($C_{18}H_{19}O_7$, 13.85), 447.1436 ($C_{26}H_{23}O_7$, 10.45), 465.1543 ($C_{26}H_{25}O_8$, 11.62), 507.1655 ($C_{28}H_{27}O_9$, 26.66), 511.1612 ($C_{27}H_{27}O_{10}$, 10.58), 525.1758 ($C_{28}H_{29}O_{10}$, 100), 543.1863 ($C_{28}H_{31}O_{11}$, 27.78) 583.2184 ($C_{31}H_{35}O_{11}$, 13.91), 625.2281 ($C_{33}H_{37}O_{12}$, 11.85), 643.2382 ($C_{33}H_{39}O_{13}$, 13.16)
**Azadirachtin H**	662.2575	645.2550	$C_{33}H_{41}O_{13}$	M-H_2O+H	83.0497 (C_5H_7O, 100), 183.0807 ($C_{13}H_{11}O$, 80.84), 203.0711 ($C_{12}H_{11}O_3$, 9.09), 205.0855 ($C_{12}H_{13}O_3$, 10.83), 211.0754 ($C_{14}H_{11}O_2$, 10.35), 213.0914 ($C_{14}H_{13}O_2$, 29.61), 233.0867 ($C_{13}H_{13}O_4$, 63.36), 241.0867 ($C_{15}H_{13}O_3$, 9.05), 243.1016 ($C_{15}H_{15}O_3$, 33.8), 259.0964 ($C_{15}H_{15}O_4$, 11.76), 271.0958 ($C_{16}H_{15}O_4$, 16.76), 273.1107 ($C_{16}H_{17}O_4$, 11.94), 303.1219 ($C_{17}H_{19}O_5$, 14.07), 333.1332 ($C_{18}H_{21}O_6$, 33.68),
		659.2704	$C_{34}H_{43}O_{13}$	M-H_2O+H	83.0497 (C_5H_7O, 77.85), 161.0598 ($C_{10}H_9O_2$, 100), 199.1120 ($C_{14}H_{15}O$, 92.87), 225.0910 ($C_{15}H_{13}O_2$, 59.32), 257.1169 ($C_{16}H_{17}O_3$, 27.93), 285.1118 ($C_{17}H_{17}O_4$, 57.89), 301.1069 ($C_{17}H_{17}O_5$, 31.81), 345.1337 ($C_{19}H_{21}O_6$, 29.89), 361.1282 ($C_{19}H_{21}O_7$, 28.38), 419.1480 ($C_{25}H_{23}O_6$,), 451.1749 ($C_{26}H_{27}O_7$, 23.41), 463.1750 ($C_{27}H_{27}O_7$, 82.35), 481.1859 ($C_{27}H_{29}O_8$, 32.99), 523.1965 ($C_{29}H_{31}O_9$, 62.58), 541.2070 ($C_{29}H_{3}3O_{10}$, 54.06)
**11-epi-azadirachtin D**	676.2731	641.2598	$C_{34}H_{41}O_{12}$	M-2H_2O+H	83.0497 (C_5H_7O, 100), 161.0597 ($C_{10}H_9O_2$, 67.67), 205.0858 ($C_{12}H_{13}O_3$, 14.62), 225.0910 ($C_{15}H_{13}O_2$, 48.31), 247.0963 ($C_{14}H_{15}O_4$, 48.31), 285.1117 ($C_{17}H_{17}O_4$, 56.13), 301.1068 ($C_{17}H_{17}O_5$, 17.22), 463.1748 ($C_{27}H_{27}O_7$, 51.27), 481.1855 ($C_{27}H_{29}O_8$, 27.55), 523.1962 ($C_{29}H_{31}O_9$, 29.92), 541.2068 ($C_{29}H_{33}O_{10}$, 57.04), 581.2377 ($C_{32}H_{37}O_{10}$, 21.98),
**Vepaol**	752.2892	735.2867	$C_{36}H_{47}O_{16}$	M-H_2O+H	83.0497 (C_5H_7O, 100), 123.0444 ($C_7H_7O_2$, 79.24), 185.0598 ($C_{12}H_9O_2$, 18.38), 203.0704 ($C_{12}H_{11}O_3$, 21.48), 205.0860 ($C_{12}H_{13}O_3$, 21.65), 217.0864 ($C_{13}H_{13}O_3$, 17.10), 231.0650 ($C_{13}H_{11}O_4$,20.49),

					233.0810 ($C_{13}H_{13}O_4$, 17.55), 249.0758 ($C_{13}H_{13}O_5$, 19.52),251.0916 ($C_{13}H_{15}O_5$, 32.88), 261.0763 ($C_{14}H_{13}O_5$, 22.02), 291.0863 ($C_{15}H_{15}O_6$, 79.16), 525.1750 ($C_{28}H_{29}O_{10}$, 16.77), 543.1869 ($C_{28}H_{31}O_{11}$, 14.3),
		721.2705	$C_{35}H_{45}O_{15}$	M-MeOH+H	83.0497 (C_5H_7O, 84.84), 161.0597 ($C_{10}H_9O_2$, 49.64), 183.0806 ($C_{13}H_{11}O$, 26.3), 241.0859 ($C_{15}H_{13}O_3$, 69.12), 269.0808 ($C_{16}H_{13}O_4$, 100), 283.0963 ($C_{17}H_{15}O_4$, 35.35), 287.0914 ($C_{16}H_{15}O_5$, 19.94), 301.1068 ($C_{17}H_{17}O_5$, 38.89), 329.1019 ($C_{18}H_{17}O_6$, 76), 347.1130 ($C_{18}H_{19}O_7$, 16.57), 389.1236 ($C_{20}H_{21}O_8$, 22.4), 507.1656 ($C_{28}H_{27}O_9$, 26.83), 525.1763 ($C_{28}H_{29}O_{10}$, 20.16), 567.1869 ($C_{30}H_{31}O_{11}$, 21.26), 585.1972 ($C_{30}H_{33}O_{12}$, 87.62).
		703.2598	$C_{35}H_{43}O_{15}$	M-MeOH-H_2O+H	83.0497 (C_5H_7O, 100), 123.0444 ($C_7H_7O_2$, 46.11), 161.0599 ($C_{10}H_9O_2$, 21.81), 203.0704 ($C_{12}H_{11}O_3$, 13.94), 205.0860 ($C_{12}H_{13}O_3$, 18.84), 231.0652 ($C_{13}H_{11}O_4$, 18.34), 251.0916 ($C_{13}H_{15}O_5$, 19.28), 261.0758($C_{14}H_{13}O_5$, 17.64), 269.0809 ($C_{16}H_{13}O_4$, 26.39), 291.0865 ($C_{15}H_{15}O_6$, 45.91), 329.1020 ($C_{18}H_{17}O_6$, 17.55), 447.1444 ($C_{26}H_{23}O_7$, 12.11), 507.1656 ($C_{28}H_{27}O_9$, 18.93), 525.1758 ($C_{28}H_{29}O_{10}$, 15.57), 543.1863 ($C_{28}H_{31}O_{11}$, 12.53), 585.1972 ($C_{30}H_{33}O_{12}$, 17.47)

Table S1. Azadirachtin derivatives, precursor ion, MS/MS daughter ions and intensity of fragments obtained from fragmentation of the precursor ion at 20% NCE.

**Gene name**	**Forward primer (5′–3′)**	**Reverse primer (5′–3′)**	**Amplicon**
Actin	AGGCATCCACGAGACCACTT	TGGCGCTAGAGCAGAAATTTC	151 bp
EF4a	AACCAGTGTTCGTGAAGACCAA	AAGCATTTCATCCGCCTCAT	151 bp
HMGR1	GGAAGCCGTCAATGATGGGA	ATTCAGGCACGCAGACTGAG	112 bp
HMGR2	TACTGCCATCTACTTAGCCACCG	CCCAACTGTGCCAACCTCAATAGA	145 bp
DXS1	ATGGTTGCCACTGCTGCCAG	CTCTACAAGAACTTTCCCTTTTCCAATCTC	135 bp
DXS2	AGAGAAATGGAGCGTGGTTCA	TAAGTCTAGGTCCTGCGGCTGG	145 bp
DXS3	TGACTGGGAGAAGAGATAAGATGC	CAGCAGAAATAGTGGTAGAACTGT	126 bp
DXS4	CAATGGGAGGTGGCACTGGTC	TGGCTTGAGACCTTCCGTAGC	128 bp

Table S2. List of primers used in this study for real time PCR analysis.
